# Supplementary material for: Sox8 is essential for vertebrate gastrulation
Source: EMBO Rep. 2025 Nov 10;26(24):6179–208. doi: 10.1038/s44319-025-00617-z (PMC12715262; doi:10.1038/s44319-025-00617-z)
Supplement: Supplementary file 1 — Table EV1 [file 44319_2025_617_MOESM1_ESM.docx]

**Table EV1:** Primers used in this study

| Number | Primer name | Primer sequence | Purpose | Ta | Product size | References |
| --- | --- | --- | --- | --- | --- | --- |
| 1 | P1'_Kremen2S | ATGCCTTTAGGT  TGTCTCCTCCCA  GAAATGATAAACACAC | Cloning CDS Kremen2S  in pCS2+ (insert) | 72 ºC | 1317 bp | This study |
| 2 | P2'_Kremen2S | TTATAACAGTGAT  TTCATGGATGACT  GATTGACGCaggtg | Cloning CDS Kremen2S  in pCS2+ (insert) |  |  |  |
| 3 | P1_Kremen2S | TTATCATTTCTGGG  AGGAGACAACCTA  AAGGCATCAATCGA  TGTCCGGCGATCGC  TGGCCGGCCTT | Cloning CDS Kremen2S in pCS2+ (vector) |  | 4204 bp | This study |
| 4 | P2_Kremen2S | GTCAATCAGTCATC  CATGAAATCACTGT  TATAAGAATTCAAG  GCCTCTCGAGCCTCT  AGAACTAT | Cloning CDS Kremen2S in pCS2+ (vector) |  |  |  |
| 5 | P1'_BMP7.1L | ATGAATGTTCTGCAAAAGAACAAAACAGGTTCTGTTCTCC | Cloning CDS BMP7.1L  in pCS2+ (insert) |  | 1275 bp | This study |
| 6 | P2'_BMP7.1L | TTAGTGGCATCCACAGGCTCTGACCACCATGTTTCTGTAT | Cloning CDS BMP7.1L  in pCS2+ (insert) |  |  |  |
| 7 | P1_BMP7.1L | CAGAACCTGTTTTGTTCTTTTGCAGAACATTCATCAATCGATGTCCGGCGATCGCTGGCCGGCCTT | Cloning CDS BMP7.1L  in pCS2+ (vector) |  | 4206 bp | This study |
| 8 | P2_BMP7.1L | GAAACATGGTGGTCAGAGCCTGTGGATGCCACTAAGAATTCAAGGCCTCTCGAGCCTCTAGAACTAT | Cloning CDS BMP7.1L  in pCS2+ (vector) |  |  |  |
| Number | **Primer name** | **Primer sequence** | **Purpose** | **Ta** | **Product size** | **References** |
| 9 | DisCas7-11_T7+linker (universal primer) | TAATACGACTCACTATAGGTTGGAAAGCCGGTTTTCTTTGATGTCACGGAAC | CRISPR downregulation | 61 ºC | 75 bp | (Özcan *et al*, 2021) |
| 10 | G1_3’UTR DisCas7-11 | TGAAGGACTATAATGCCATAATAGTTCCGTGACATCAAAGAAAA | Downregulation of Sox8 |  |  | This study |
| 11 | G2_3’UTR DisCas7-11 | CCTGCTGATAAACTGAGGATTTTGTTCCGTGACATCAAAGAAAA | Downregulation of Sox8 |  |  |  |
| 12 | G3_3’UTR DisCas7-11 | ACCAGACCAACAATTCTGATTCTGTTCCGTGACATCAAAGAAAA | Downregulation of Sox8 | 61 ºC | 75 bp | This study |
| 13 | G1_Kremen2_  3’ UTR | TGTGGTGCCATTACATGGTCATTGTTCCGTGACATCAAAGAAAA | Downregulation of Kremen2S |  |  |  |
| 14 | G2_Kremen2_3’ UTR | GGCCTACAAACTATTTATTTTATGTTCCGTGACATCAAAGAAAA | Downregulation of Kremen2S |  |  |  |
| 15 | G3_Kremen2_3’ UTR | CTGTGGCAAATAGTGAACCTGTAGTTCCGTGACATCAAAGAAAA | Downregulation of Kremen2S |  |  |  |
| Number | **Primer name** | **Primer sequence** | **Purpose** | **Ta** | **Product size** | **References** |
| 16 | Kremen2_Probe_Fw | TGTTGGTGGAGATGCTGTGG | Antisense probe for ISH Kremen2 | 69 ºC | 800 bp | (Janesick *et al*, 2018) |
| 17 | Kremen2_Probe_Rv | gatctcgaTAATACGACTCACTATAGGGTCTGGAATCTGGAAGATGTGGA | Antisense probe for ISH Kremen2 |  |  |  |
| 18 | Wnt11b_Fw | ATGGCTCCGACCCGTCACTG | Antisense probe for Wnt11b | 67 ºC | 841bp | This study |
| 19 | Wnt11b_T7_Rv | gatctcgaTAATACGACTCACTATAGGGagtgCCACCATGGACTGGCCTGATGTCTAGCTC | Antisense probe for Wnt11b |  |  |  |
| 20 | *bmp7.1_Fw* | ctcctttggacatacttctt | Antisense probe for *bmp7.1* | 59º C | 773 bp | This study |
| 21 | *bmp7.1_T7_Rev* | gatctcgaTAATACGACTCACTATAGGGagtgCCACCATGGatgaagggctgtttattgtgc | Antisense probe for *bmp7.1* |  |  |  |
| 22 | Sox8L_Fw | CTCACGTTTCGGACTCAGAC | SqRT-PCR Sox8 | 66 ºC | 481 bp | This study |
| 23 | Sox8L_Rv | GAGTCACTTTGCCCTGCCTT | SqRT-PCR Sox8 |  |  |  |

| Number | Primer name | Primer sequence | Purpose | Ta | Product size | References |
| --- | --- | --- | --- | --- | --- | --- |
| 24 | EF1α_Fw | ACCCTCCTCTTGGTCGTTTT | SqRT-PCR EF1α | 66 ºC | 134 bp | (Hong & Saint-Jeannet, 2007) |
| 25 | EF1α_Rv | TTTGGTTTTCGCTGCTTTCT | SqRT-PCR EF1α |  |  |  |
| 26 | dkk1_Fw | GCAAGATCAAGAACGCTTCC | SqRT-PCR dkk1 | 65 ºC | 206bp | This study |
| 27 | dkk1_Rv | GACGGGCACAACATAGACCT | SqRT-PCR dkk1 |  |  |  |
| 28 | Nodal 3.1 | TAATCTGTTGTGCCGATCCA | SqRT-PCR Nodal3.1 | 61 ºC | 152 bp | (Kofron et al, 2004) |
| 29 | Nodal 3.1 | ATCAATGTTGCCCTTTTTCA | SqRT-PCR Nodal3.1 |  |  |  |

| Number | Primer name | Primer sequence | Purpose | Ta | Product size | References |
| --- | --- | --- | --- | --- | --- | --- |
| 30 | xBra.Fw | GGAGTAATGAGTGCGACCGA | SqRT-PCR xBra | 68ºC | 243 bp | This study |
| 31 | xBra.Rv | GCCCGACATGCTCACCTTCA | SqRT-PCR xBra |  |  |  |
| 32 | Ventx1_Fw | CGCTCCCCCACCATATTAGG | SqRT-PCR Ventx1 | 65º C | 202 bp | This study |
| 33 | Ventx1_Rv | CAAAGAGTTGTGGGTAGGGT | SqRT-PCR Ventx1 |  |  |  |
| 34 | msfGFP_Fw | GTGAGTAAAGGTGAAGAACTC | SqRT-PCR msfGFP | 59 ºC | 520 bp | This study |
| 35 | msfGFP_Rv | GATCTTAAAGTTGGCCTTTATC | SqRT-PCR msfGFP |  |  |  |
| 36 | Kremen2_Fw | TGTTGGTGGAGATGCTGTGG | SqRT-PCR Kremen2 | 69 ºC | 778bp; 609bp (variant 1)  609bp (variant 1) | (Janesick *et al*, 2018)  (same primers as for the probe, but without the T7 promoter) |
| 37 | Kremen2_Rv | GGGTCTGGAATCTGGAAGATGTGGA | SqRT-PCR Kremen2 |  |  |  |

| Number | Primer name | Primer sequence | Purpose | Ta | Product size | References |
| --- | --- | --- | --- | --- | --- | --- |
| 38 | Sox8_CDS_  Fw | ATGCTGAACATGAGTTCGGATCAGGAGCCTCCCTGCAGCC | For Sox8-GFP  Cloning (insert) | 72º C | 1377 bp | This study |
| 39 | Sox8_CDS_Rv | CAAAGAGTTGTGGGTAGGGT | For Sox8-GFP  Cloning (insert) |  |  |  |
| 40 | Primer A_pCS2 | GGGAGGCTCCTGATCCGAACTCATGTTCAGCATCCGGCCTTTAATTAATGGCGCGCCACTAG | For Sox8-GFP  Cloning (vector) | 72 ºC | 4922 bp | This study |
| 41 | Primer A'_pCS2 | GGACCAGCCTGTCTACACAACCCTGACAAGGCCTCCAGCGATCGCCGGACATCGATTGATGAG | For Sox8-GFP  Cloning (vector) |  |  |  |
| 42 | PB1_P1 | CTCCTGGTTGCAAGGCATTTGCA | For ChIP-PCR | 67 ºC | 100 bp | This study |
| 43 | PB1_P2 | CACTCCTCCCCTTTTATAGTCCTTG | For ChIP-PCR |  |  |  |
| 44 | PBN_P1 | TCAAGATCCAAAACGAATATCTGATTAT | For ChIP-PCR | 62º C | 100 bp | This study |
| 45 | PBN_P2 | AGCCAGCTTAAGTCTTGGAA |  |  |  |  |
| Number | **Primer name** | **Primer sequence** | **Purpose** | **Ta** | **Product size** | **References** |
| 46 | P1_PCS2 for huDisCas7-11 | acggctcgaggaattcaattgaaatcttcatagtagtcgtcatACTAGTGGATCCTGCAAAAAGAACAAGTAGC | Gibson Assembly for huDisCas7-11 cloning in Pcs2+ | 72 ºC | 5009 bp | This study |
| 47 | P2_PCS2 for huDisCas7-11 | tcggcagaagaagctgaccacgccgtggacaccgtgggcaGGAGGAGGTGGAAGCGGAGGAGGAGGAAG |  |  |  |  |
| 48 | Primer  F_huDisCas7-11 | tgacgactactatgaagatttcaattgaattcctcgagccgt | Gibson Assembly for huDisCas7-11 cloning in Pcs2+ | 65 ºC | 4803 bp | This study |
| 49 | Primer  R_huDisCas7-11 | tgcccacggtgtccacggcgtggtcagcttcttctgccga | Gibson Assembly for huDisCas7-11 cloning in Pcs2+ |  |  |  |
