# Supplementary material for: Sox8 is essential for vertebrate gastrulation
Source: EMBO Rep. 2025 Nov 10;26(24):6179–208. doi: 10.1038/s44319-025-00617-z (PMC12715262; doi:10.1038/s44319-025-00617-z)
Supplement: Supplementary file 2 — Table EV2 [file 44319_2025_617_MOESM2_ESM.docx]

**Table EV2:** gRNAs used in this study

| Guide name | Guide sequence |
| --- | --- |
| G1_Sox8 3’UTR DisCas7-11 | GTTGATGTCACGGAACTATTATGGCATTATAGTCCTTCA |
| G2_Sox8 3’UTR DisCas7-11 | GTTGATGTCACGGAACAAAATCCTCAGTTTATCAGCAGG |
| G3_Sox8 3’UTR DisCas7-11 | GTTGATGTCACGGAACAGAATCAGAATTGTTGGTCTGGT |
| G1_Krm2 3’UTR DisCas7-11 | GTTGATGTCACGGAACAATGACCATGTAATGGCACCACA |
| G2_Krm2 3’UTR DisCas7-11 | GTTGATGTCACGGAACATAAAATAAATAGTTTGTAGGCC |
| G3_Krm2 3’UTR DisCas7-11 | GTTGATGTCACGGAACTACAGGTTCACTATTTGCCACAG |
