# Supplementary material for: Sox8 is essential for vertebrate gastrulation
Source: EMBO Rep. 2025 Nov 10;26(24):6179–208. doi: 10.1038/s44319-025-00617-z (PMC12715262; doi:10.1038/s44319-025-00617-z)
Supplement: Supplementary file 3 — Appendix [file 44319_2025_617_MOESM3_ESM.pdf]

Sox8 is essential for vertebrate gastrulation

Sofia Moreira[1,2], Artemis G. Korovesi[3] & Elias H Barriga[1,2*]

[1] Mechanisms of Morphogenesis Lab, Cluster of Excellence Physics of Life (PoL), TU Dresden, Dresden, Germany.

[2] Mechanisms of Morphogenesis Lab, Gulbenkian Institute of Science (IGC); Oeiras, Portugal.

[3] Patterning and Morphogenesis Lab, Gulbenkian Institute for Molecular Medicine (GIMM), Lisbon, Portugal

* Correspondence to Elias H Barriga
Email: elias.barriga@tu-dresden.de

Appendix:

Appendix Figure S1: Transcription factors, including SOX, are highly expressed in the ventral territory 2
Appendix Figure S2: Principal component analysis (PCA) of RNA-seq data from control and sox8 CRISPR Cas7-11 samples....... 3
Appendix Figure S3: ChIP–PCR results showing that Sox8 binds to the kremen2 promoter. 4
Appendix Figure S4: Step-by-step workflow of CRISPR Cas7-11 RNA targeting:....5
Appendix Figure S5: Fill-in-PCR schematic representation for generation of the guide PCR template. 6
Appendix Figure S6: Examples of gel images 7
Appendix Figure S7: Map of pCS2+ huDisCas7-11 msfGFP created with SnapGene® software 8
Appendix References....... 9

A

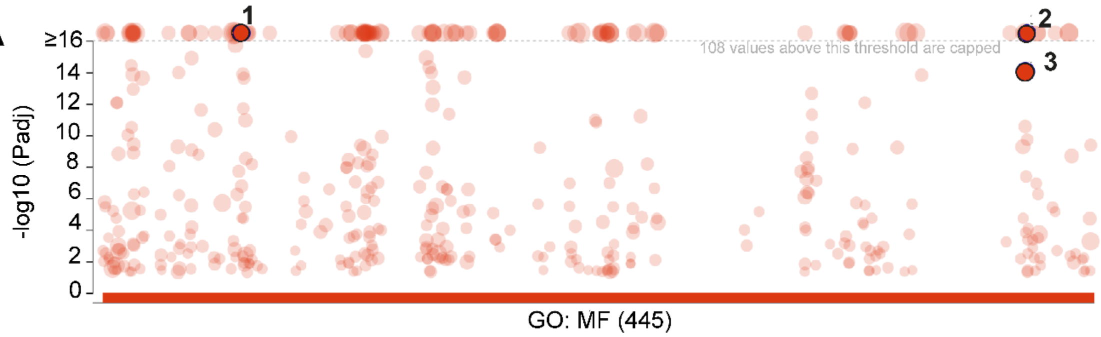

No.	Source	Term ID	Term name	Padj
1	GO:MF	GO:0008134	Transcription factor binding	4.902×10^{-66}
2	GO:MF	GO:0140297	DNA-binding transcription factor binding	1.308×10^{-54}
3	GO:MF	GO:0140110	Transcription regulator activity	9.095×10^{-15}

B

Ranking	Sox group	Gene	Expression pattern	Reference
1	SoxD	*sox13.S*	Posterior cardinal veins, intersomitic veins and developing heart	McGarey *et al.* 2010
2	SoxG	*sox15.L*	Neuroectoderm; Dorsal marginal zone	Satow R *et al.* 2006<br>Nitta KR *et al.* 2004
3	SoxC	*sox11.L*	Neuroectoderm; Mesoderm around blastopore	Cizelsky *et al.* 2013<br>Marchak A *et al.* 2017
4	SoxE	*sox8.L*	Neuroectoderm; Ventrolateral domain	O'Donnell *et al.* 2006<br>Hong CS *et al.* 2008
5	SoxB1	*sox3.S*	Neuroectoderm; Dorsal marginal zone	Rorick AM *et al.* 2007<br>de Almeida I *et al.* 2008

Appendix Figure S1: Transcription factors, including SOX, are highly expressed in the ventral territory. A) Manhattan plot of g: Profiler enrichment results using a list of genes found in the RNA-seq data from ventral tissues. In the Manhattan plot for MF (molecular function), significant results were obtained for the terms “transcription factor binding”, “DNA-binding transcription factor binding” and “transcription regulatory activity” (terms with a p value <0.05). The table below shows these three enriched terms and the statistical Padj value for each term. B) The five most enriched SOX transcription factors and expression patterns reported in previously published works (*sox13.s*: McGary *et al*, 2010; *sox15.*L:Satow *et al*, 2006; Nitta *et al*, 2004; *sox11. L*: Cizelsky *et al*, 2013; Marchak *et al*, 2017; *sox8*. L: O’Donnell *et al*, 2006; Hong *et al*, 2008; *sox3.s*: (Rorick *et al*, 2007; de Almeida *et al*, 2008).

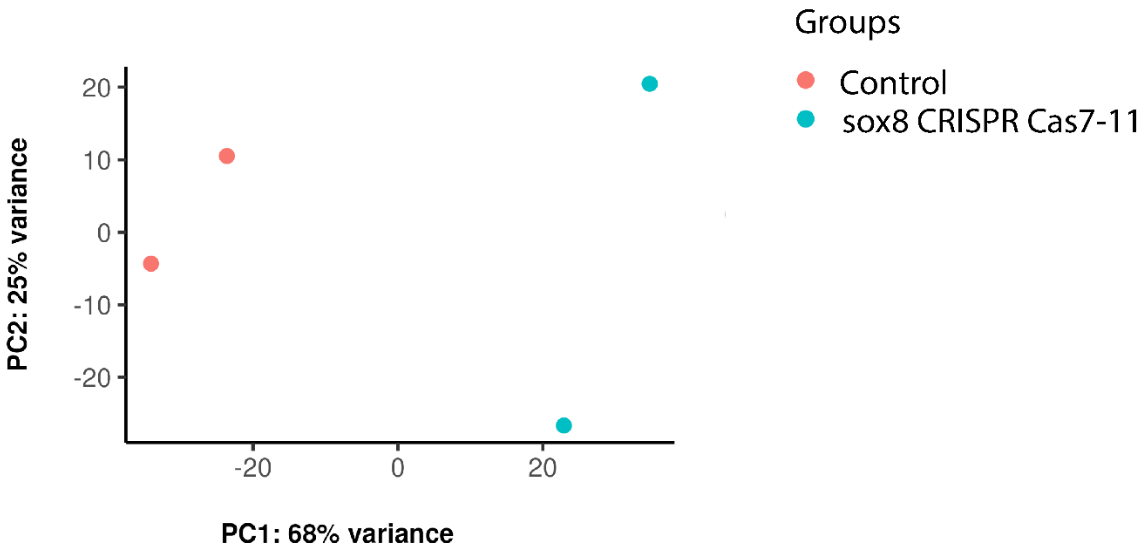

Appendix Figure S2: Principal component analysis (PCA) of RNA-seq data from control and sox8 CRISPR Cas7-11 samples. PCA was performed using variance-stabilized gene expression values from RNA-seq data, with a focus on the 500 most variable genes to illustrate the concordance between replicates and overall sample variability. Each point represents an individual biological replicate (n=2 per group), with control samples identified as red spots and *sox8* CRISPR as blue spots. The first two principal components (PC1 and PC2) account for 68% and 25% of the total variance, respectively. Separation along PC1 indicates substantial transcriptional changes induced by the *sox8* CRISPR treatment.

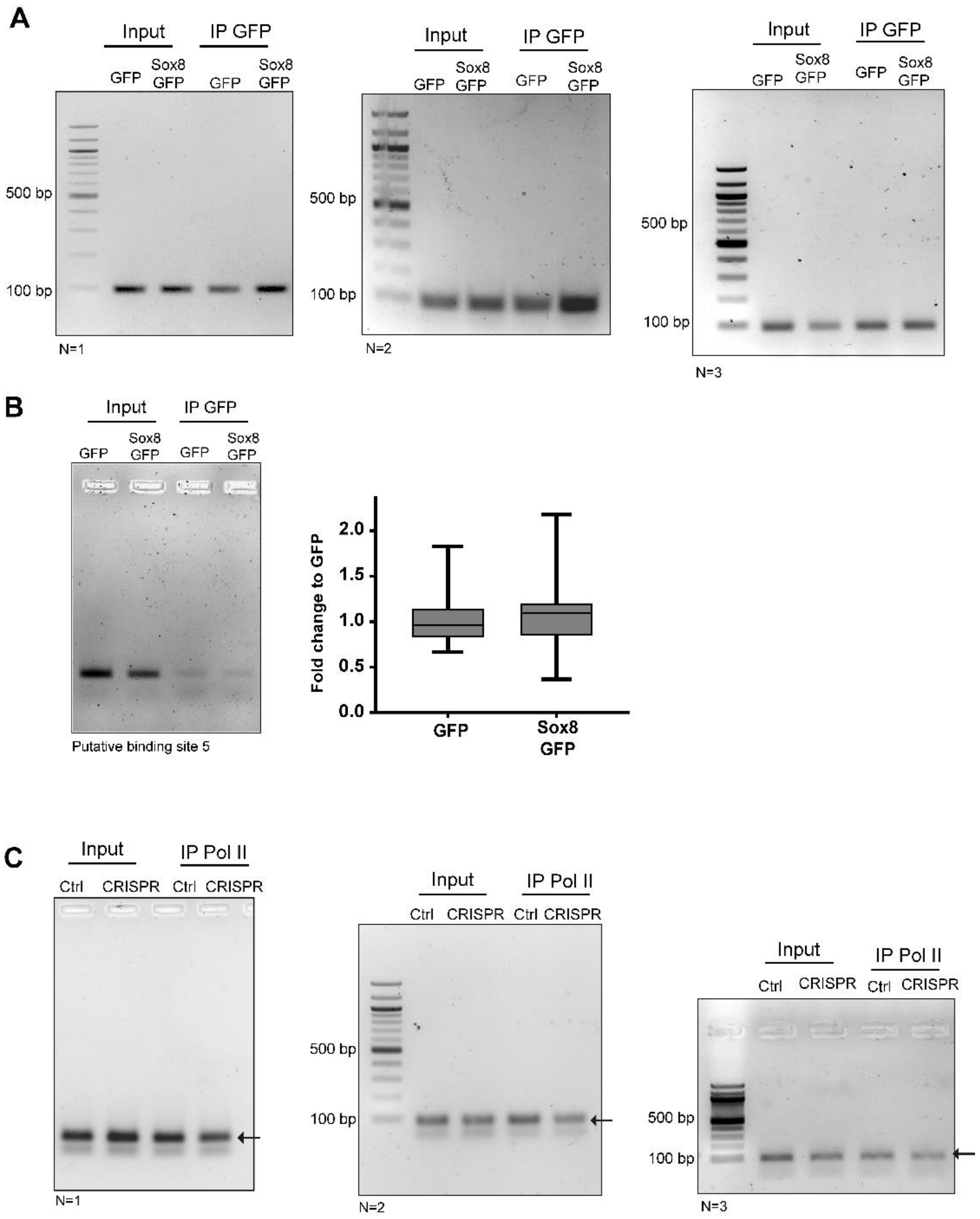

Appendix Figure S3: ChIP–PCR results showing that Sox8 binds to the kremen2 promoter. (A) Crops from the gels used to quantify the fold enrichment of Sox8-GFP binding to the putative binding site 1 of the kremen2 promoter; (B) Crops and quantifications of gels from PCRs using primers for other putative binding site and using the same ChIP-GFP samples as in A; (C) Crops from the gels used to quantify the percentage enrichment of Pol II bound to the kremen2 promoter under control and sox8 CRISPR Cas7-11 conditions. N=3 independent biological experiments.

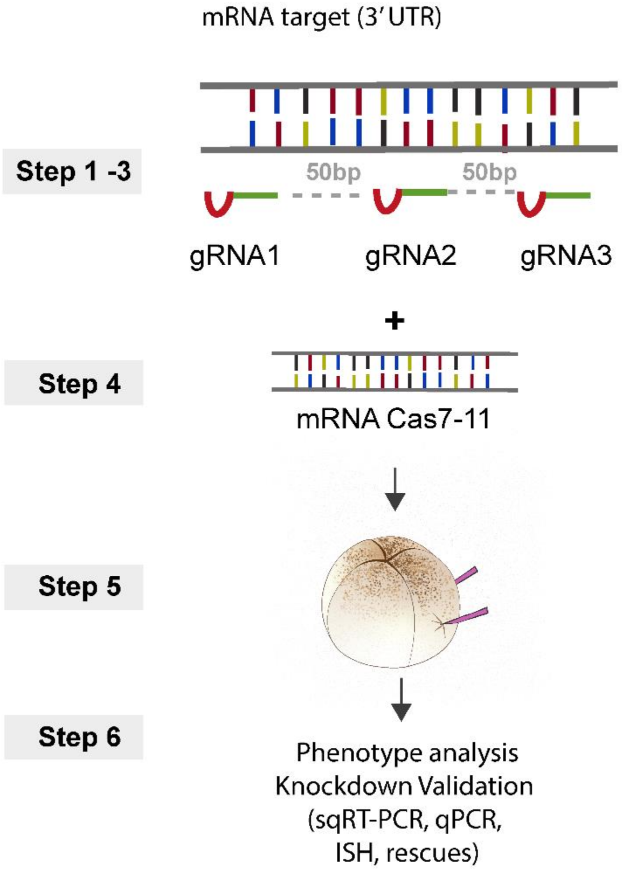

Appendix Figure S4: Step-by-step workflow of CRISPR Cas7-11 RNA targeting: from gRNA design (Steps 1-3) to mRNA Cas7-11 synthesis (Step 4), targeted injections (Step 5), phenotype analysis and knockdown validation (Step 6).

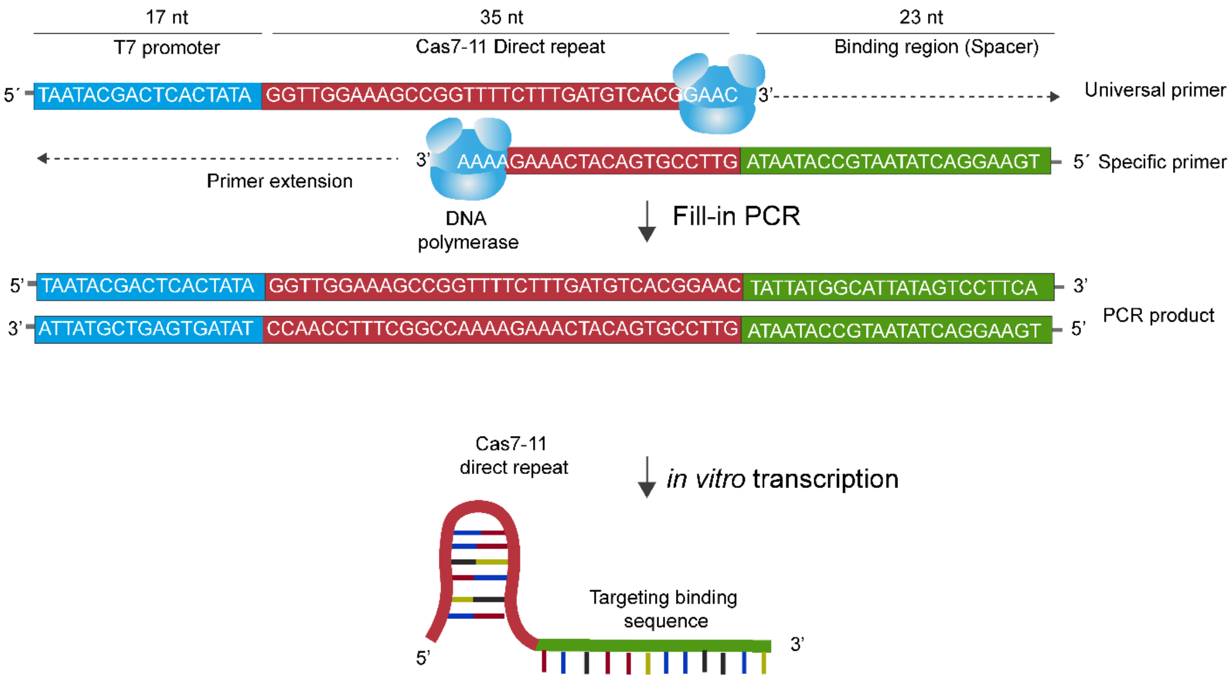

Appendix Figure S5: Fill-in-PCR schematic representation for generation of the guide PCR template. Universal primer (containing the 17 nucleotides of the T7 promoter and 35 nucleotides of the direct repeat for Cas7-11) and the specific primer (containing the reverse complement of part of the direct repeat sequence and a specific binding sequence of approximately 23 nucleotides) are represented. The direction of DNA polymerase extension is also indicated, as is the final PCR product, which will be used as a template for T7 RNA polymerase *in vitro* transcription.

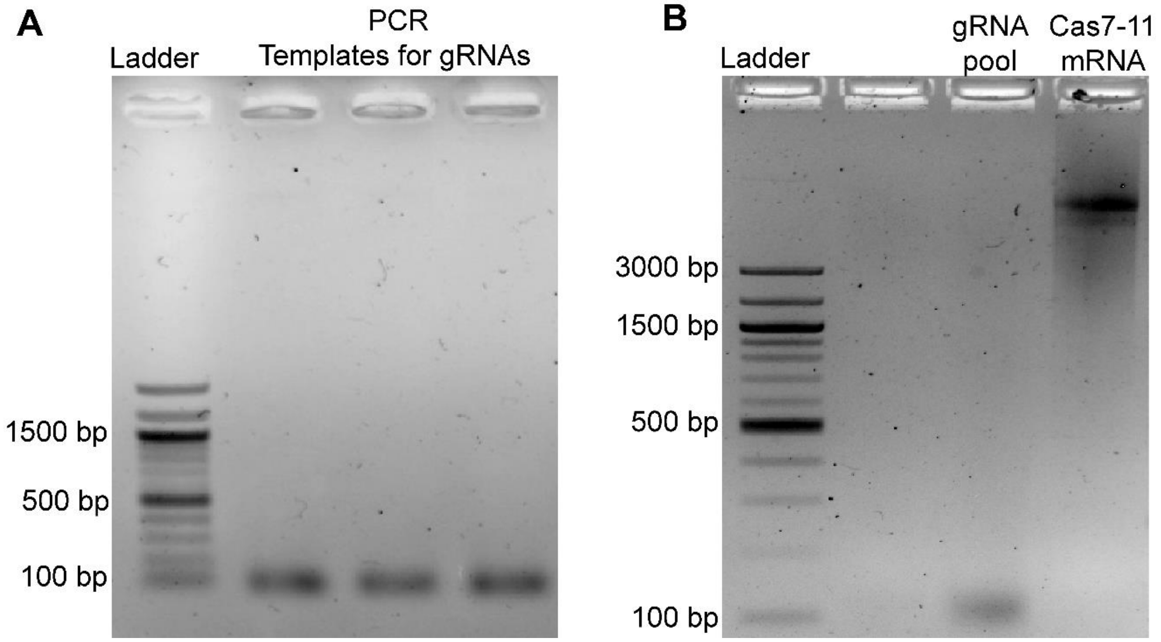

Appendix Figure S6: Examples of gel images. (A) gRNA PCR templates with expected sizes less than 100 bp and (B) the integrity of gRNA and mRNACas7-11 produced by *in vitro* transcription (2% agarose/1% bleach gel**).**

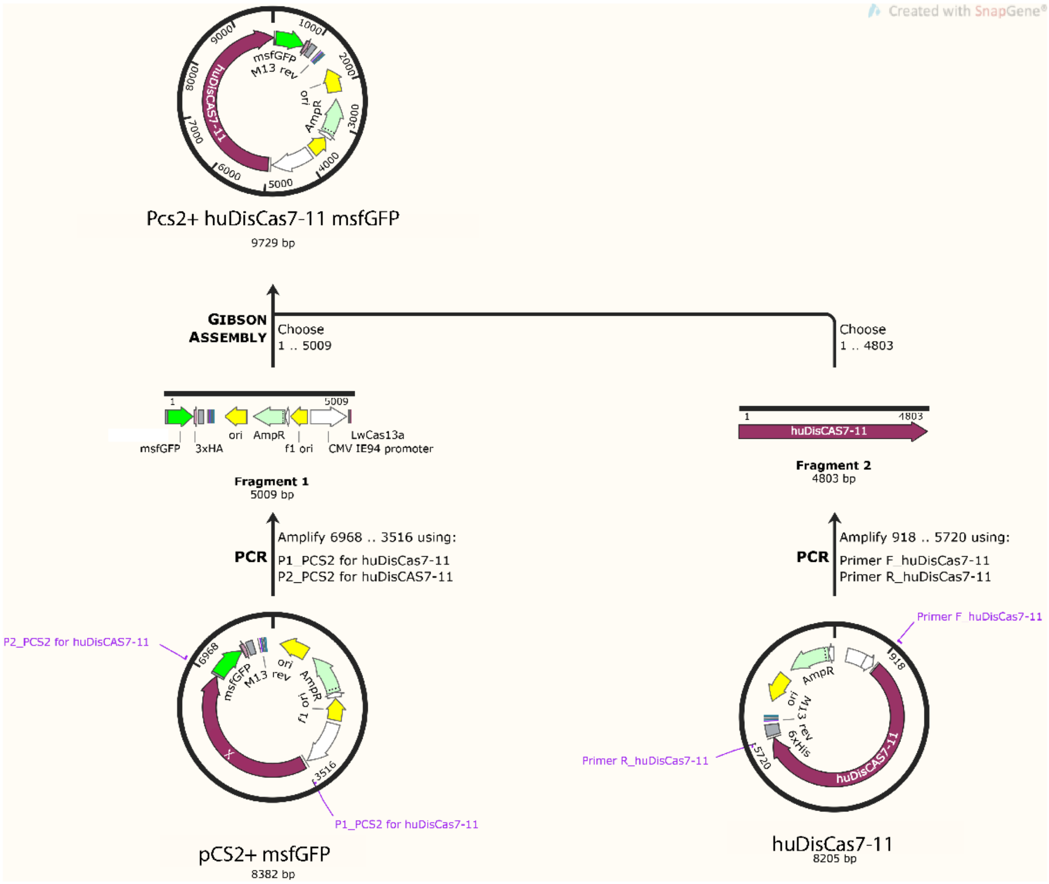

Appendix Figure S7: Map of pCS2+ huDisCas7-11 msfGFP created with SnapGene® software (from Dotmatics; available at snapgene.com)

Appendix References

de Almeida I, Rolo A, Batut J, Hill C, Stern CD & Linker C (2008) Unexpected activities of Smad7 in *Xenopus* mesodermal and neural induction. *Mechanisms of Development* 125: 421–431

Cizelsky W, Hempel A, Metzig M, Tao S, Hollemann T, Kühl M & Kühl SJ (2013) sox4 And sox11 Function during Xenopus laevis Eye Development. *PLOS ONE* 8: e69372

Hong C-S, Park B-Y & Saint-Jeannet J-P (2008) Fgf8a induces neural crest indirectly through the activation of Wnt8 in the paraxial mesoderm. *Development* 135: 3903–3910

Marchak A, Grant PA, Neilson KM, Datta Majumdar H, Yaklichkin S, Johnson D & Moody SA (2017) Wbp2nl has a developmental role in establishing neural and non-neural ectodermal fates. *Developmental Biology* 429: 213–224

McGary KL, Park TJ, Woods JO, Cha HJ, Wallingford JB & Marcotte EM (2010) Systematic discovery of nonobvious human disease models through orthologous phenotypes. *Proc Natl Acad Sci U S A* 107: 6544–6549

Nitta KR, Tanegashima K, Takahashi S & Asashima M (2004) *XSIP1* is essential for early neural gene expression and neural differentiation by suppression of BMP signaling. *Developmental Biology* 275: 258–267

O'Donnell M, Hong C-S, Huang X, Delnicki RJ & Saint-Jeannet J-P (2006) Functional analysis of Sox8 during neural crest development in *Xenopus*. *Development* 133: 3817–3826

Rorick AM, Mei W, Liette NL, Phiel C, El-Hodiri HM & Yang J (2007) PP2A:B56epsilon is required for eye induction and eye field separation. *Dev Biol* 302: 477–493

Satow R, Kurisaki A, Chan T, Hamazaki TS & Asashima M (2006) Dullard Promotes Degradation and Dephosphorylation of BMP Receptors and Is Required for Neural Induction. *Developmental Cell* 11: 763–774
