# Supplementary figures and images for: Sox8 is essential for vertebrate gastrulation
Source: EMBO Rep. 2025 Nov 10;26(24):6179–208. doi: 10.1038/s44319-025-00617-z (PMC12715262; doi:10.1038/s44319-025-00617-z)

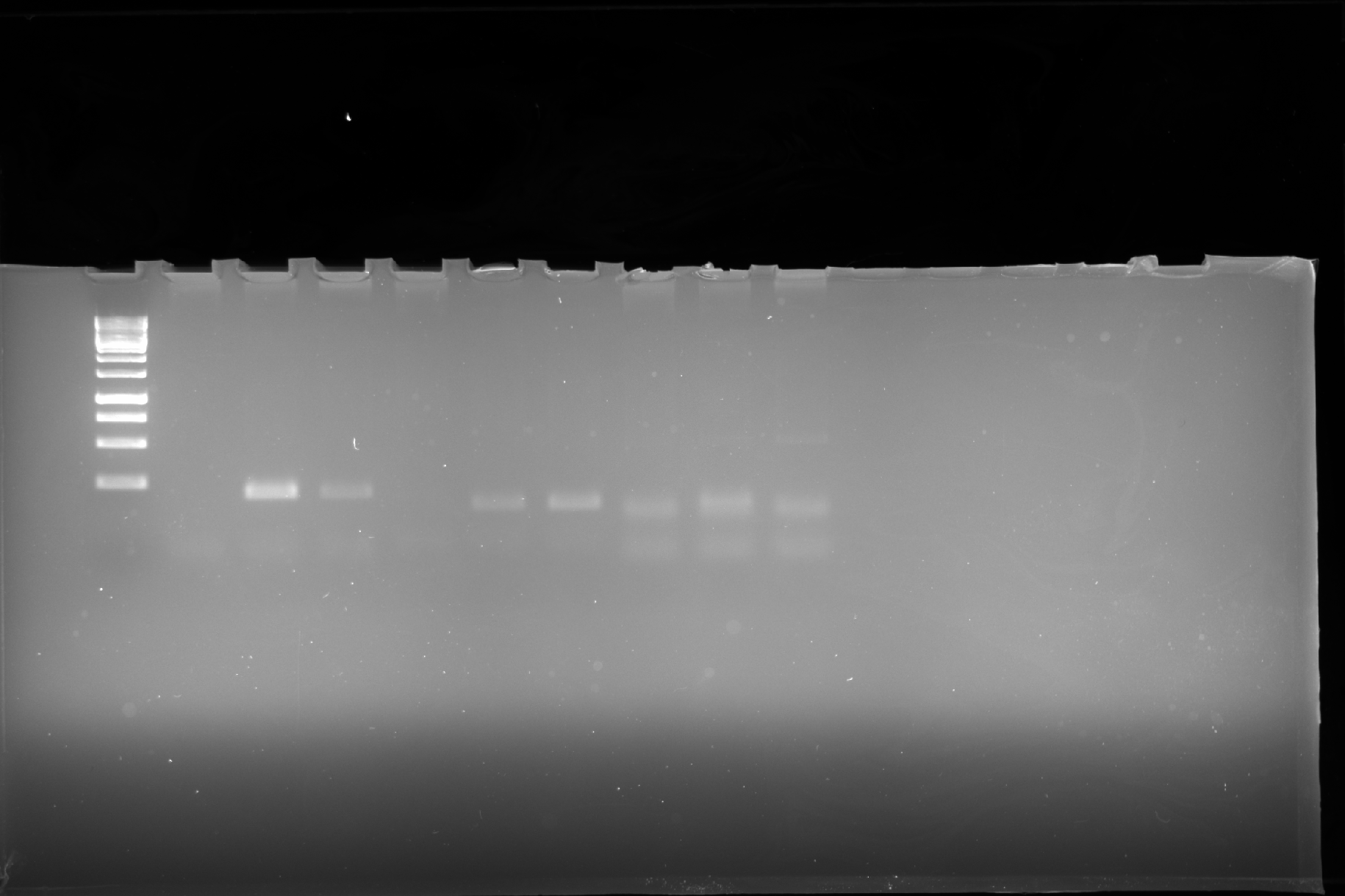

Supplement: Supplementary file 5 — Source data Fig. 1 [file 44319_2025_617_MOESM5_ESM.zip › Figure 1/Figure 1D/Used to quantify_Figure 1D_2.tif]

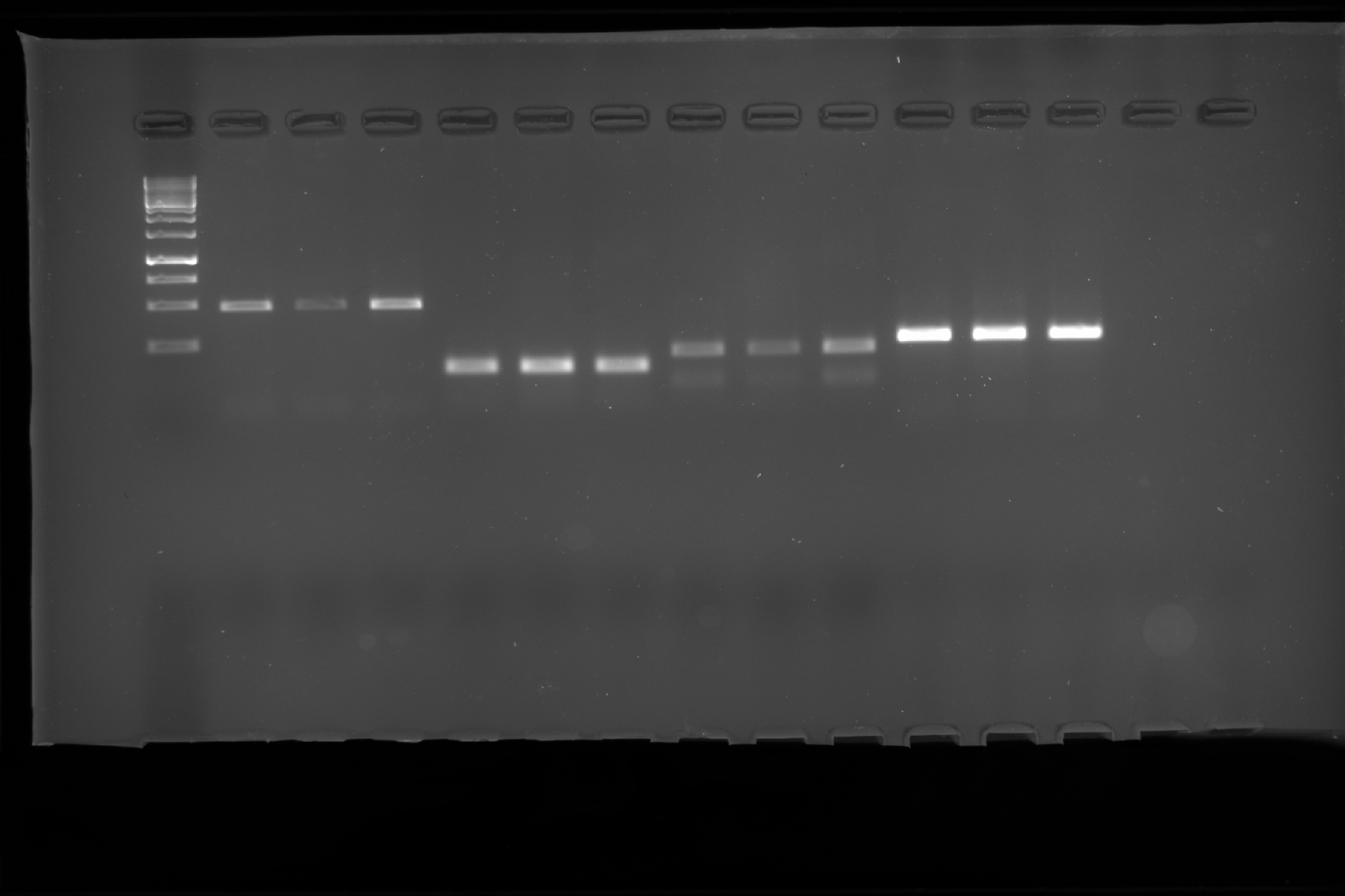

Supplement: Supplementary file 5 — Source data Fig. 1 [file 44319_2025_617_MOESM5_ESM.zip › Figure 1/Figure 1D/Used to quantify_Figure 1D_labelled.tif]

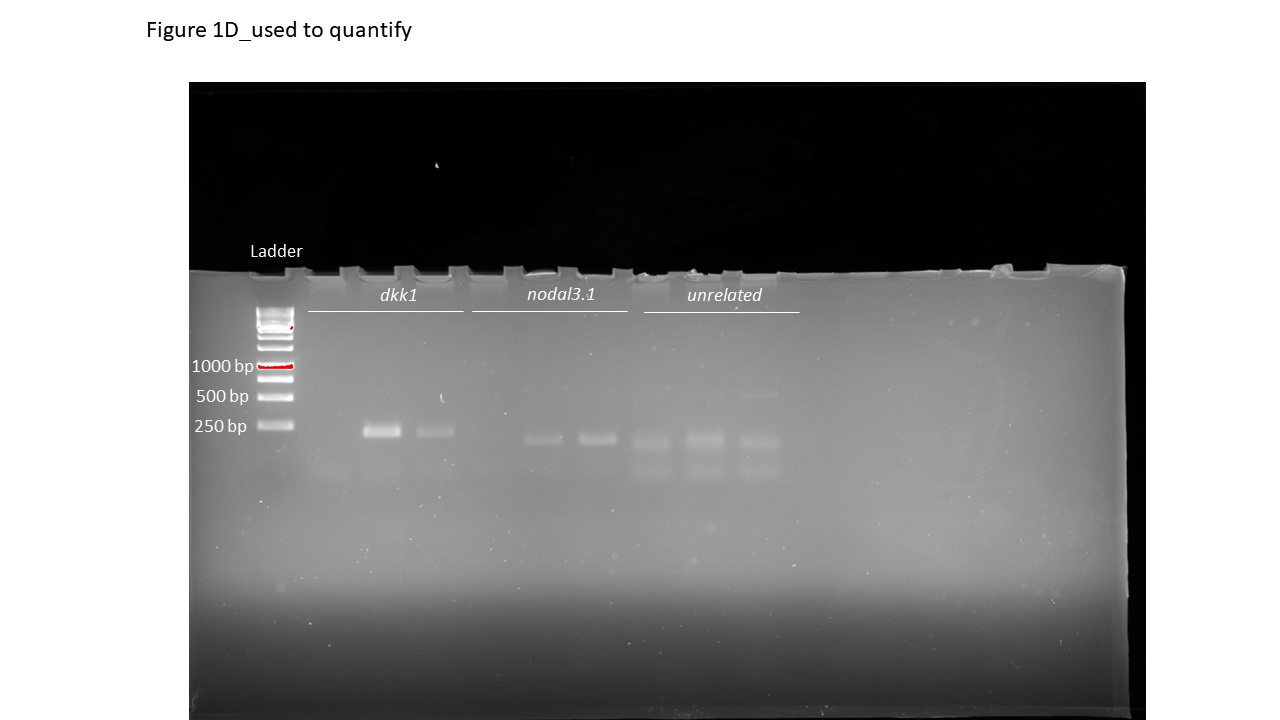

Supplement: Supplementary file 5 — Source data Fig. 1 [file 44319_2025_617_MOESM5_ESM.zip › Figure 1/Figure 1D/Used to quantify_Figure 1D_2_labelled.TIF]

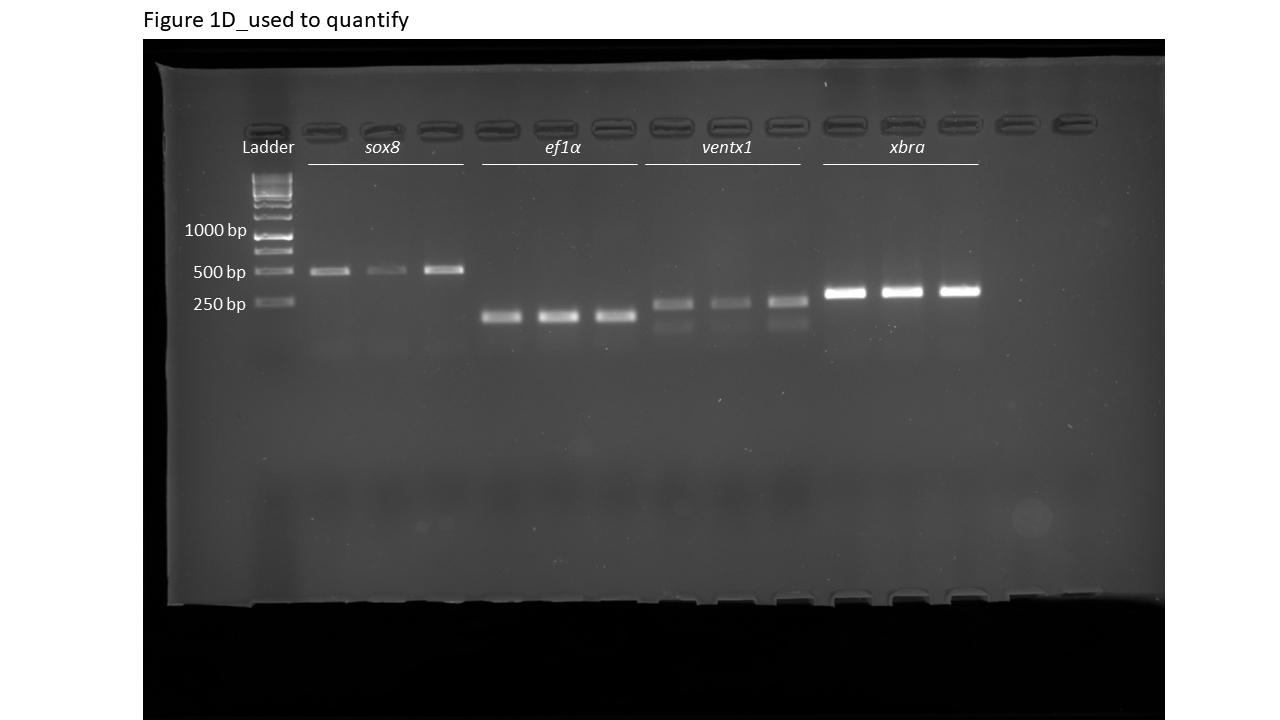

Supplement: Supplementary file 5 — Source data Fig. 1 [file 44319_2025_617_MOESM5_ESM.zip › Figure 1/Figure 1D/Used to quantify_Figure 1D_labbeled.TIF]

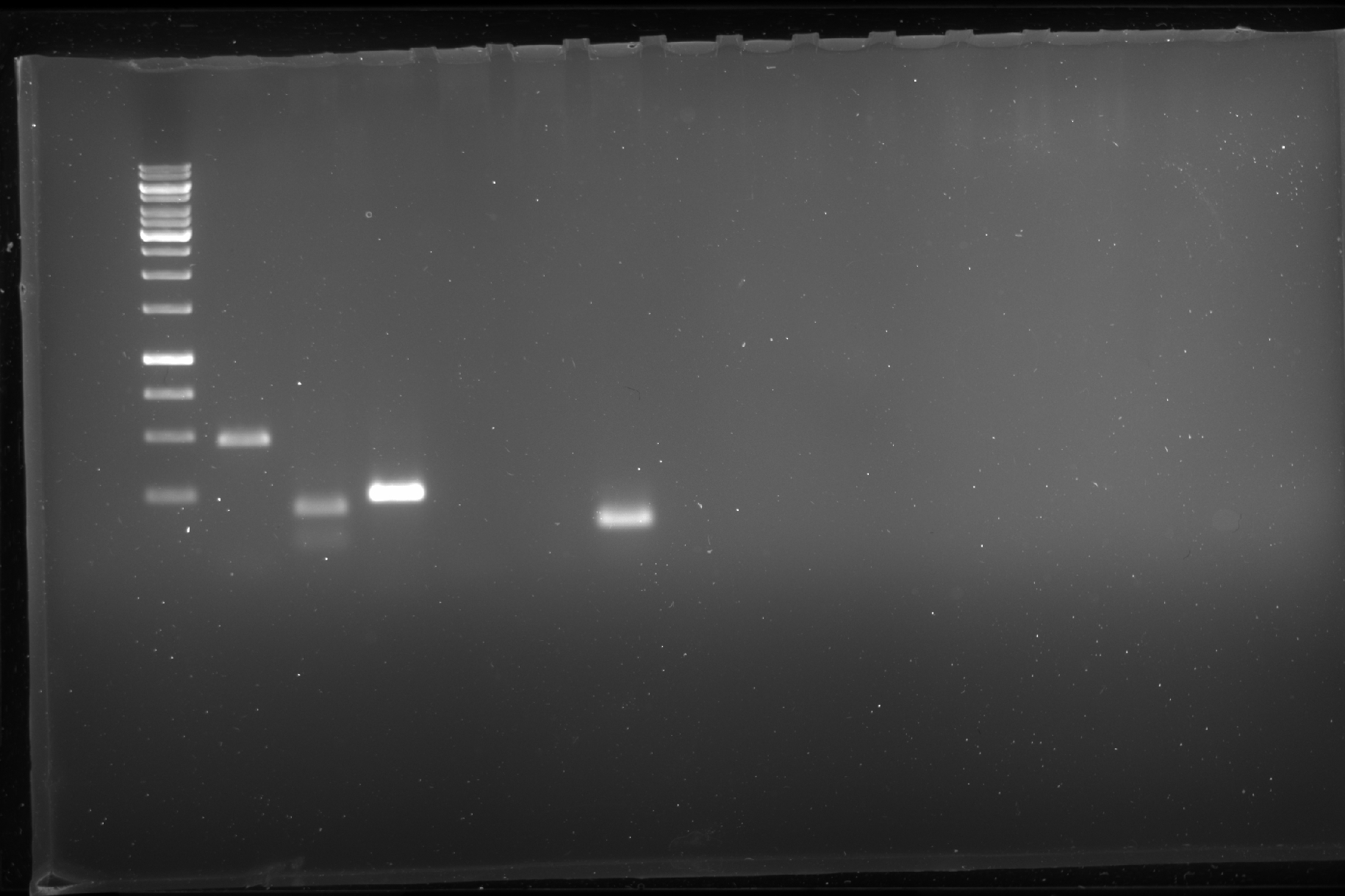

Supplement: Supplementary file 5 — Source data Fig. 1 [file 44319_2025_617_MOESM5_ESM.zip › Figure 1/Figure 1C/Figure 1C_original.tif]

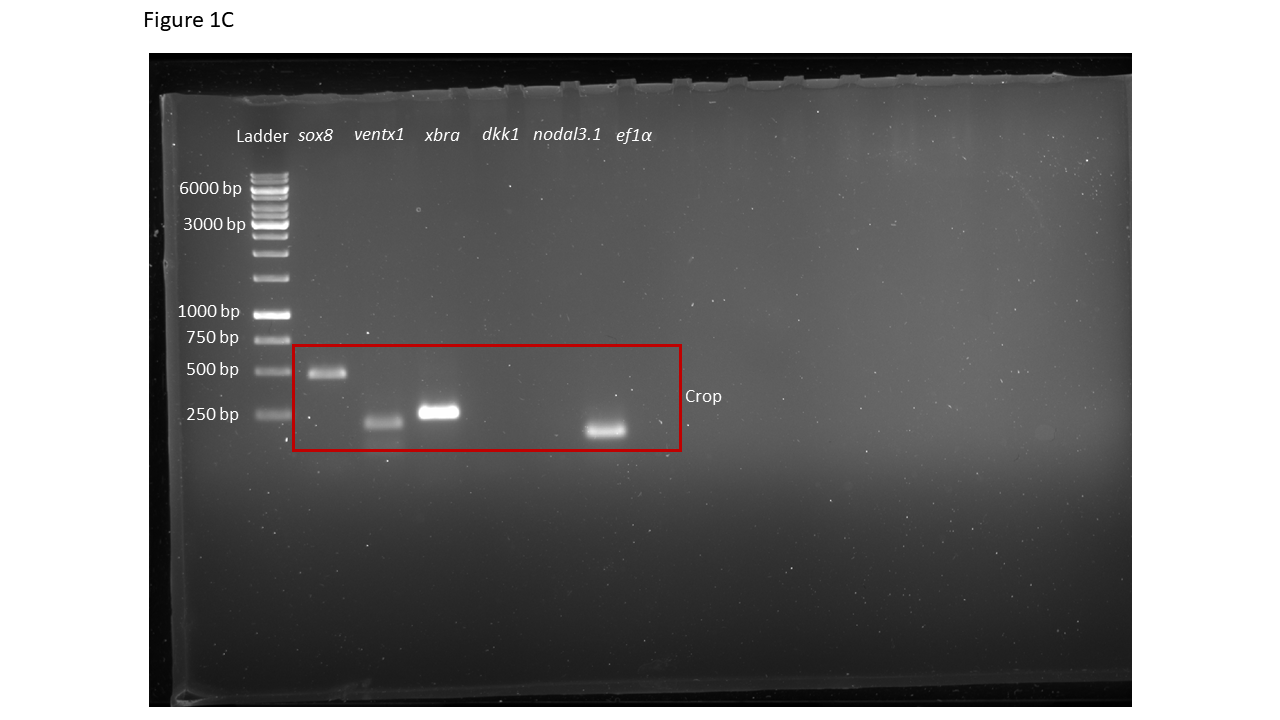

Supplement: Supplementary file 5 — Source data Fig. 1 [file 44319_2025_617_MOESM5_ESM.zip › Figure 1/Figure 1C/Figure 1c_labelled.TIF]

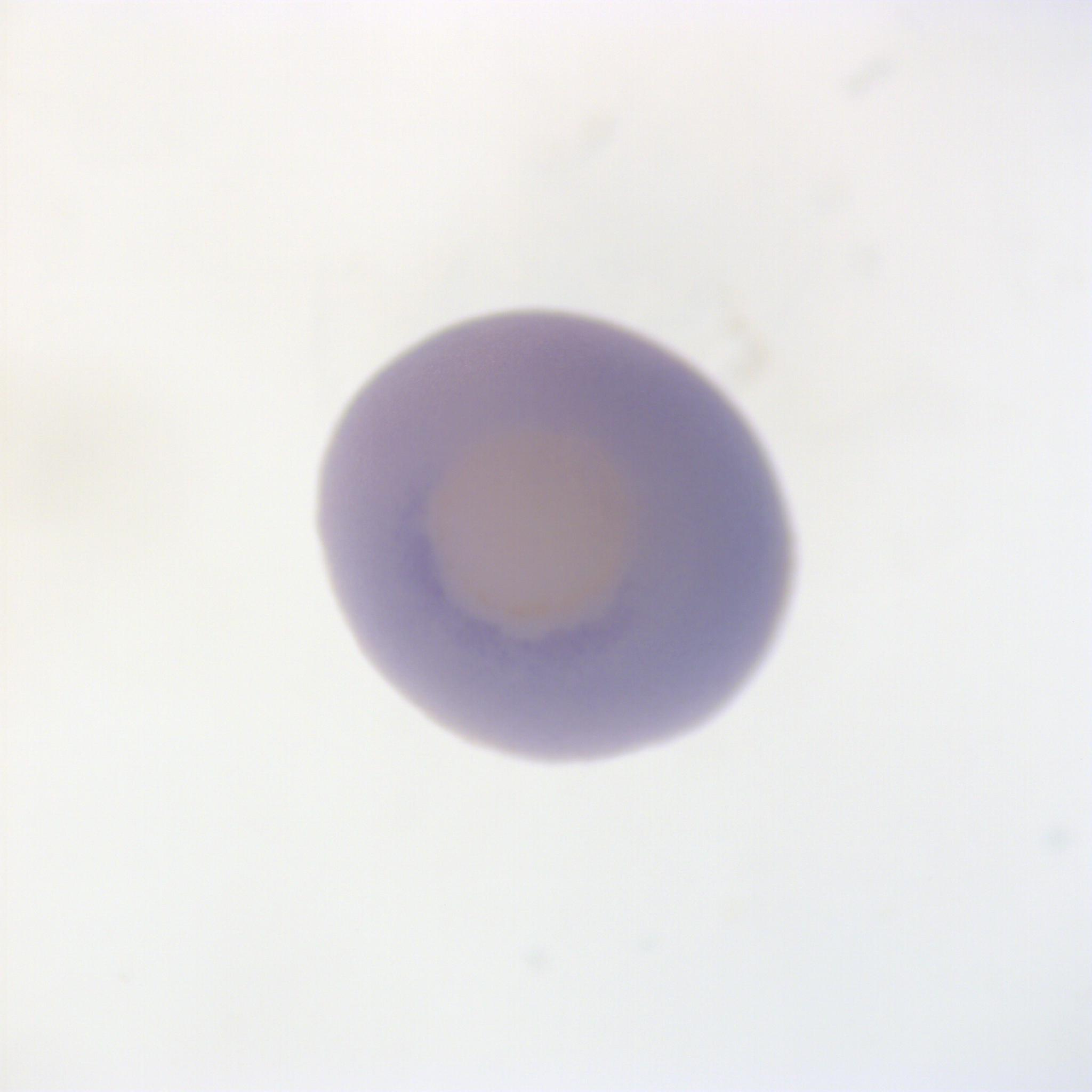

Supplement: Supplementary file 5 — Source data Fig. 1 [file 44319_2025_617_MOESM5_ESM.zip › Figure 1/Figure 1F/Sox8 ISH/3.1.tif]

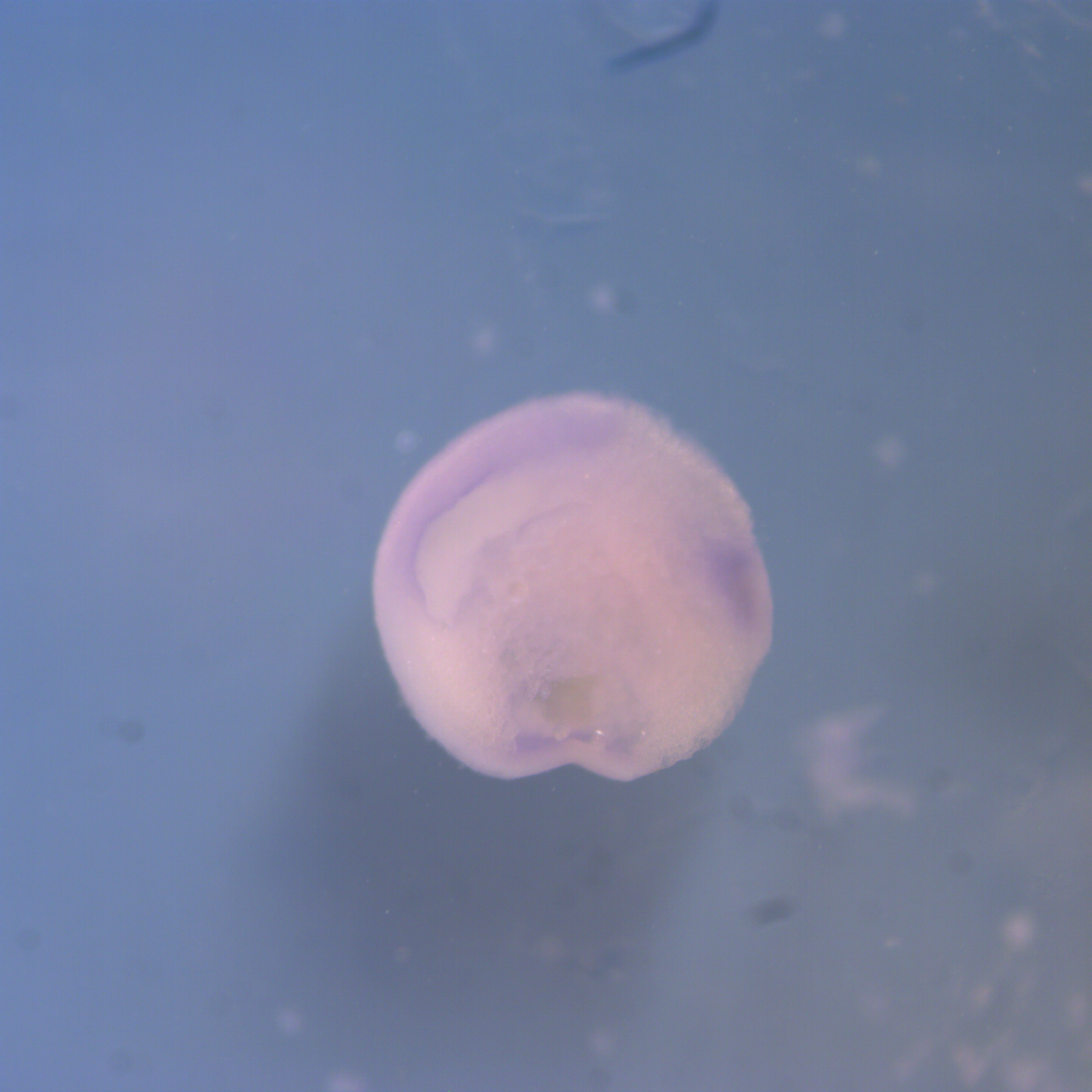

Supplement: Supplementary file 5 — Source data Fig. 1 [file 44319_2025_617_MOESM5_ESM.zip › Figure 1/Figure 1F/Sox8 ISH/23.tif]

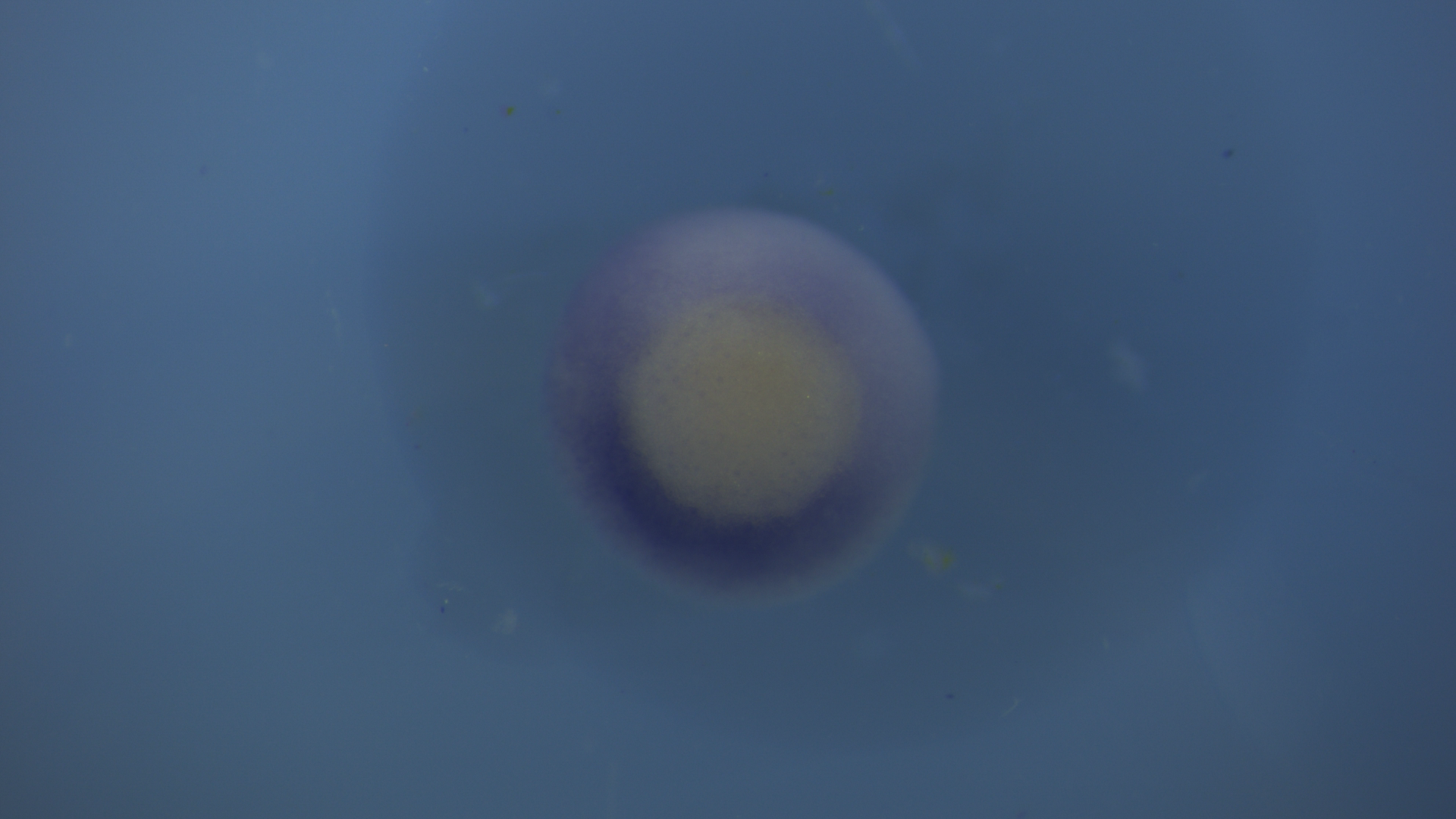

Supplement: Supplementary file 5 — Source data Fig. 1 [file 44319_2025_617_MOESM5_ESM.zip › Figure 1/Figure 1F/Ventx ISH/6_ventx1231003.tif]

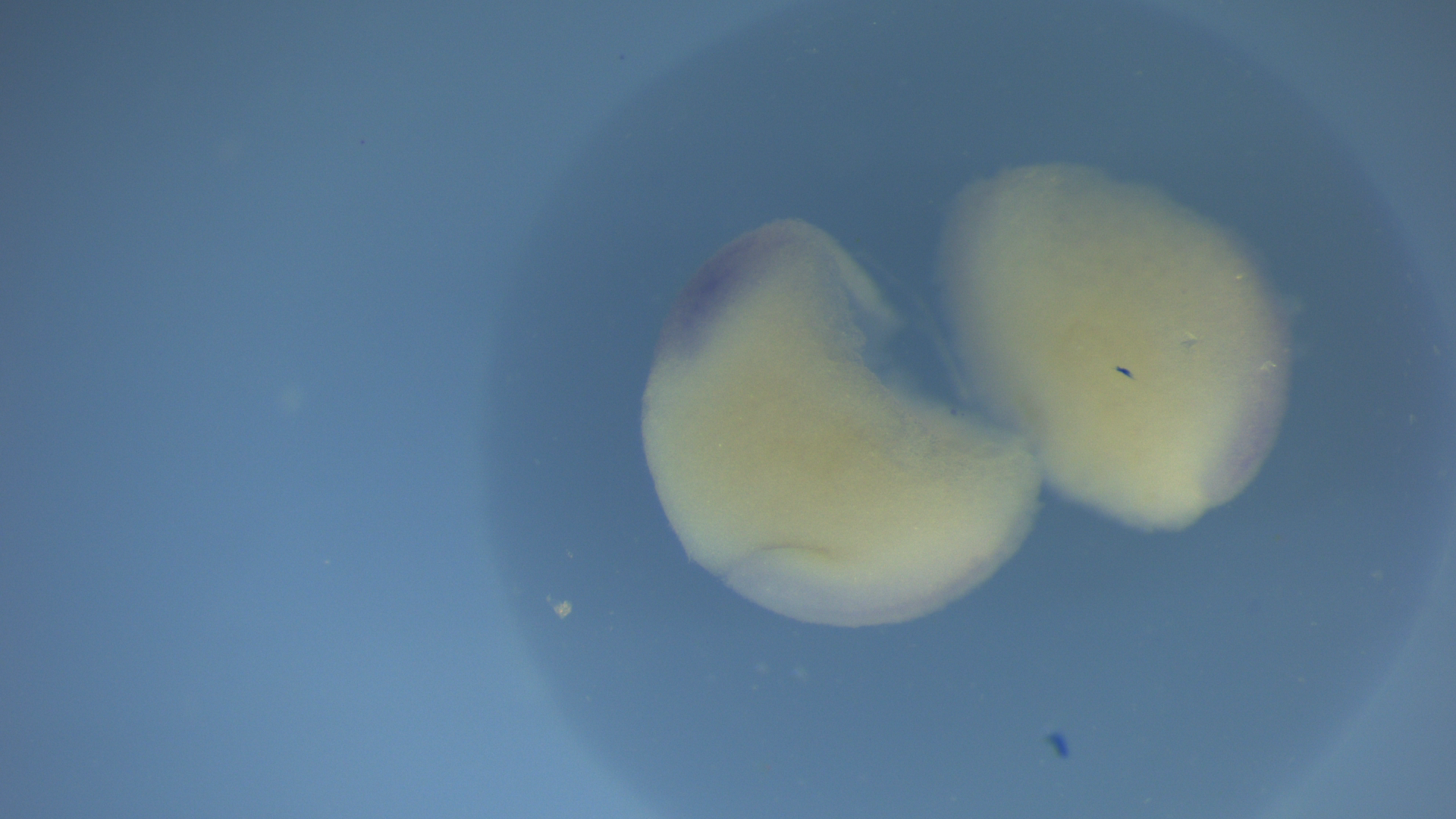

Supplement: Supplementary file 5 — Source data Fig. 1 [file 44319_2025_617_MOESM5_ESM.zip › Figure 1/Figure 1F/Ventx ISH/5_ventx12310035.tif]

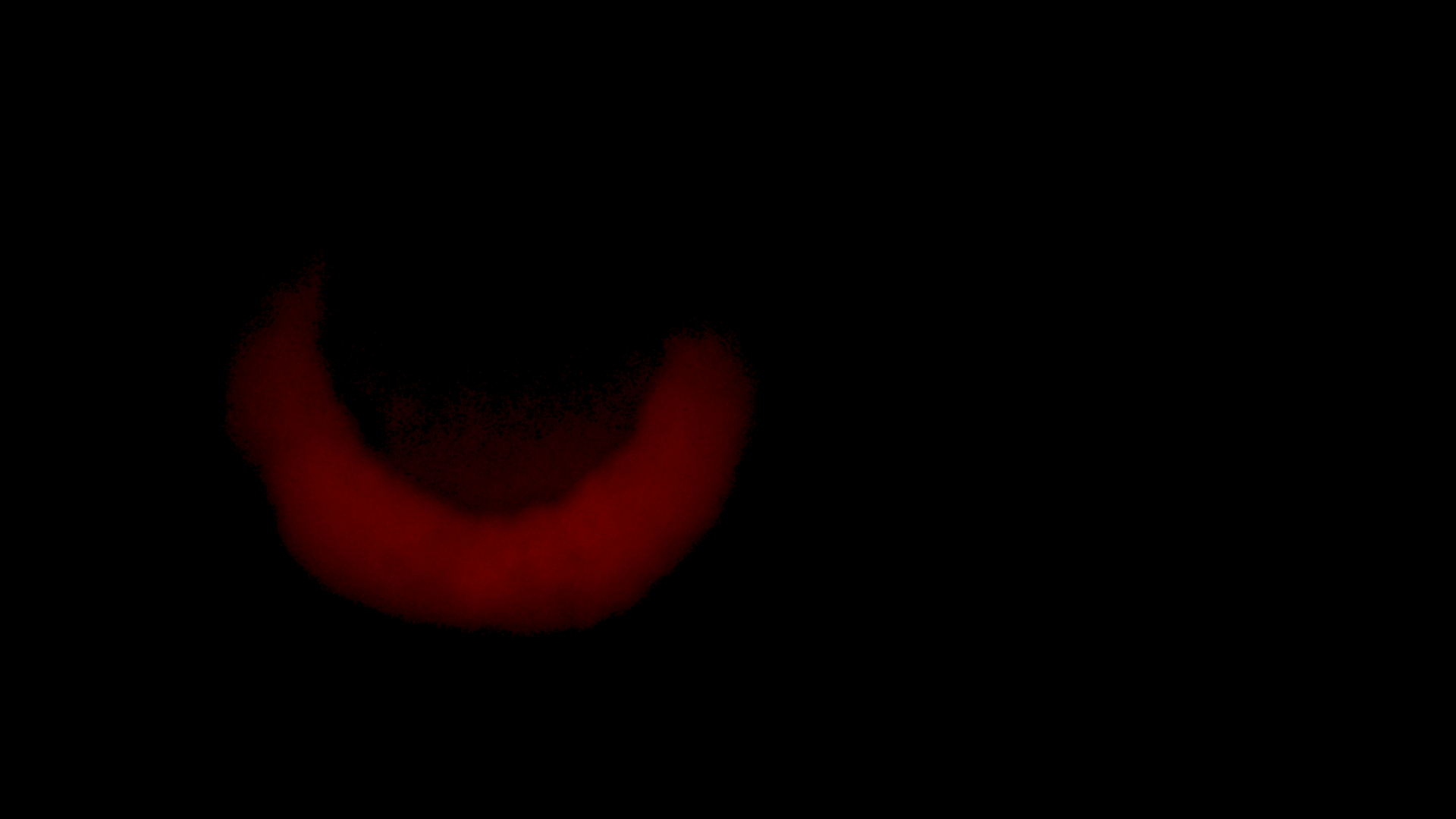

Supplement: Supplementary file 6 — Source data Fig. 2 [file 44319_2025_617_MOESM6_ESM.zip › Figure 2/Figure 2C/sox8 CRISPR.tif]

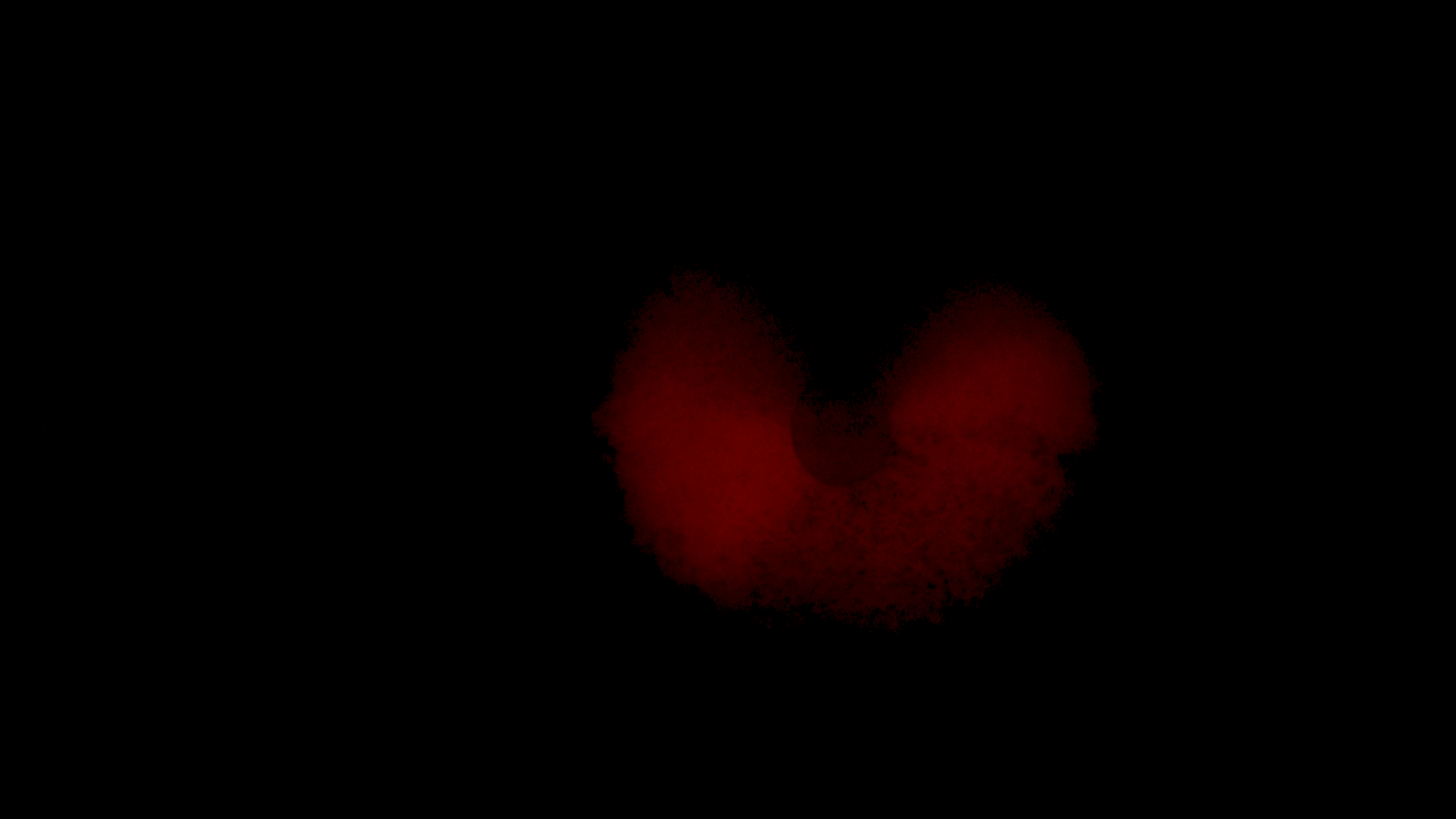

Supplement: Supplementary file 6 — Source data Fig. 2 [file 44319_2025_617_MOESM6_ESM.zip › Figure 2/Figure 2C/Control.tif]

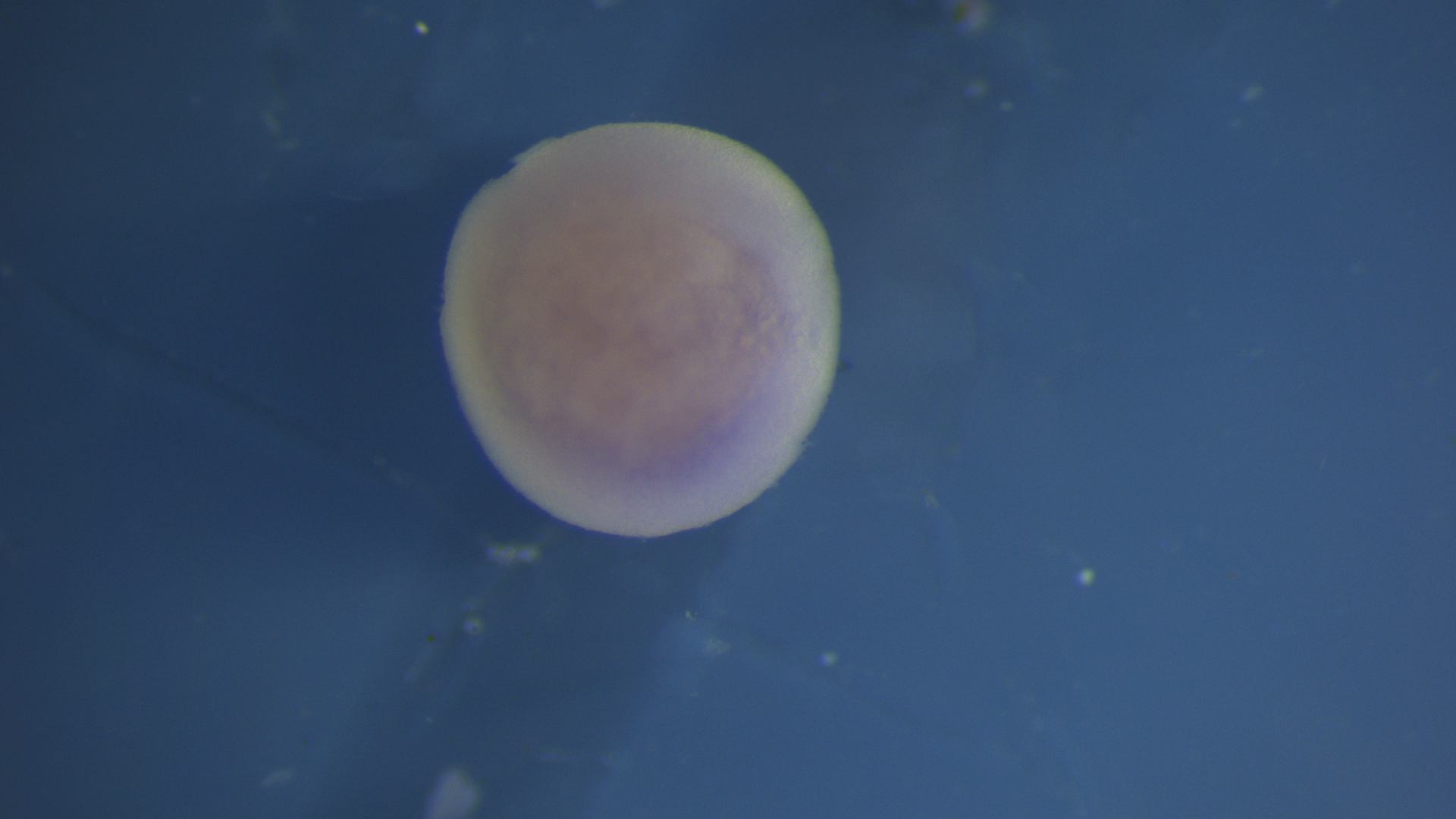

Supplement: Supplementary file 6 — Source data Fig. 2 [file 44319_2025_617_MOESM6_ESM.zip › Figure 2/Figure 2D/sox8 CRISPR.tif]

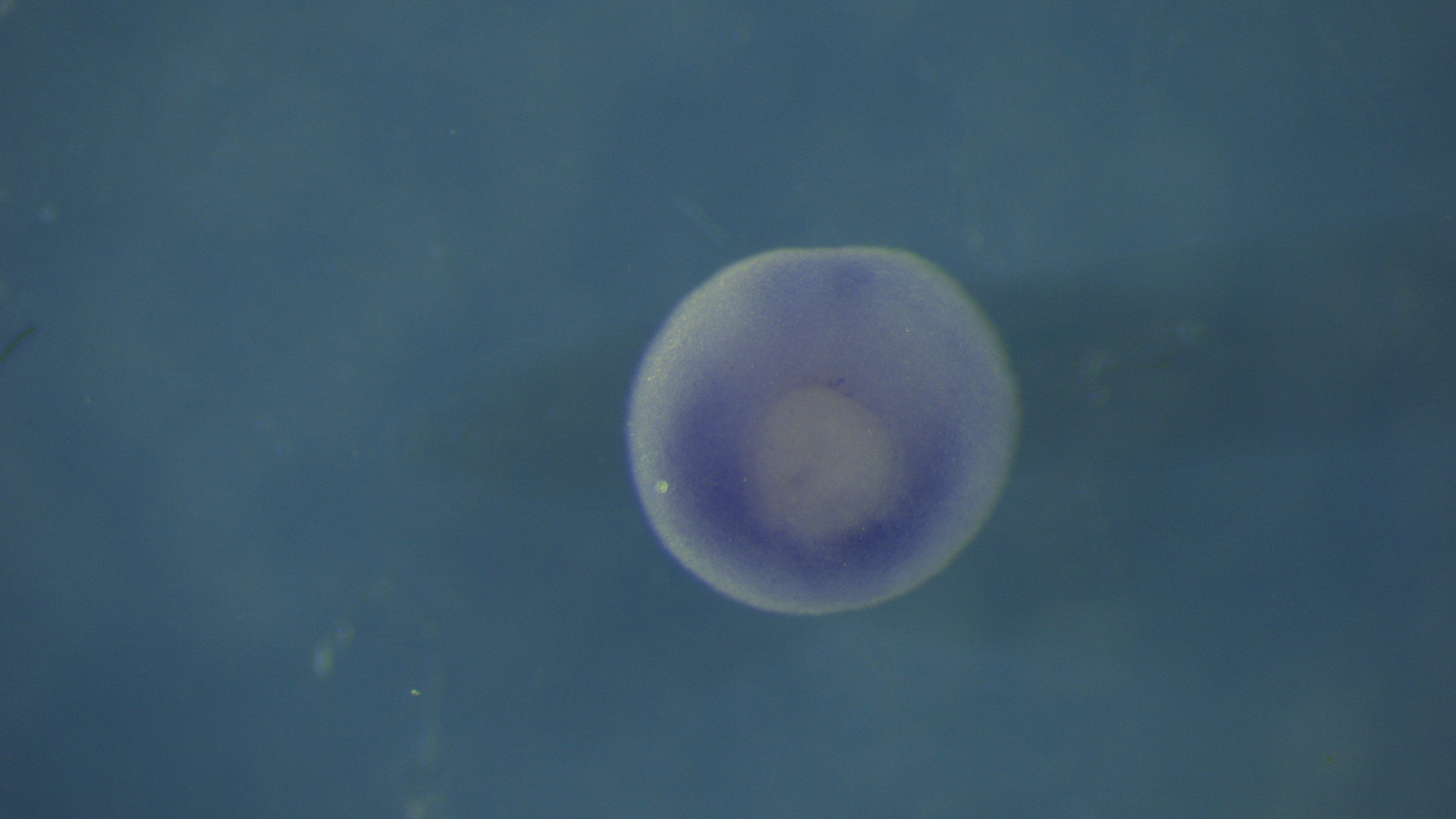

Supplement: Supplementary file 6 — Source data Fig. 2 [file 44319_2025_617_MOESM6_ESM.zip › Figure 2/Figure 2D/Control.tif]

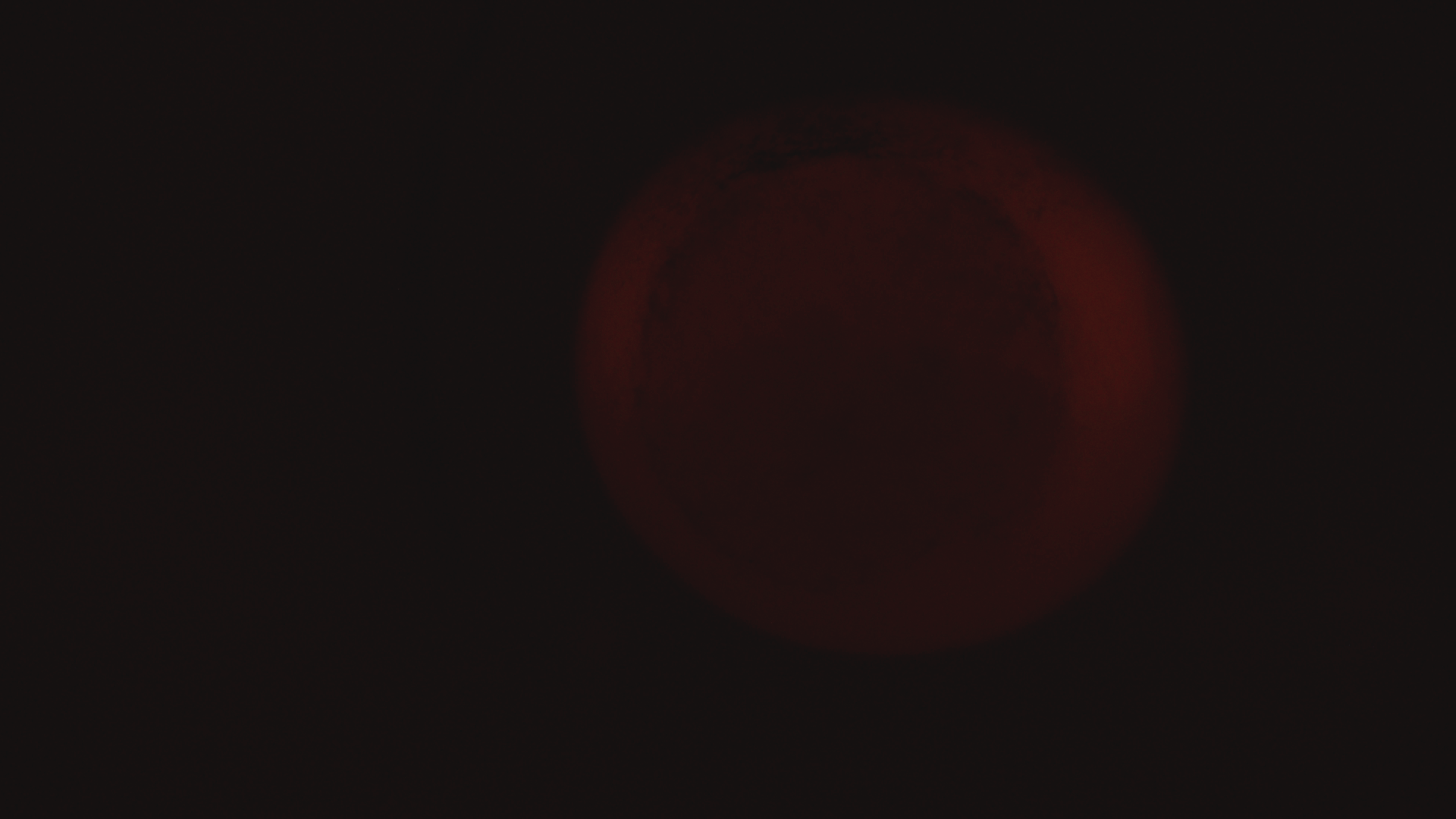

Supplement: Supplementary file 6 — Source data Fig. 2 [file 44319_2025_617_MOESM6_ESM.zip › Figure 2/Figure 2E/Cas711_160pgguides_30_102311011.tif]

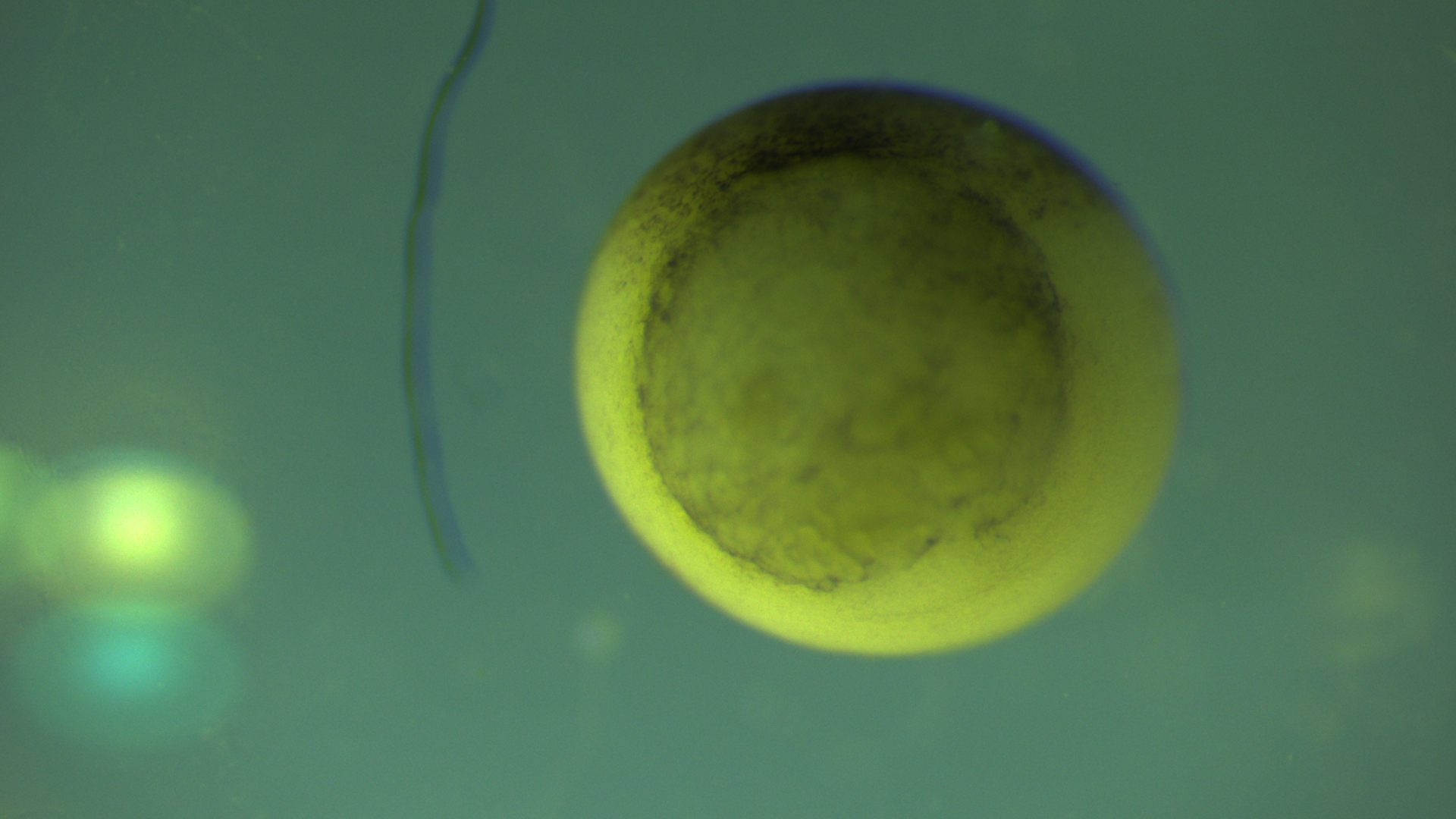

Supplement: Supplementary file 6 — Source data Fig. 2 [file 44319_2025_617_MOESM6_ESM.zip › Figure 2/Figure 2E/Cas711_160pgguides_30_10231101.tif]

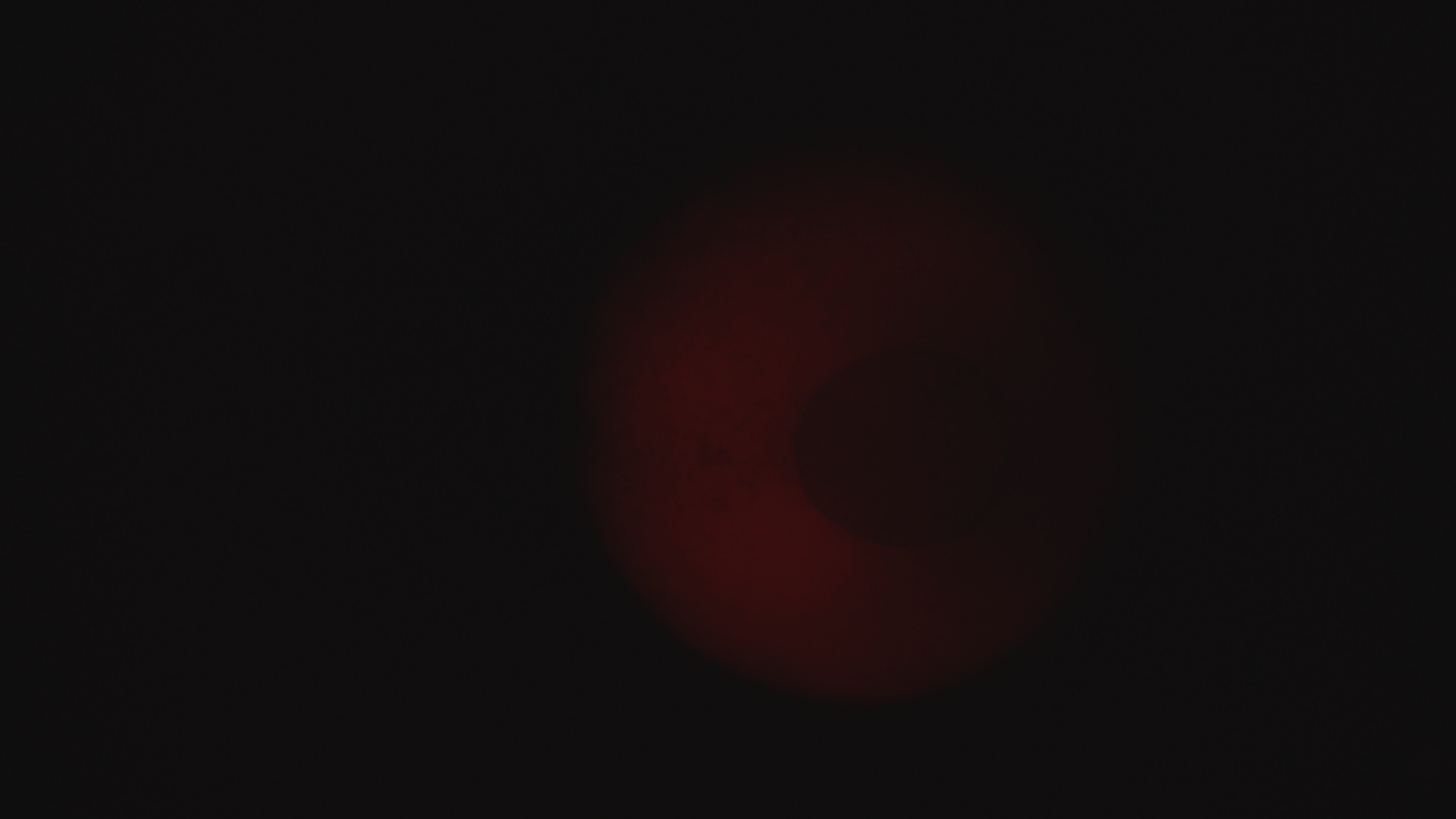

Supplement: Supplementary file 6 — Source data Fig. 2 [file 44319_2025_617_MOESM6_ESM.zip › Figure 2/Figure 2E/160pg100pgsox_171023101827.tif]

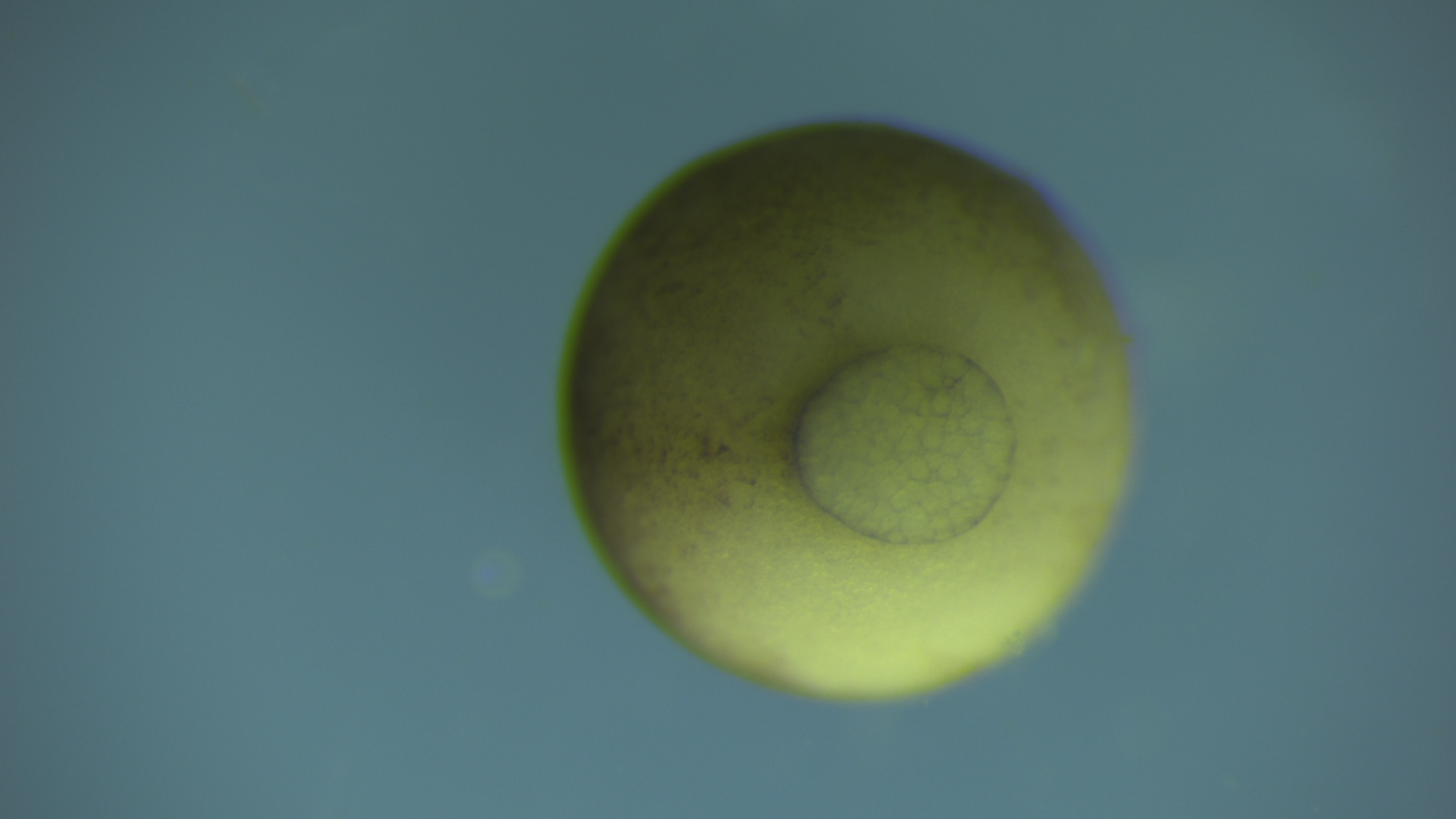

Supplement: Supplementary file 6 — Source data Fig. 2 [file 44319_2025_617_MOESM6_ESM.zip › Figure 2/Figure 2E/160pg100pgsox_171023101826.tif]

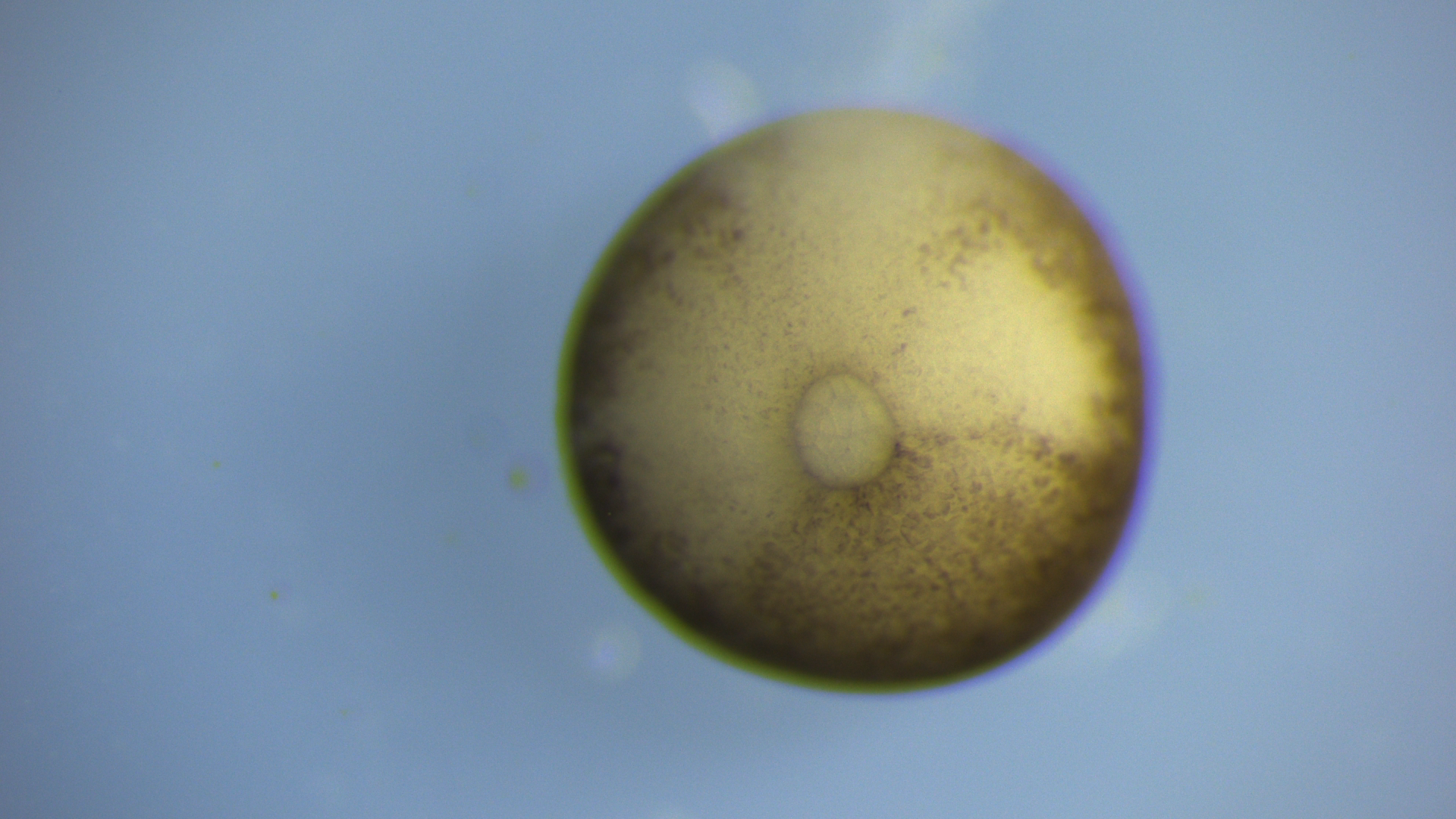

Supplement: Supplementary file 6 — Source data Fig. 2 [file 44319_2025_617_MOESM6_ESM.zip › Figure 2/Figure 2E/Cas711_23_11231124.tif]

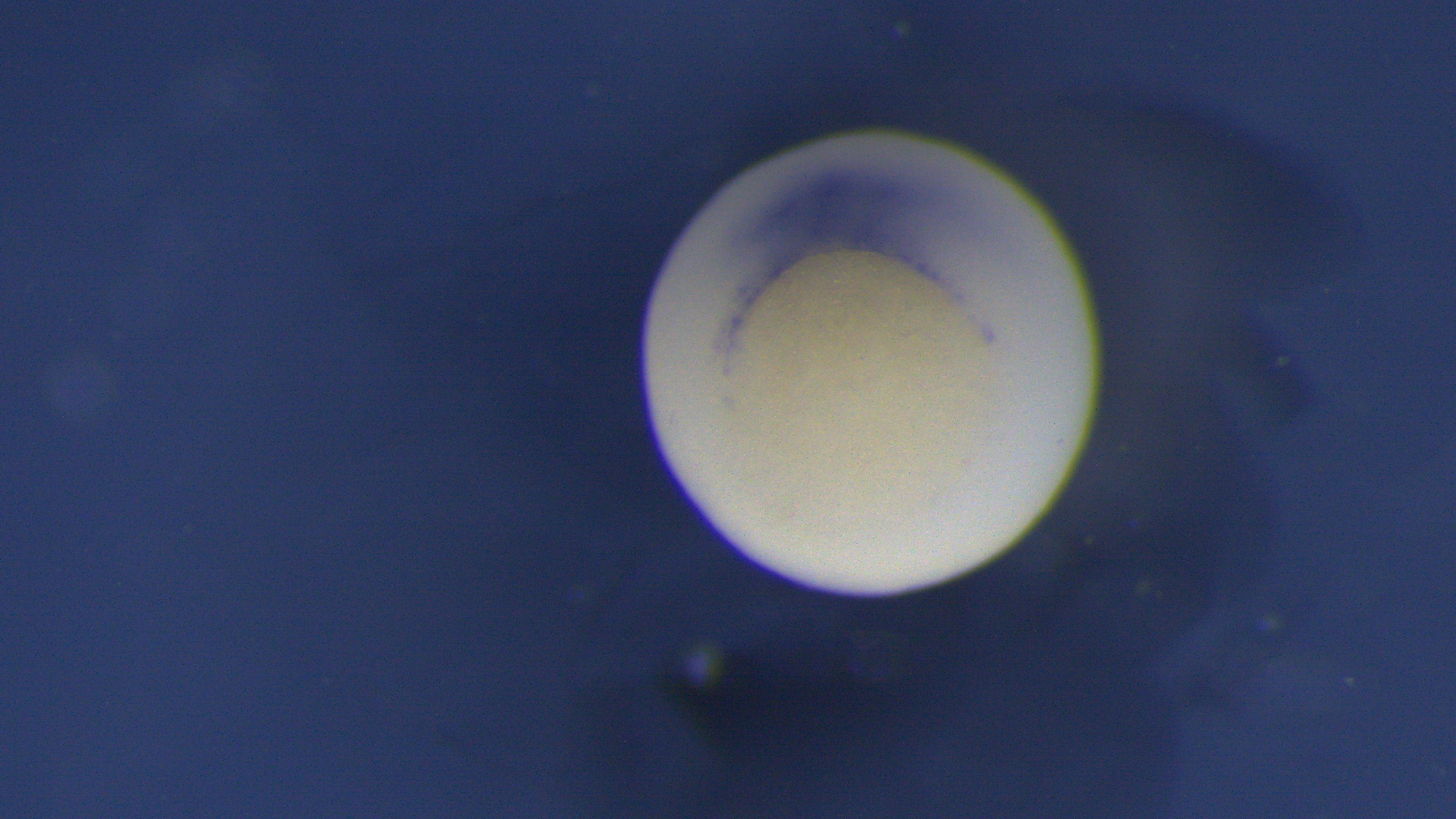

Supplement: Supplementary file 7 — Source data Fig. 3 [file 44319_2025_617_MOESM7_ESM.zip › Figure 3/Figure 3E,F,G/sox CRISPR Chrd.jpg]

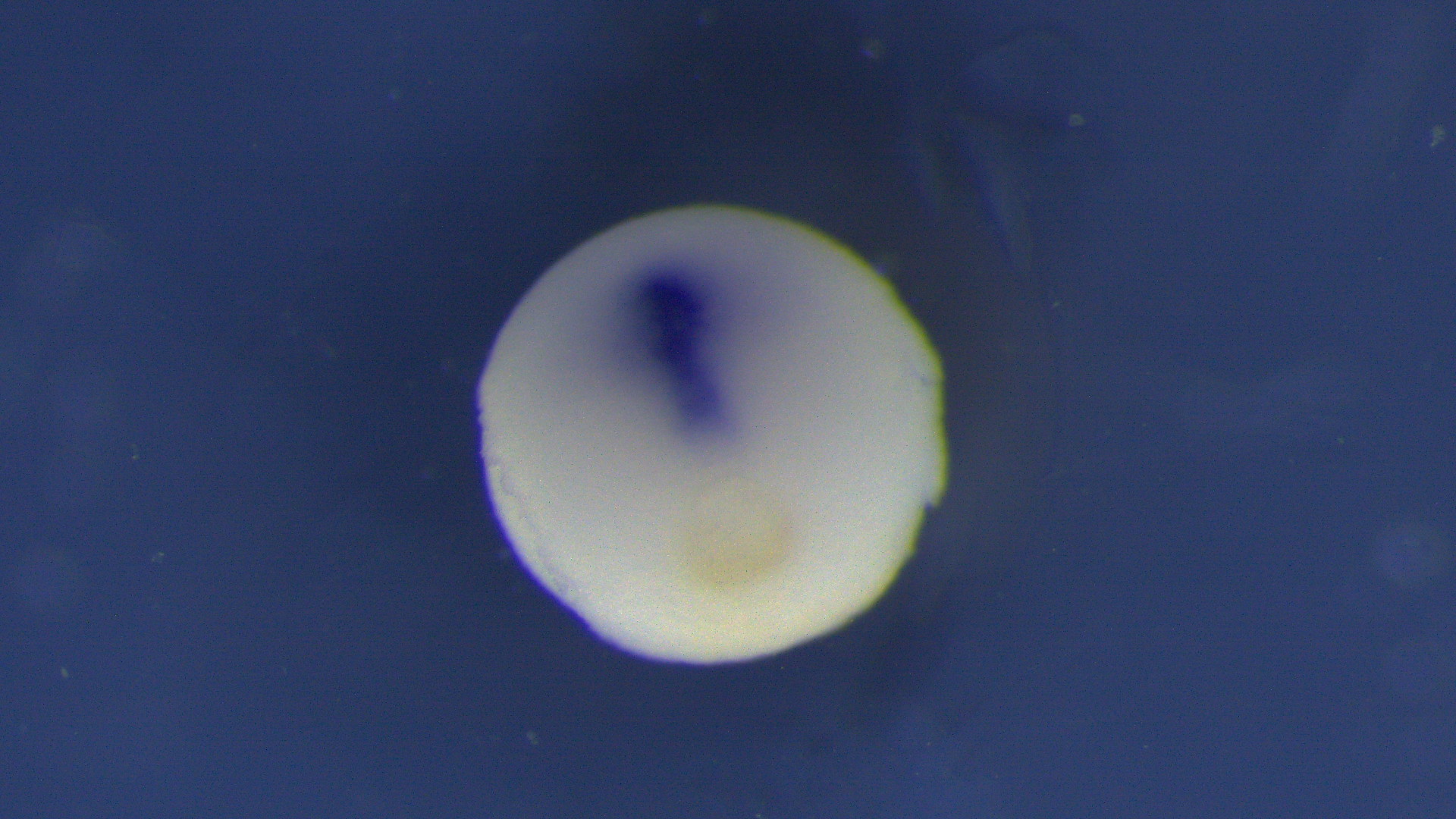

Supplement: Supplementary file 7 — Source data Fig. 3 [file 44319_2025_617_MOESM7_ESM.zip › Figure 3/Figure 3E,F,G/Control Chrd.jpg]

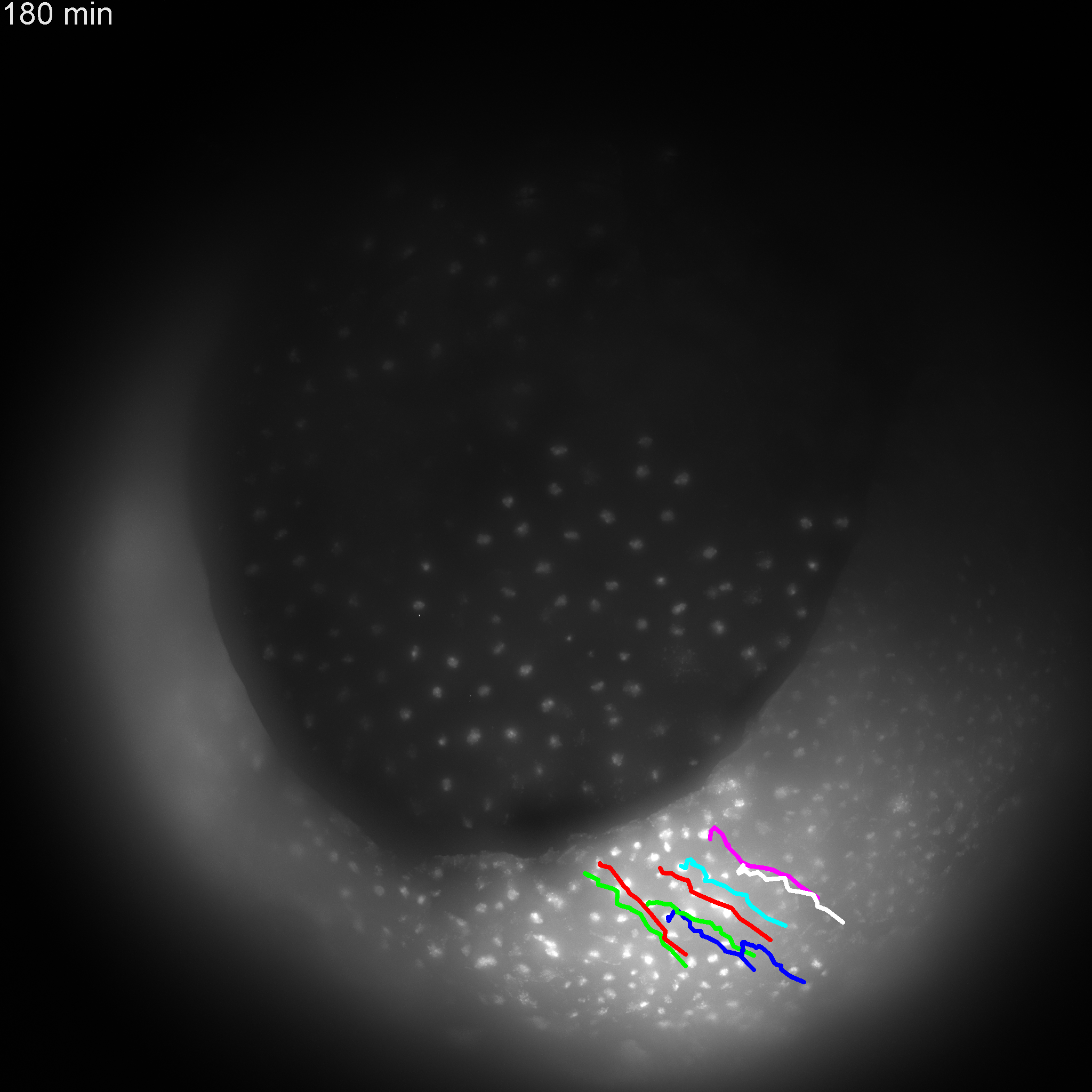

Supplement: Supplementary file 7 — Source data Fig. 3 [file 44319_2025_617_MOESM7_ESM.zip › Figure 3/Figure 3B/Control_overlay dots and lines.tif]

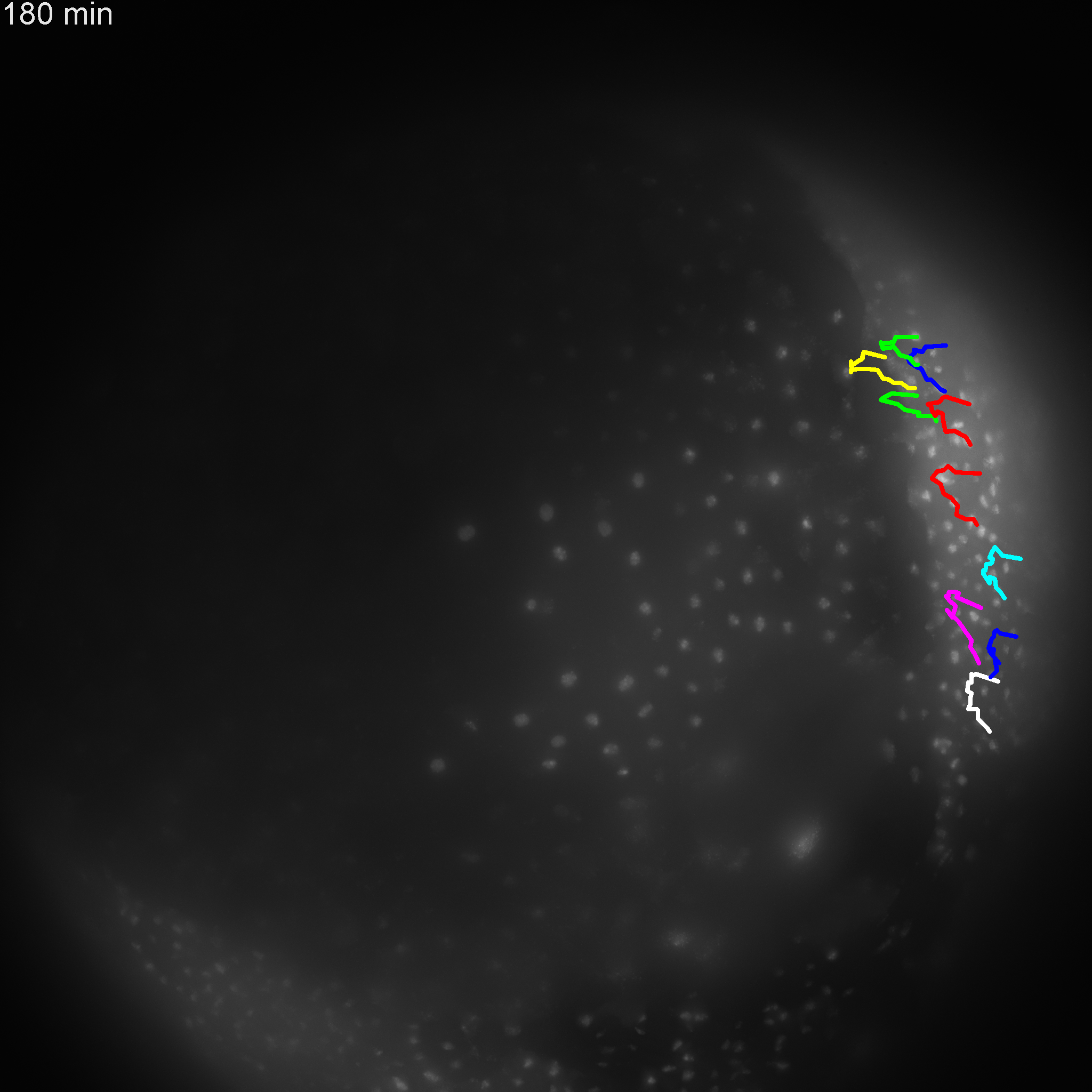

Supplement: Supplementary file 7 — Source data Fig. 3 [file 44319_2025_617_MOESM7_ESM.zip › Figure 3/Figure 3B/sox8 CRISPR_overlay dots and lines.tif]

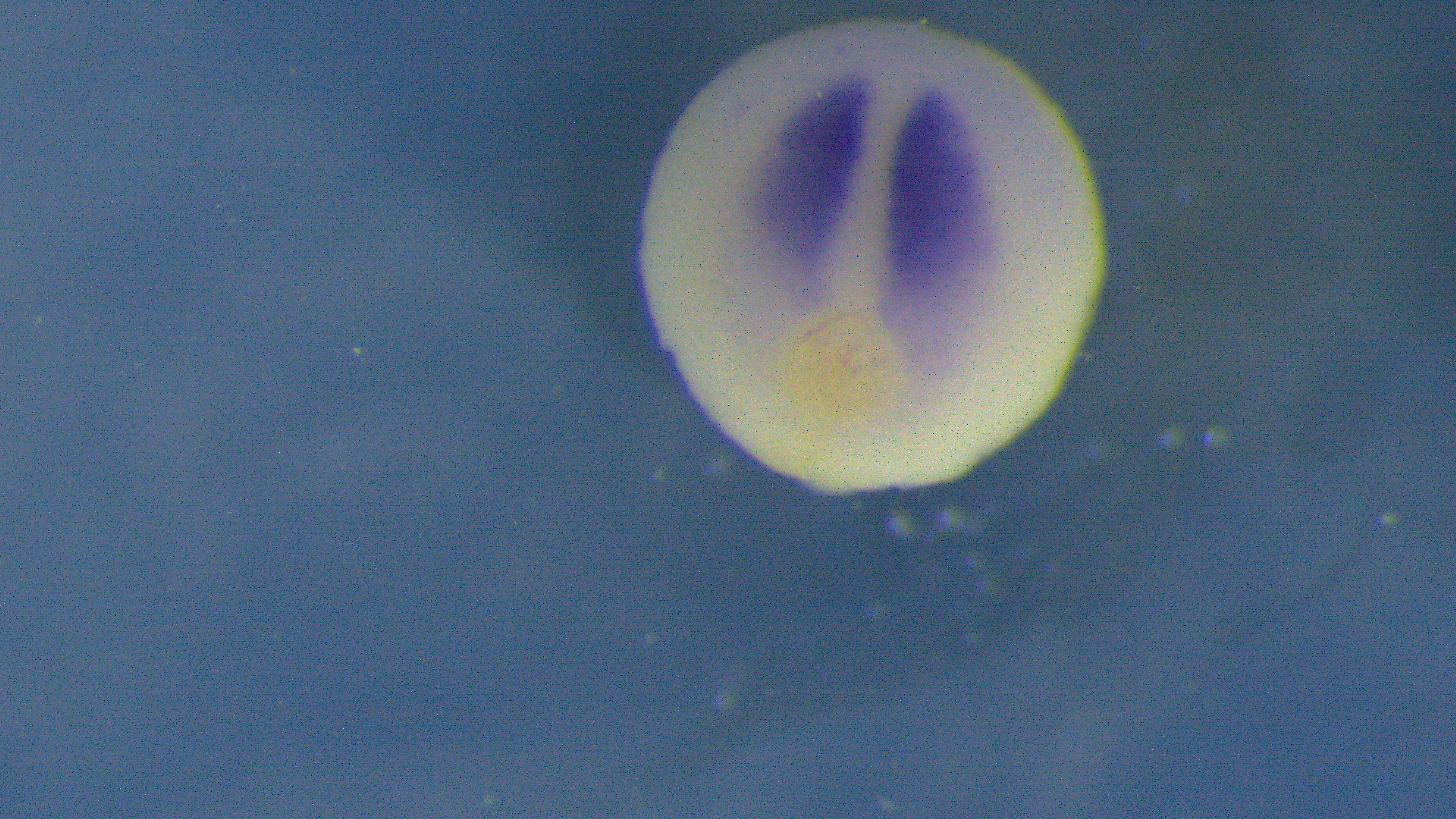

Supplement: Supplementary file 7 — Source data Fig. 3 [file 44319_2025_617_MOESM7_ESM.zip › Figure 3/Figure 3K,L,M/Control myoD.jpg]

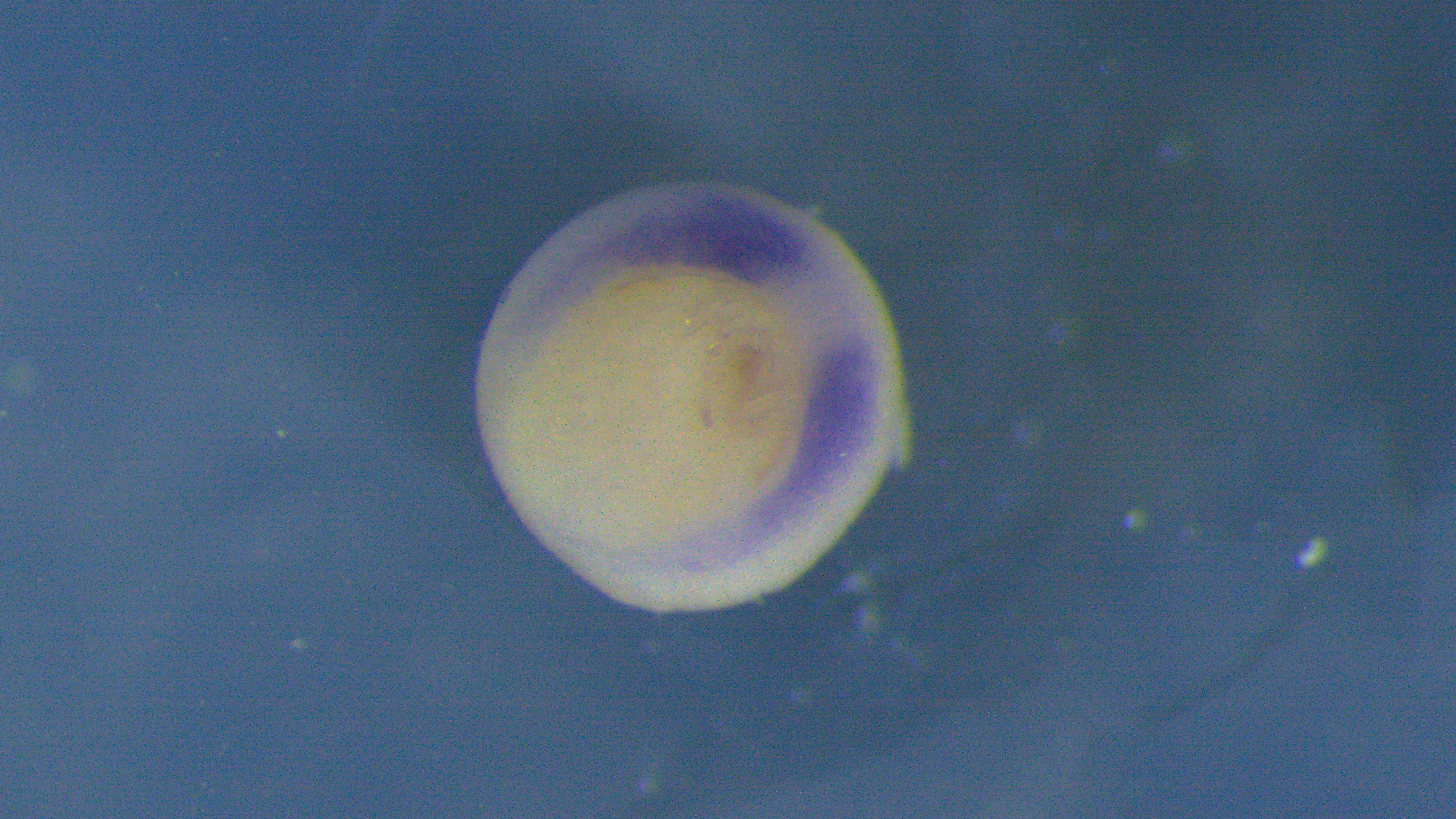

Supplement: Supplementary file 7 — Source data Fig. 3 [file 44319_2025_617_MOESM7_ESM.zip › Figure 3/Figure 3K,L,M/sox8 CRISPR myoD.jpg]

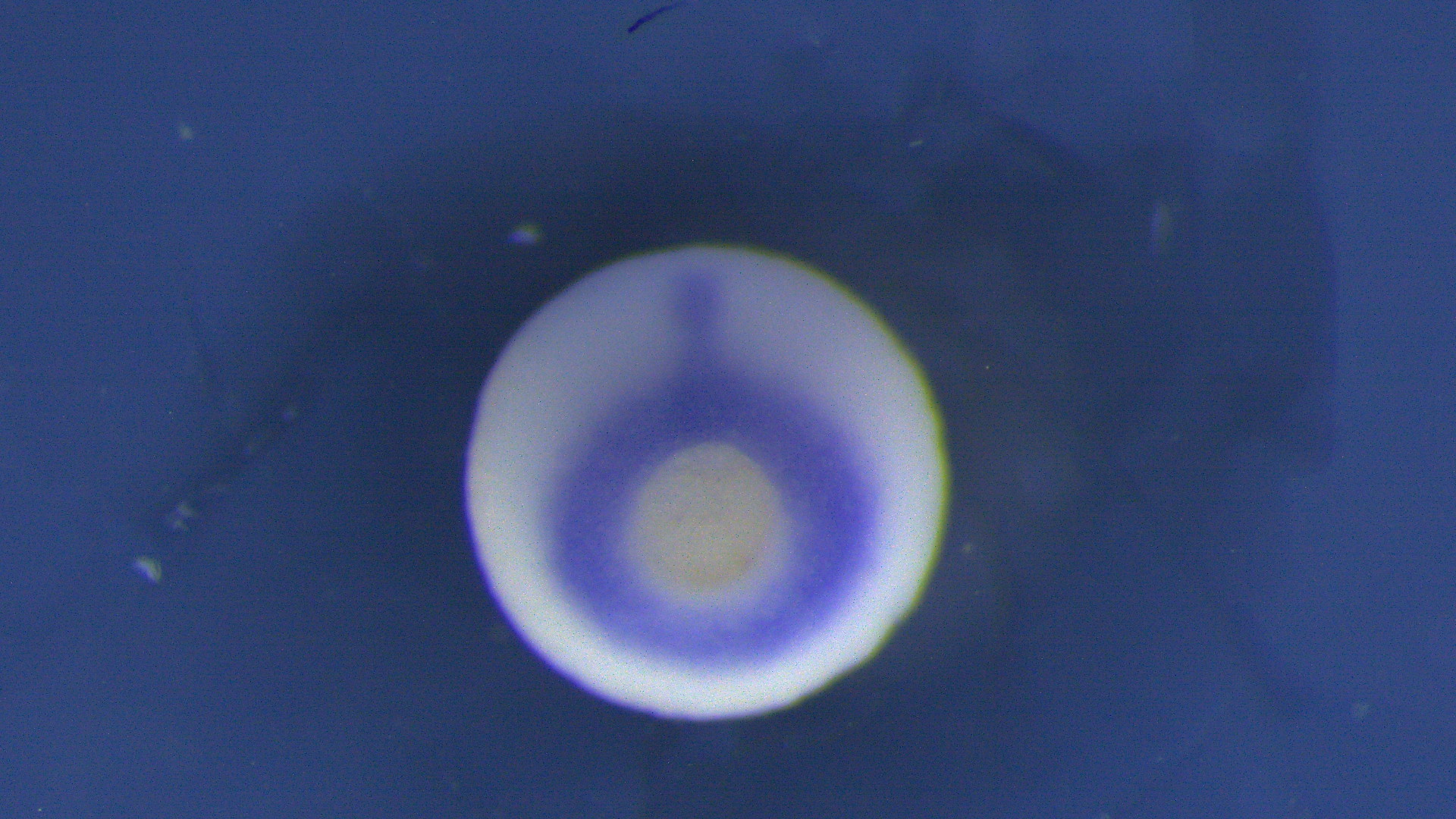

Supplement: Supplementary file 7 — Source data Fig. 3 [file 44319_2025_617_MOESM7_ESM.zip › Figure 3/Figure 3H,I,J/Control Xbra.jpg]

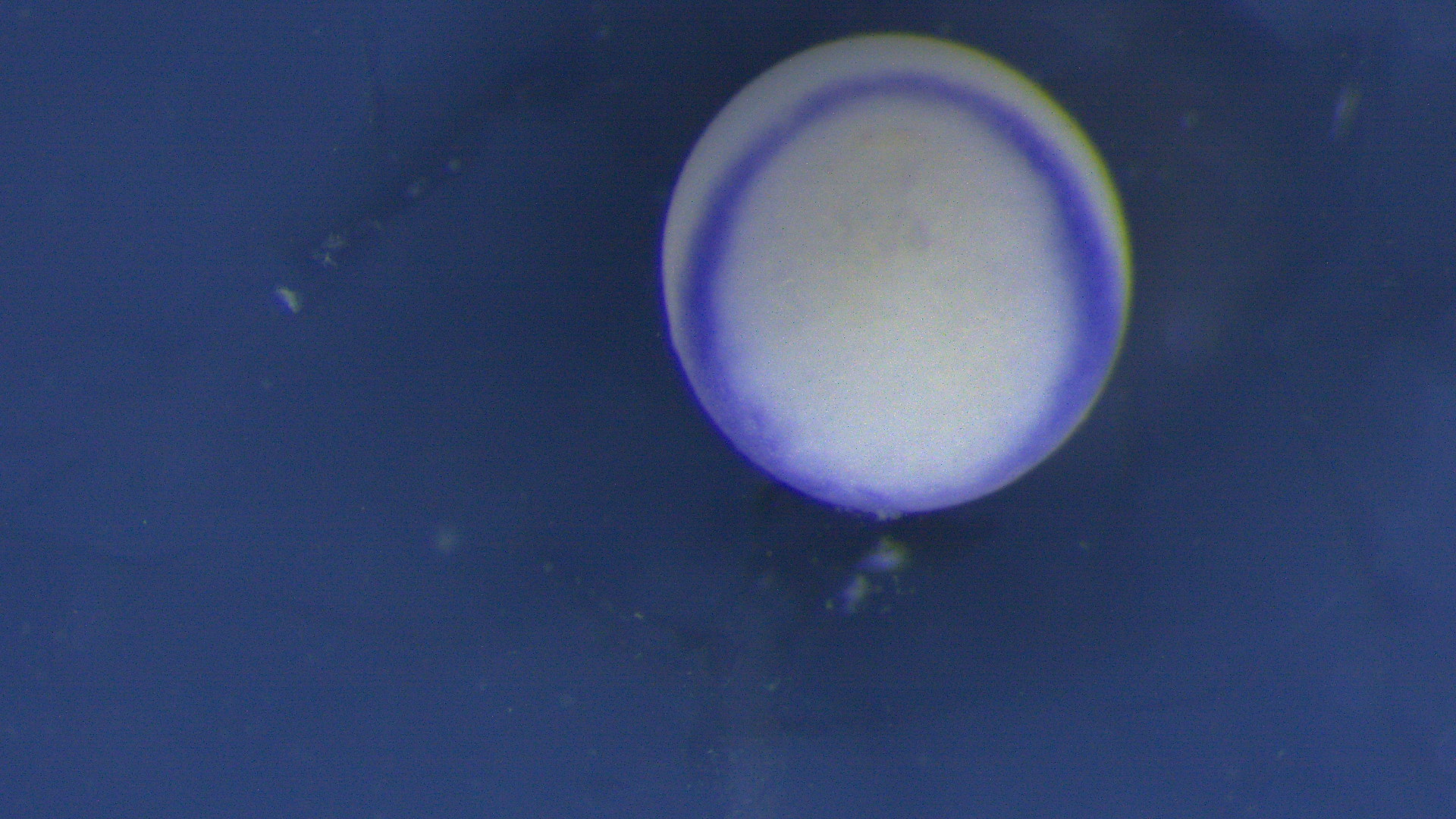

Supplement: Supplementary file 7 — Source data Fig. 3 [file 44319_2025_617_MOESM7_ESM.zip › Figure 3/Figure 3H,I,J/sox8 CRISPR Xbra.jpg]

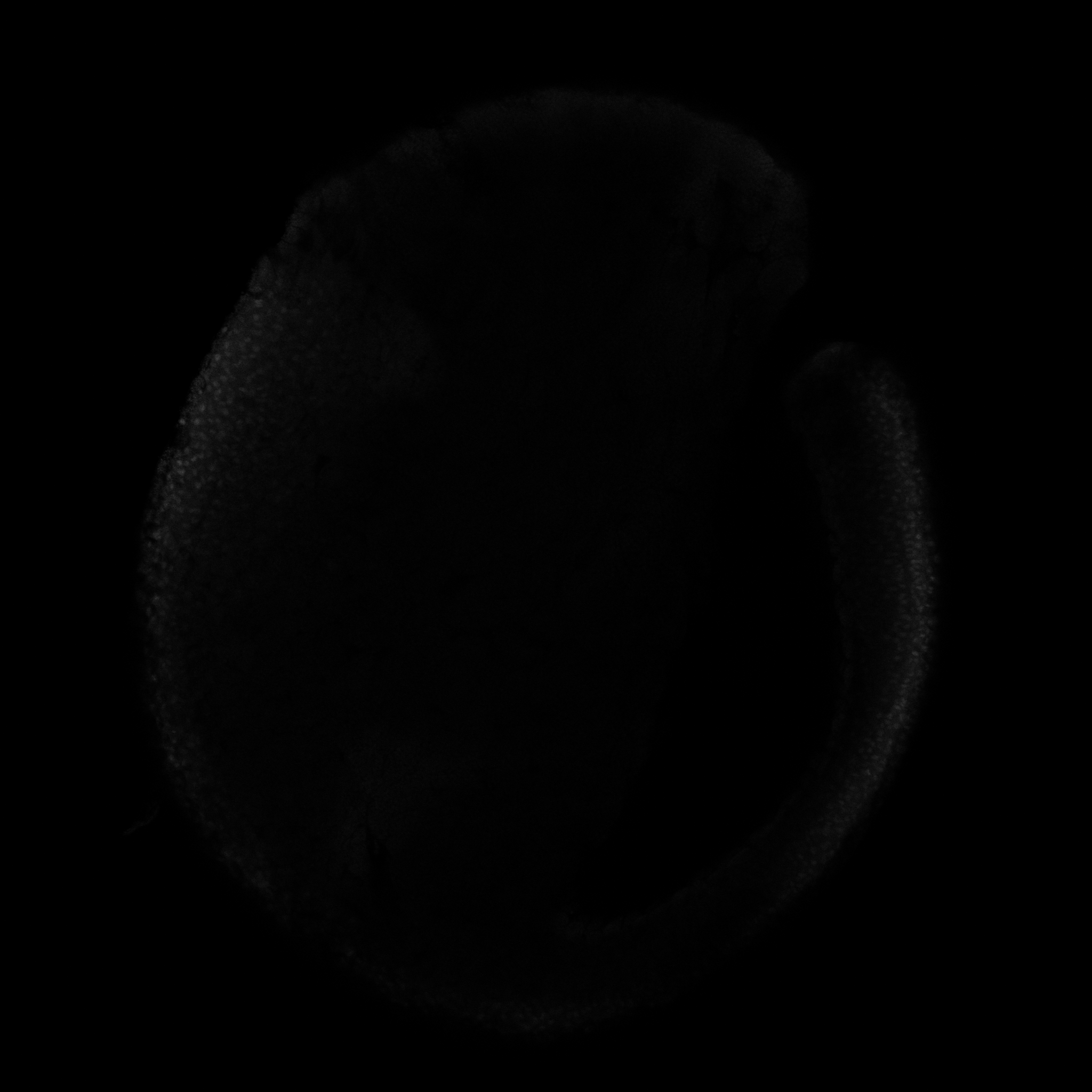

Supplement: Supplementary file 7 — Source data Fig. 3 [file 44319_2025_617_MOESM7_ESM.zip › Figure 3/Figure 3A/sox8 CRISPR.tif]

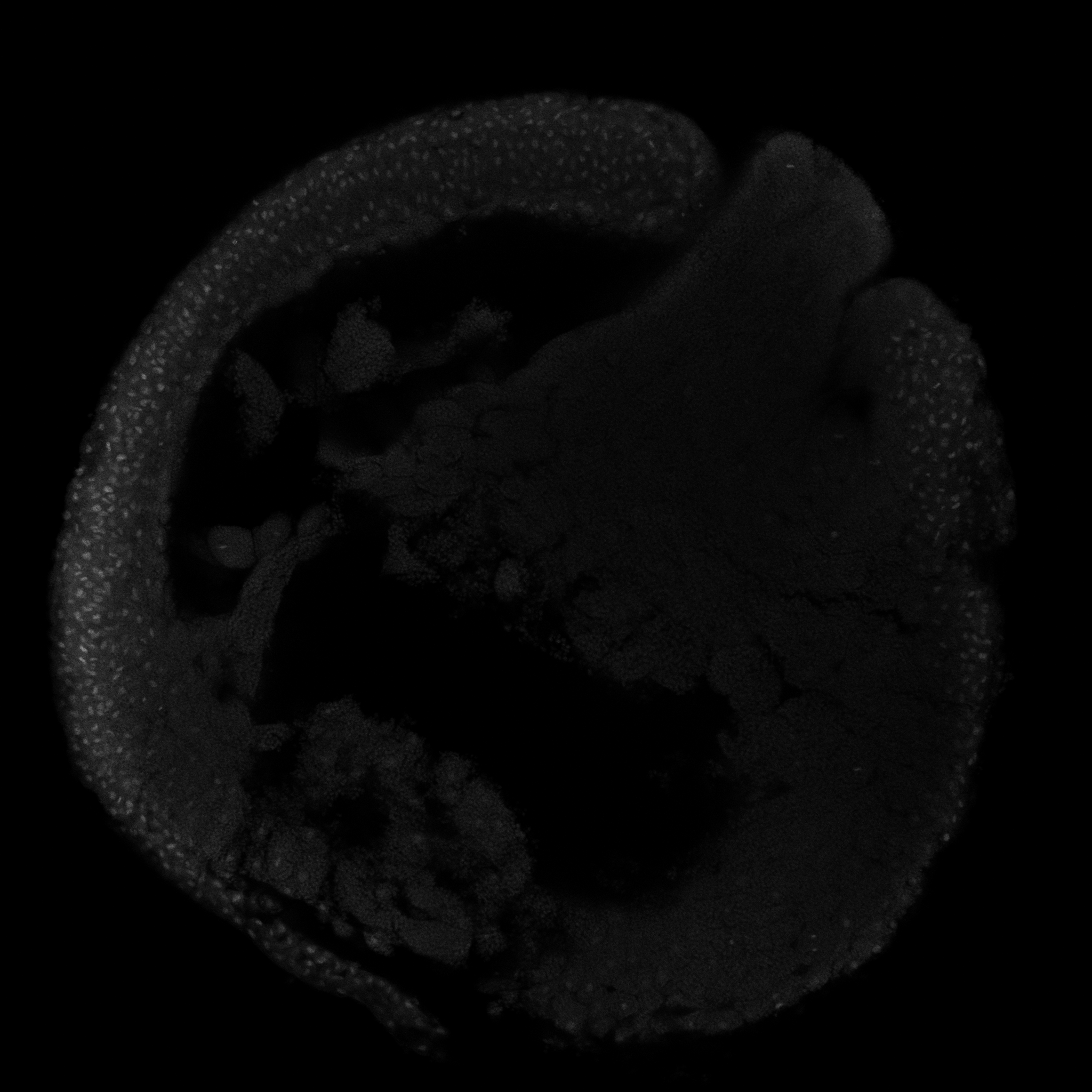

Supplement: Supplementary file 7 — Source data Fig. 3 [file 44319_2025_617_MOESM7_ESM.zip › Figure 3/Figure 3A/Control.tif]

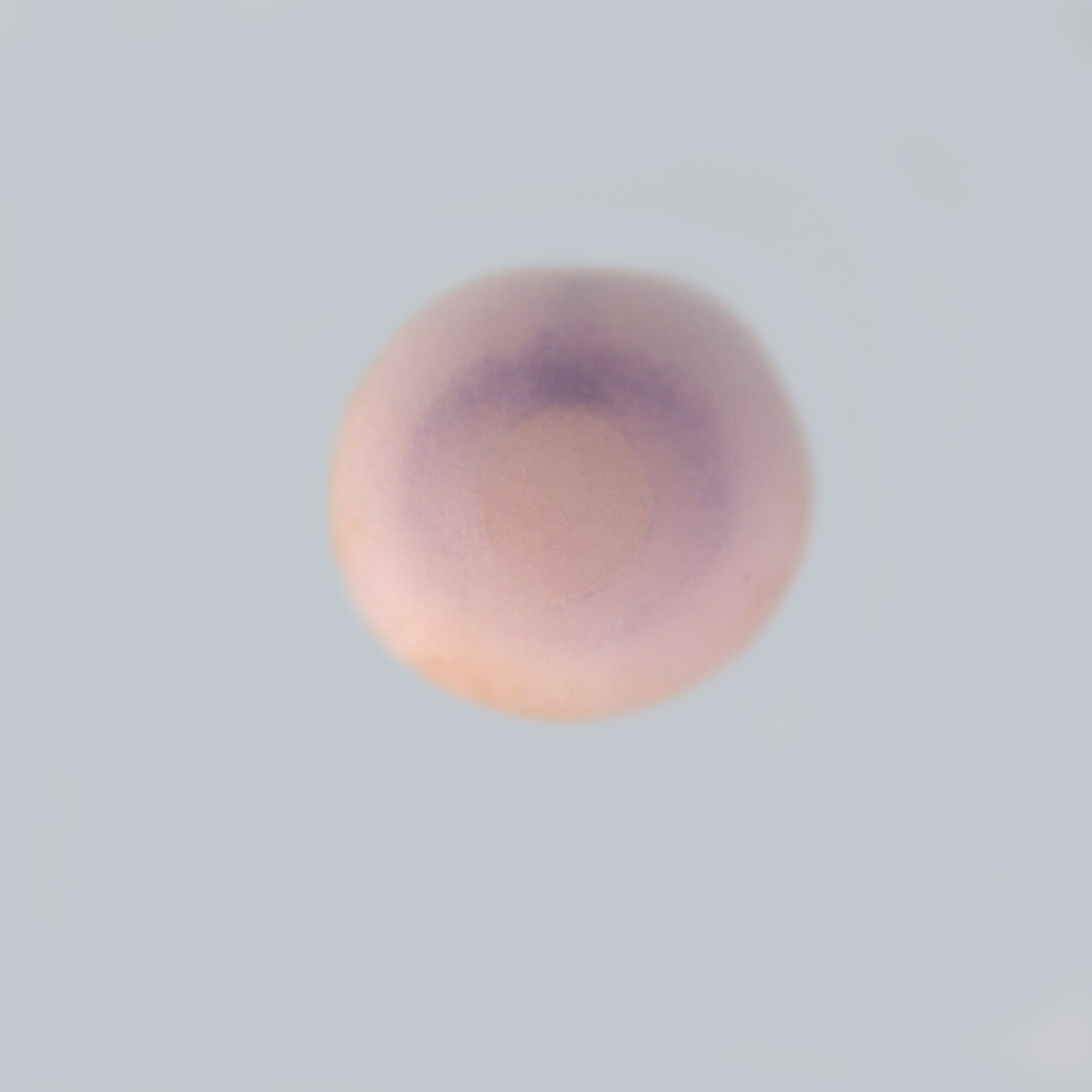

Supplement: Supplementary file 8 — Source data Fig. 4 [file 44319_2025_617_MOESM8_ESM.zip › Figure 4/Figure 4G/Control.jpg]

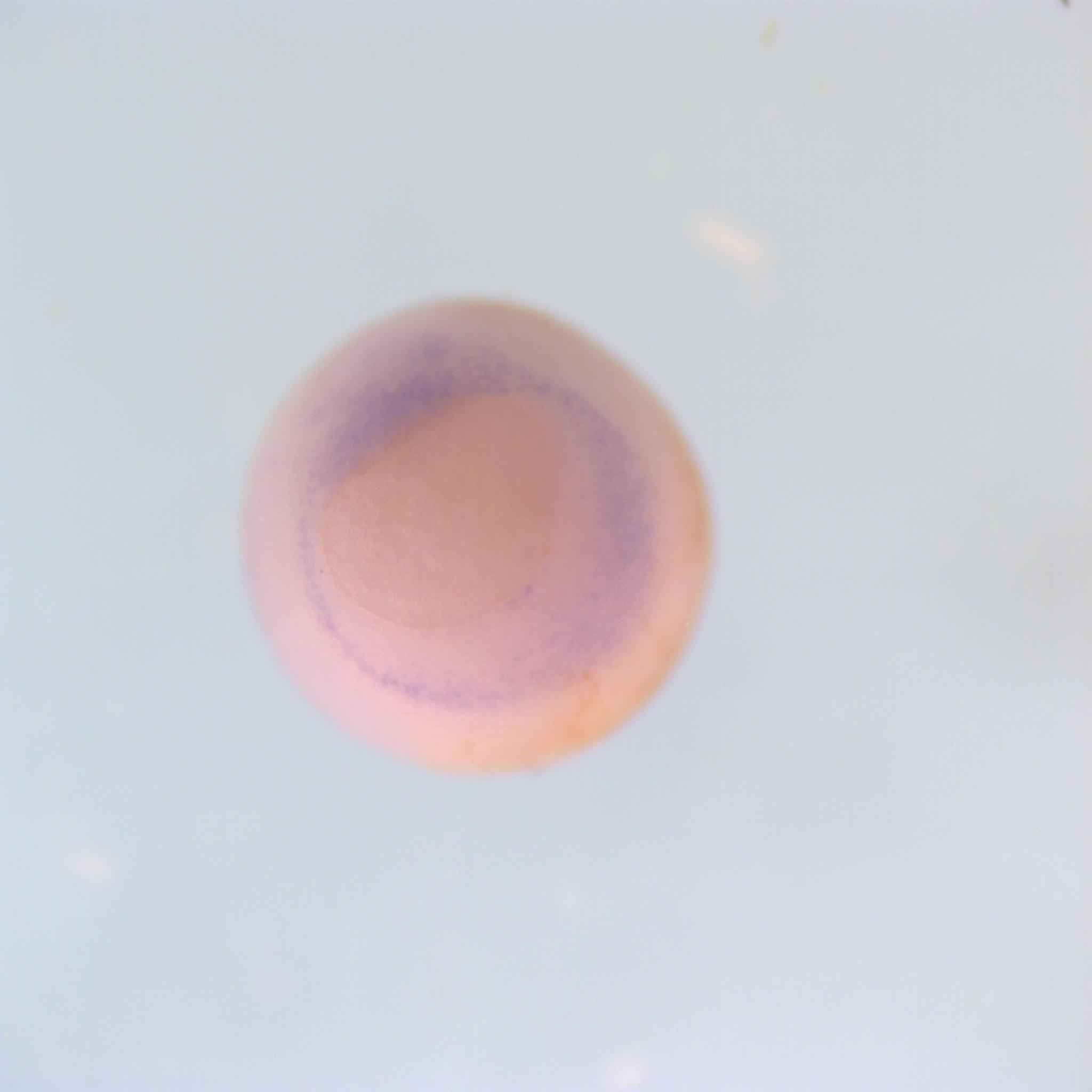

Supplement: Supplementary file 8 — Source data Fig. 4 [file 44319_2025_617_MOESM8_ESM.zip › Figure 4/Figure 4G/sox8 CRISPR.jpg]

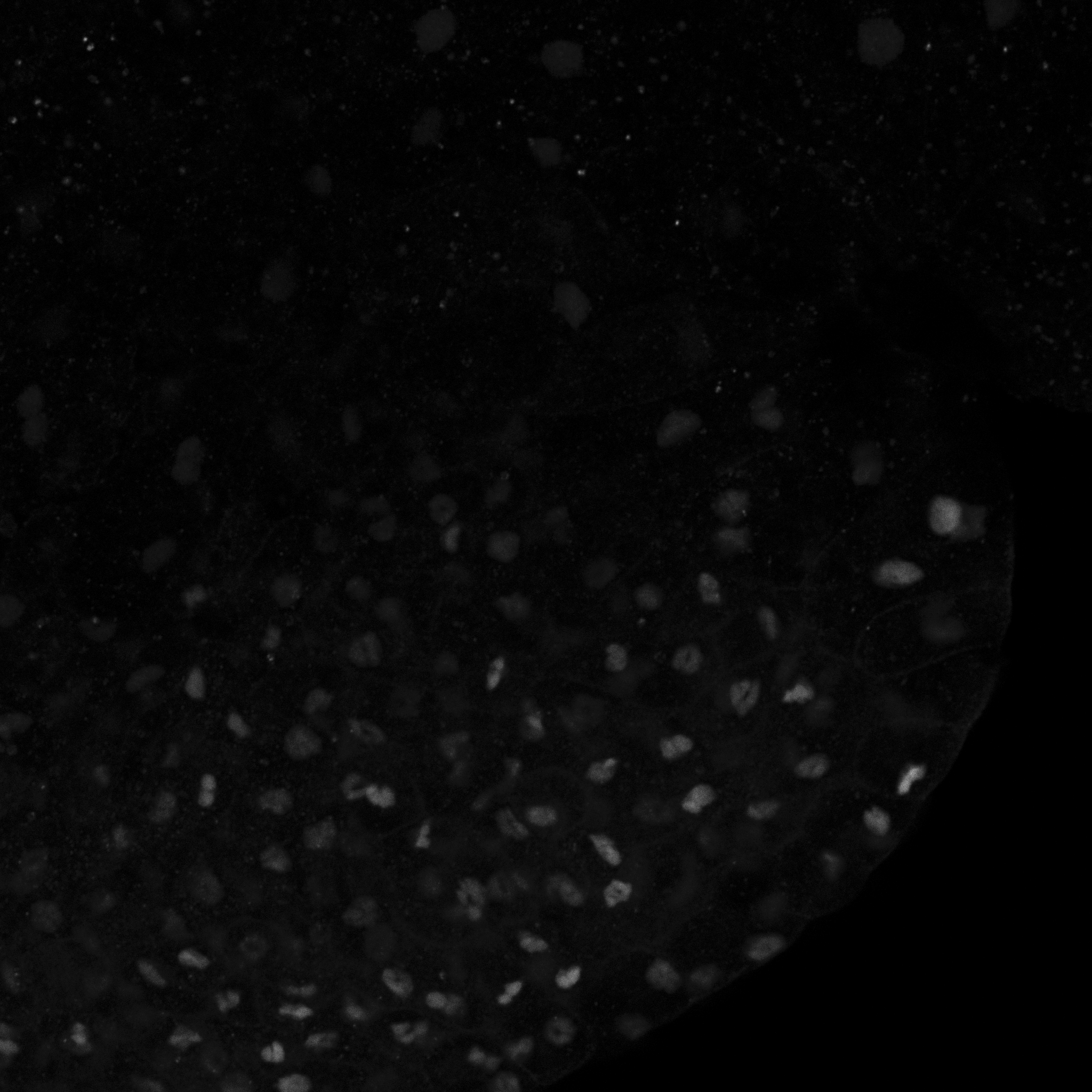

Supplement: Supplementary file 8 — Source data Fig. 4 [file 44319_2025_617_MOESM8_ESM.zip › Figure 4/Figure4K,L/Sox8 CRISPR/sox8 CRISPR.tif]

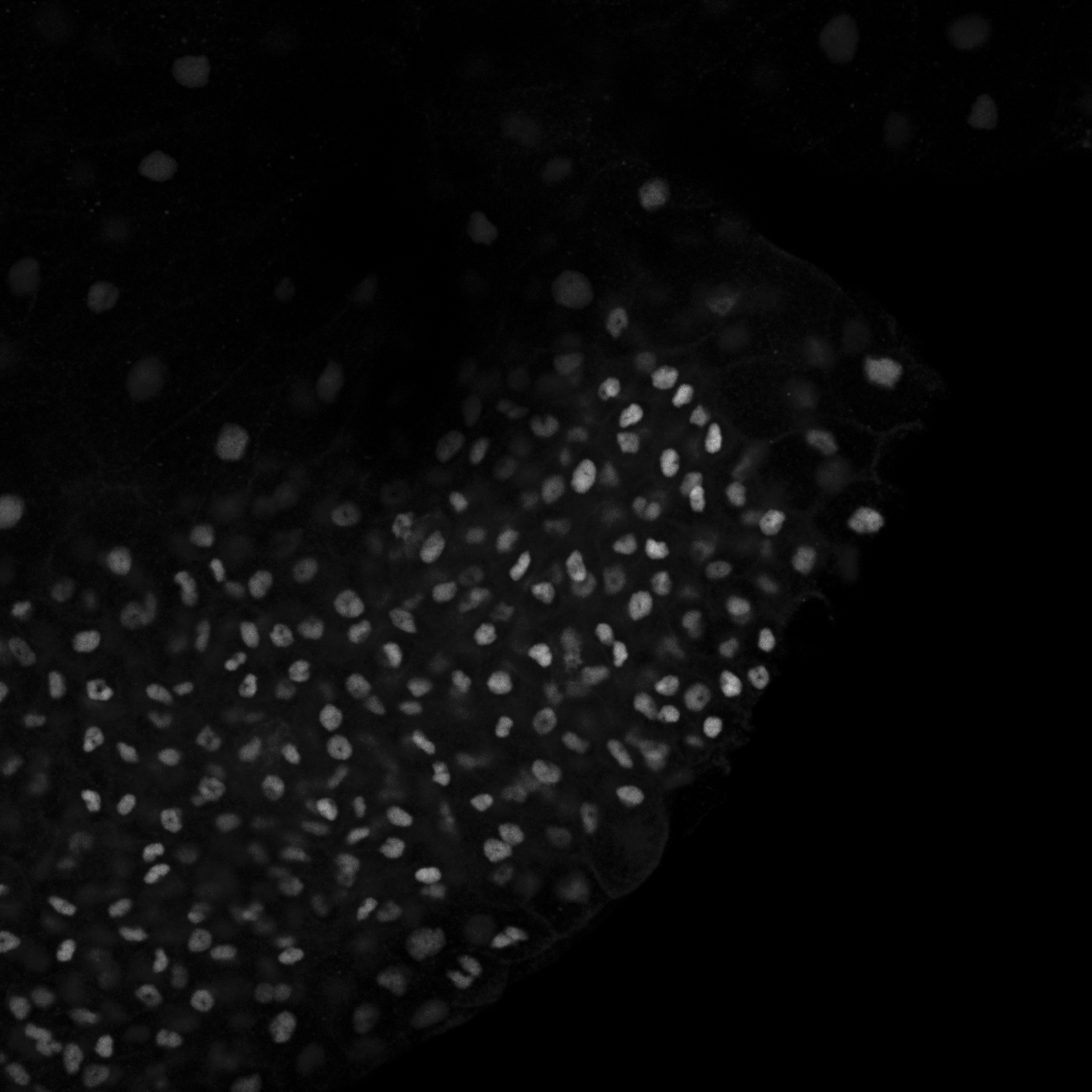

Supplement: Supplementary file 8 — Source data Fig. 4 [file 44319_2025_617_MOESM8_ESM.zip › Figure 4/Figure4K,L/Control/Control_psmad1_single plan.tif]

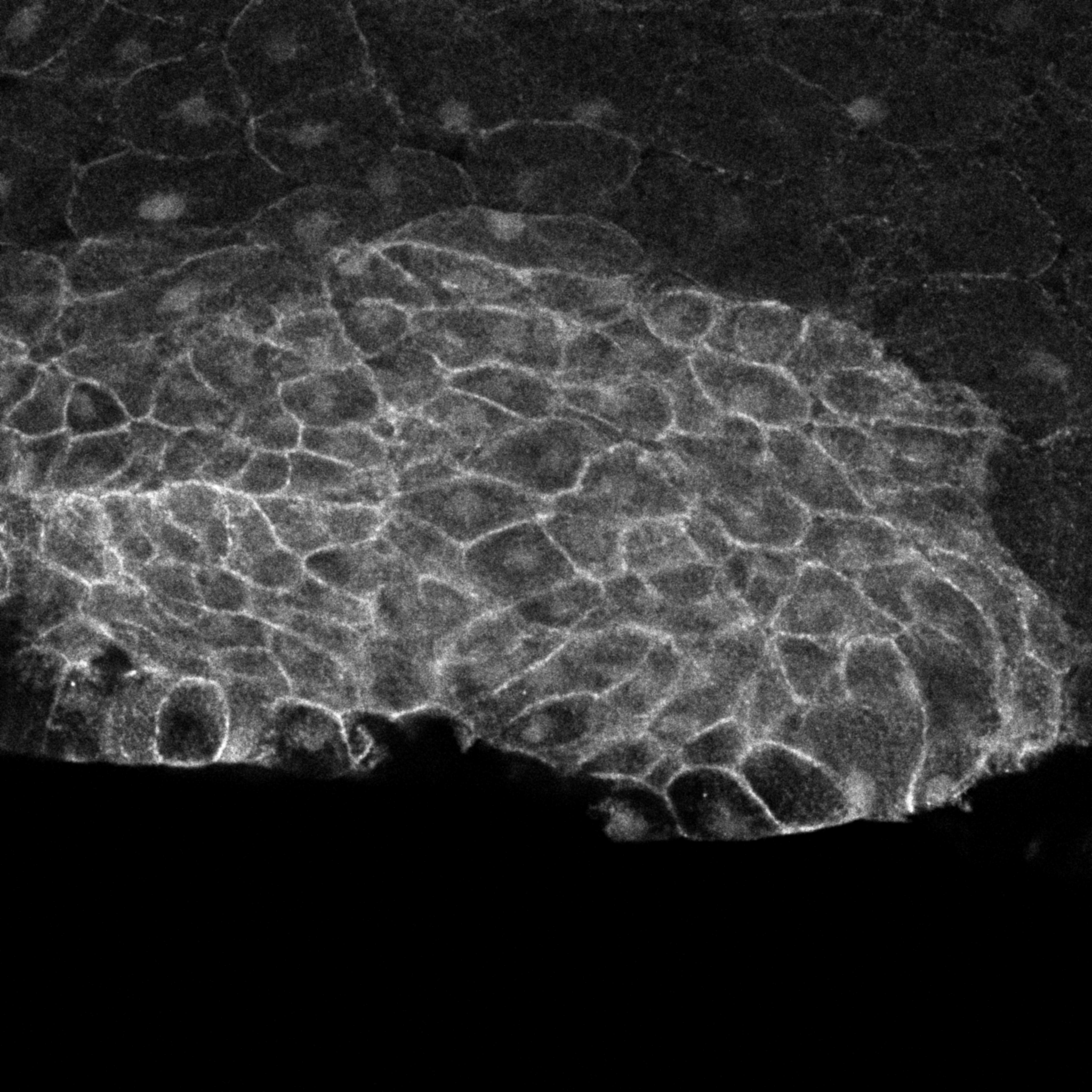

Supplement: Supplementary file 8 — Source data Fig. 4 [file 44319_2025_617_MOESM8_ESM.zip › Figure 4/Figure 4I, J/sox8 CRISPR/sox8 CRISPR_Beta catenin_single plan.tif]

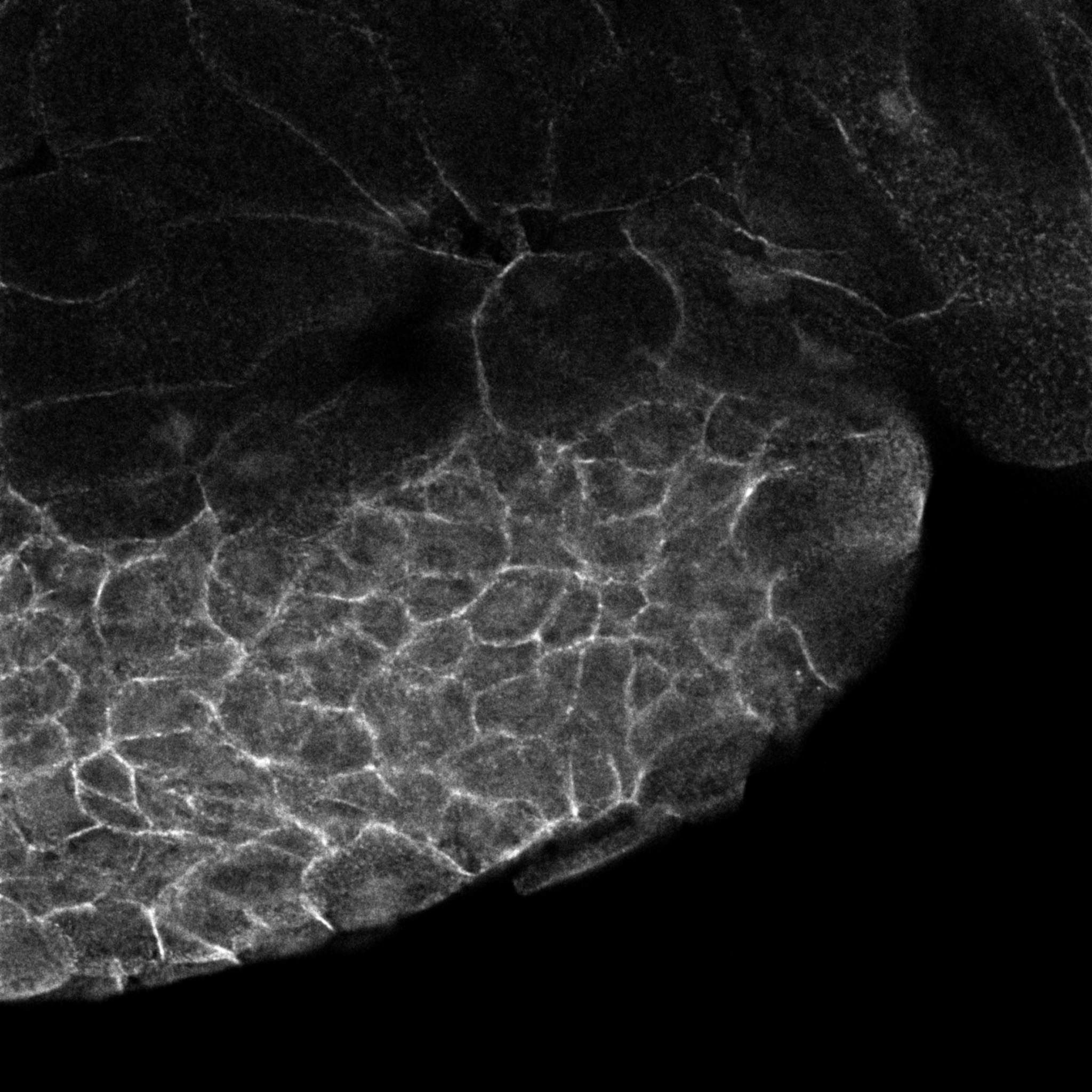

Supplement: Supplementary file 8 — Source data Fig. 4 [file 44319_2025_617_MOESM8_ESM.zip › Figure 4/Figure 4I, J/Control/Control_Beta catenin_single plan.tif]

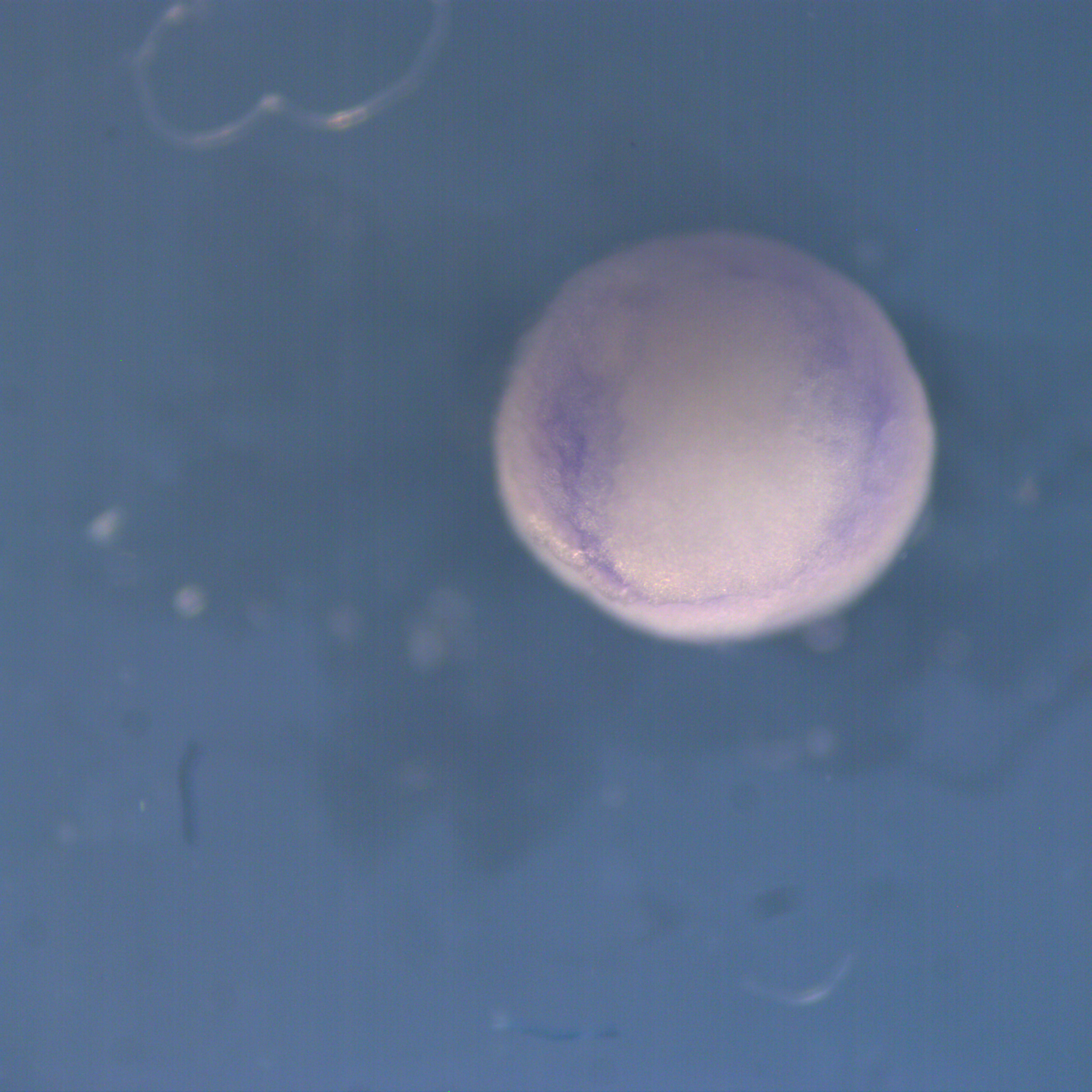

Supplement: Supplementary file 9 — Source data Fig. 5 [file 44319_2025_617_MOESM9_ESM.zip › Figure 5/Figure 5A/sox8 CRISPR_ISH Kremen.png]

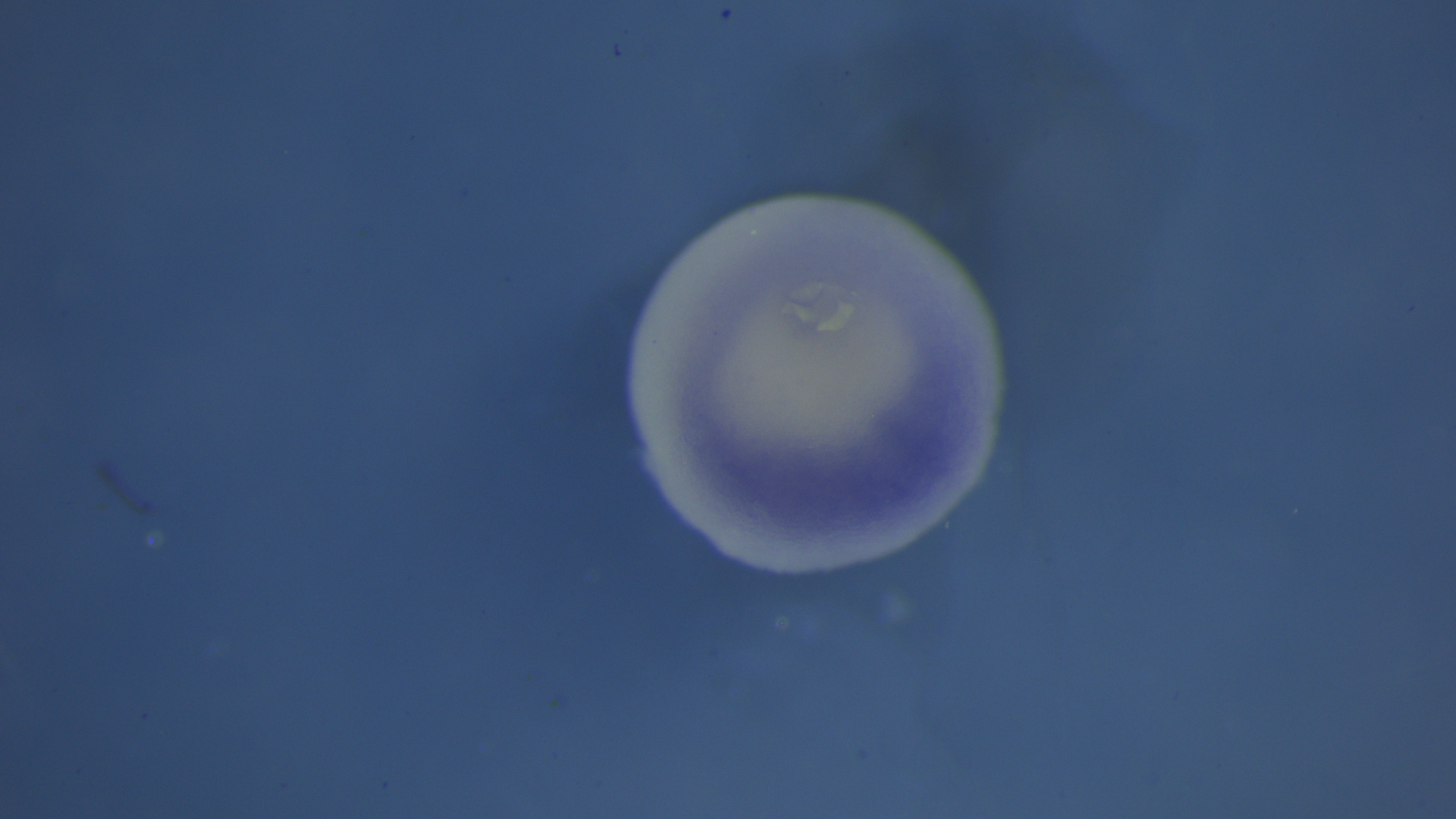

Supplement: Supplementary file 9 — Source data Fig. 5 [file 44319_2025_617_MOESM9_ESM.zip › Figure 5/Figure 5A/Control_ISH kremen.tif]

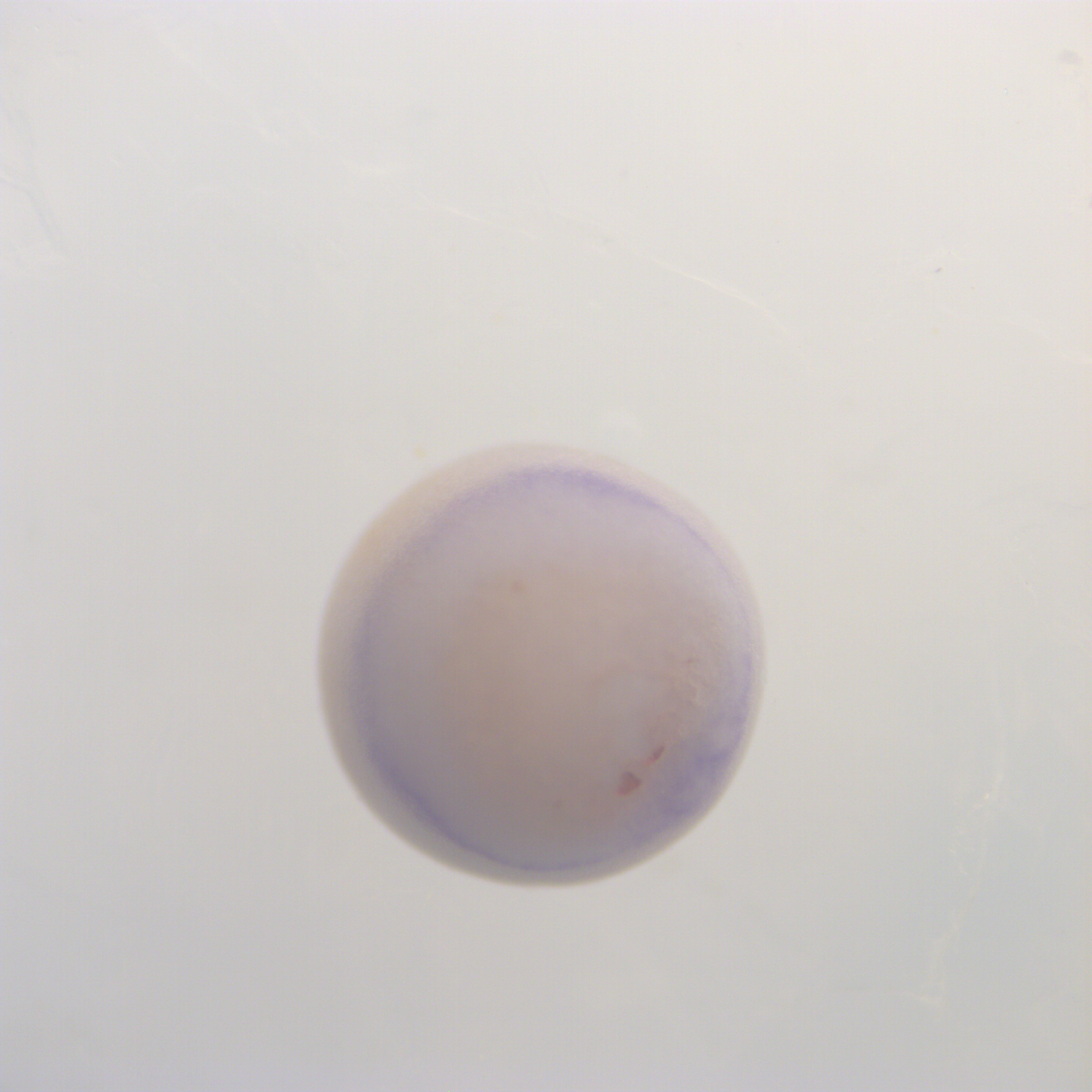

Supplement: Supplementary file 10 — Source data Fig. 6 [file 44319_2025_617_MOESM10_ESM.zip › Figure 6/Figure 6D/Kremen2 CRISPR_Wnt11b.png]

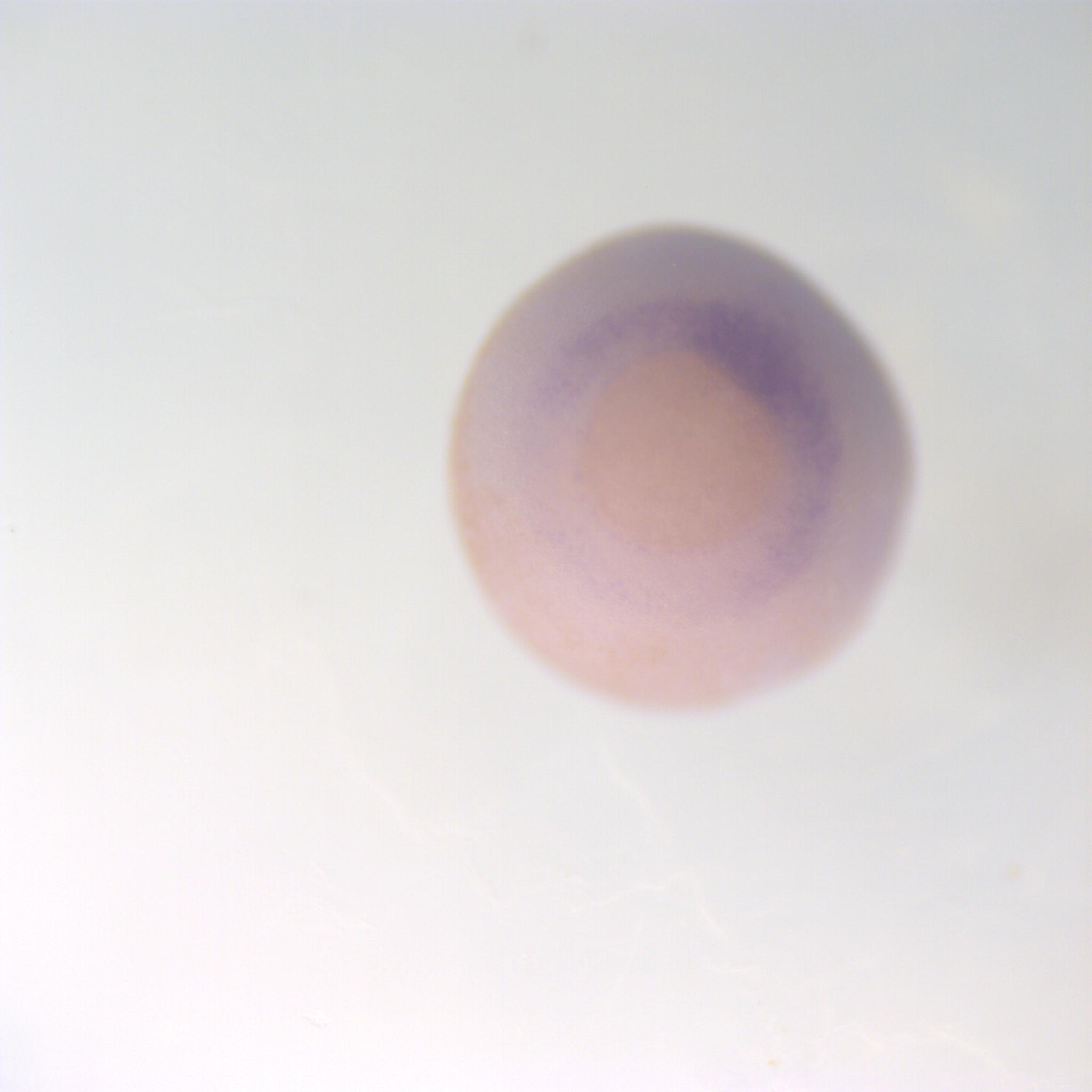

Supplement: Supplementary file 10 — Source data Fig. 6 [file 44319_2025_617_MOESM10_ESM.zip › Figure 6/Figure 6D/Control_Wnt11b.jpg]

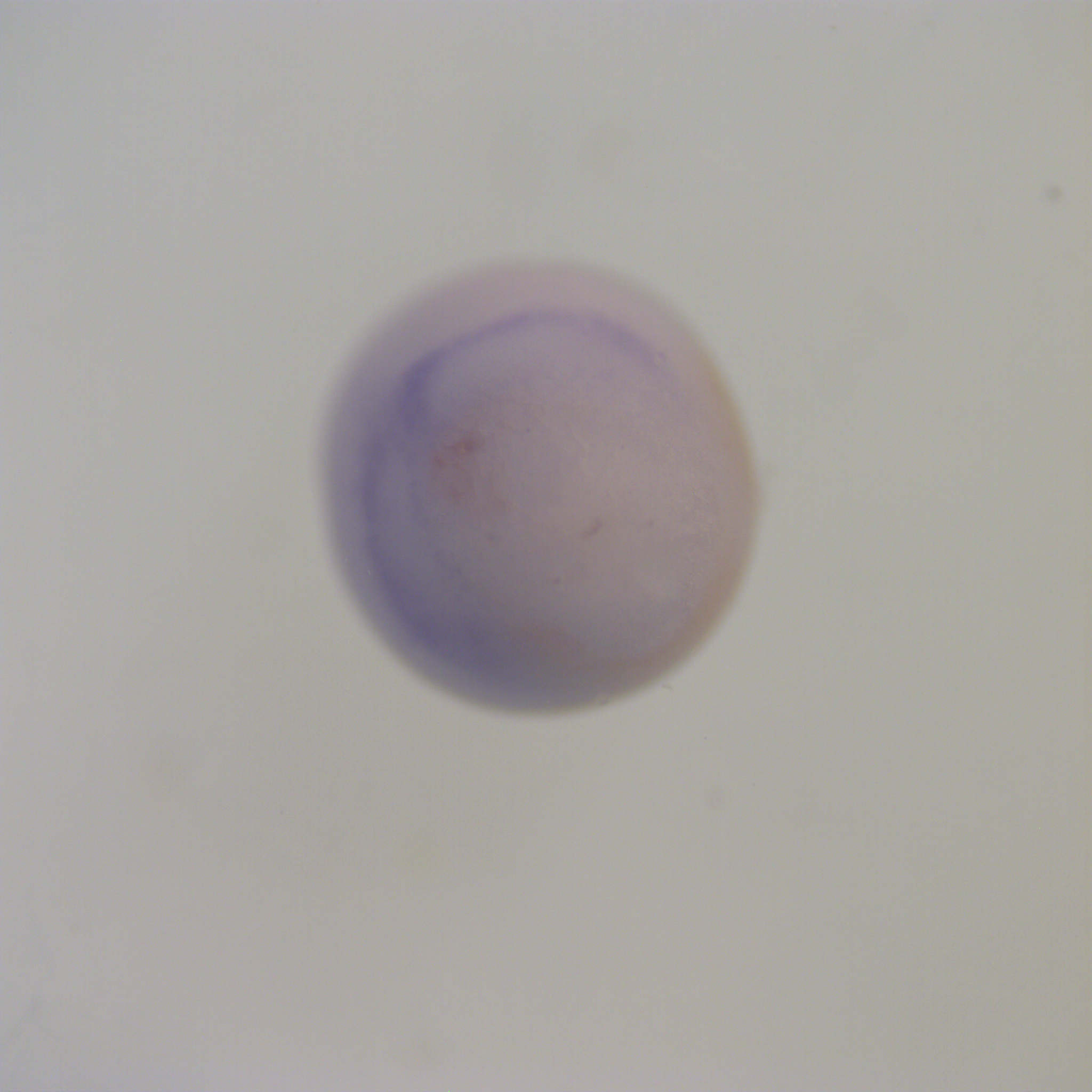

Supplement: Supplementary file 10 — Source data Fig. 6 [file 44319_2025_617_MOESM10_ESM.zip › Figure 6/Figure 6D/Kremen2 CRISPR +mRNA Kremen2_Wnt11b.png]

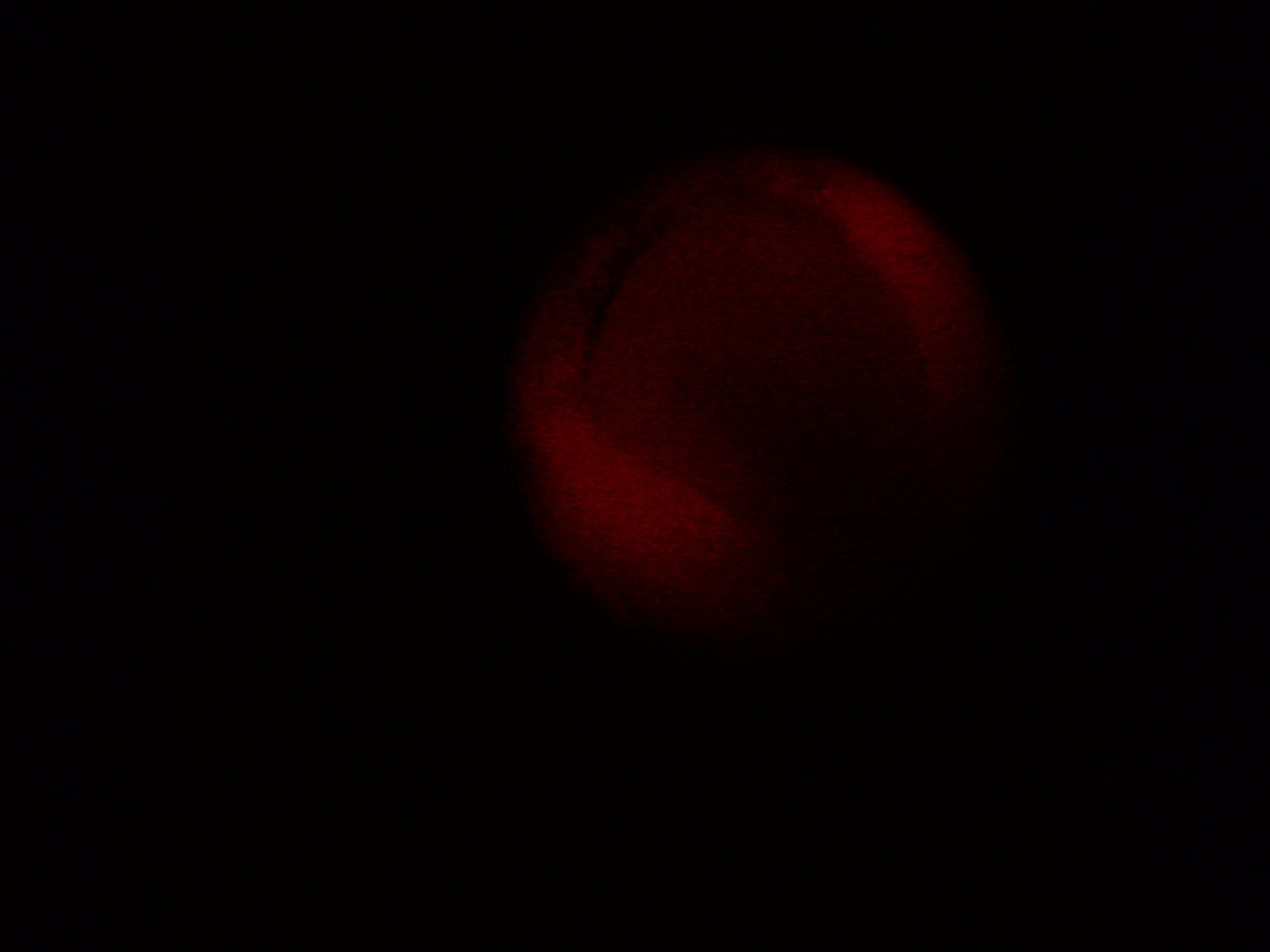

Supplement: Supplementary file 10 — Source data Fig. 6 [file 44319_2025_617_MOESM10_ESM.zip › Figure 6/Figure 6A/kremen2 CRISPR/A028 - 20240416_171248.bmp]

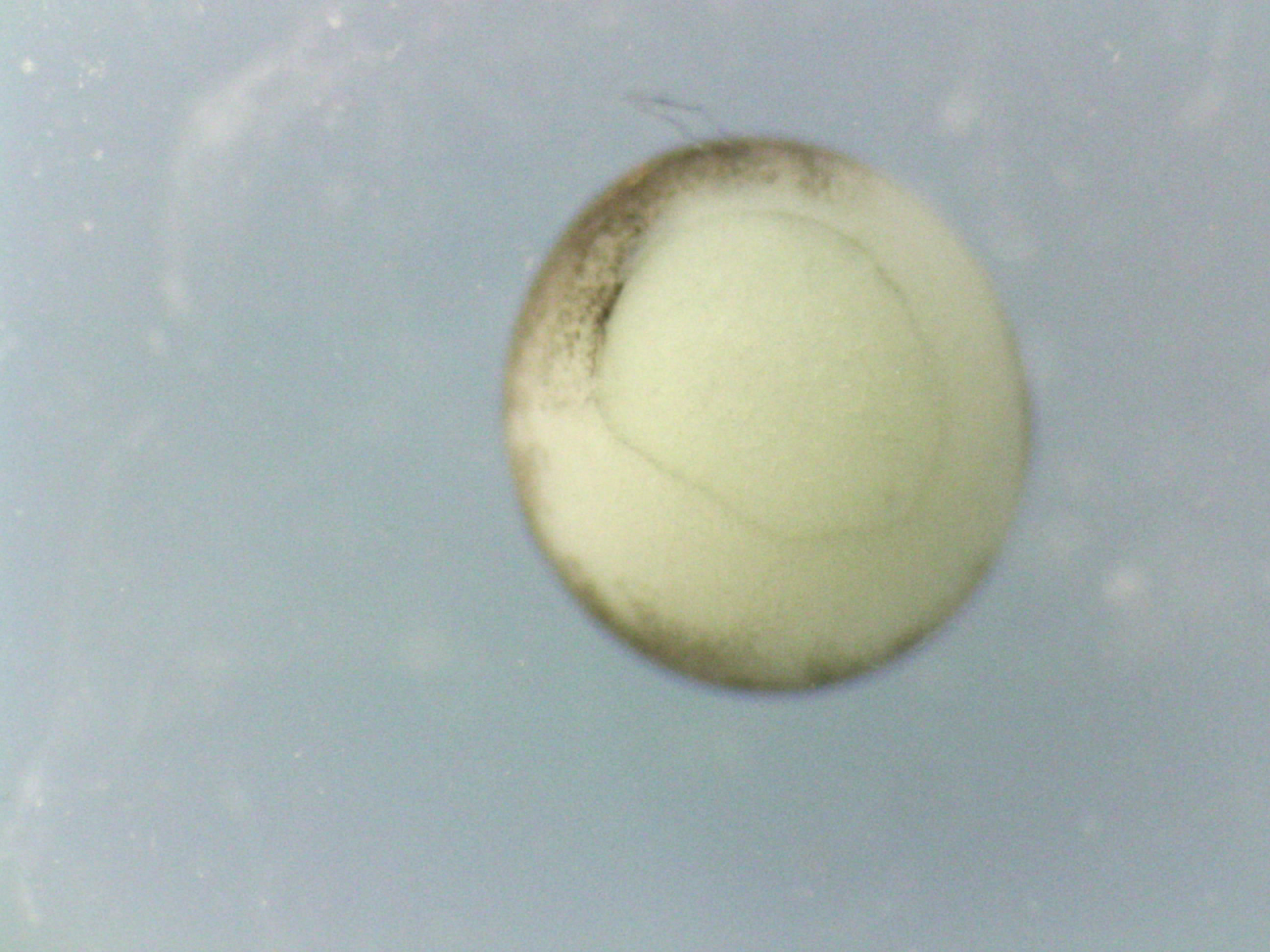

Supplement: Supplementary file 10 — Source data Fig. 6 [file 44319_2025_617_MOESM10_ESM.zip › Figure 6/Figure 6A/kremen2 CRISPR/A027 - 20240416_171240.bmp]

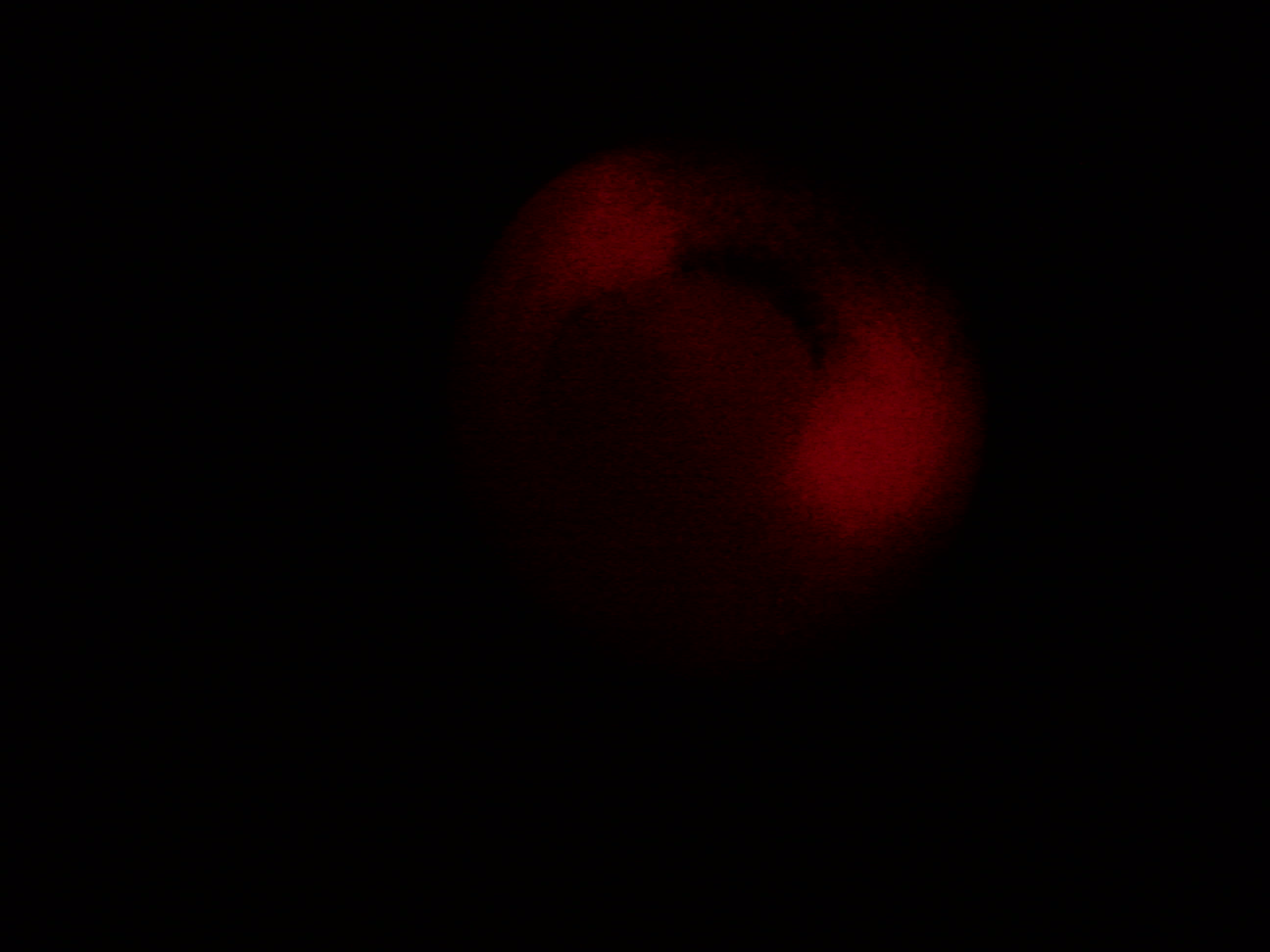

Supplement: Supplementary file 10 — Source data Fig. 6 [file 44319_2025_617_MOESM10_ESM.zip › Figure 6/Figure 6A/kremen2 CRISPR + mRNA kremen2/A067 - 20240416_172514.bmp]

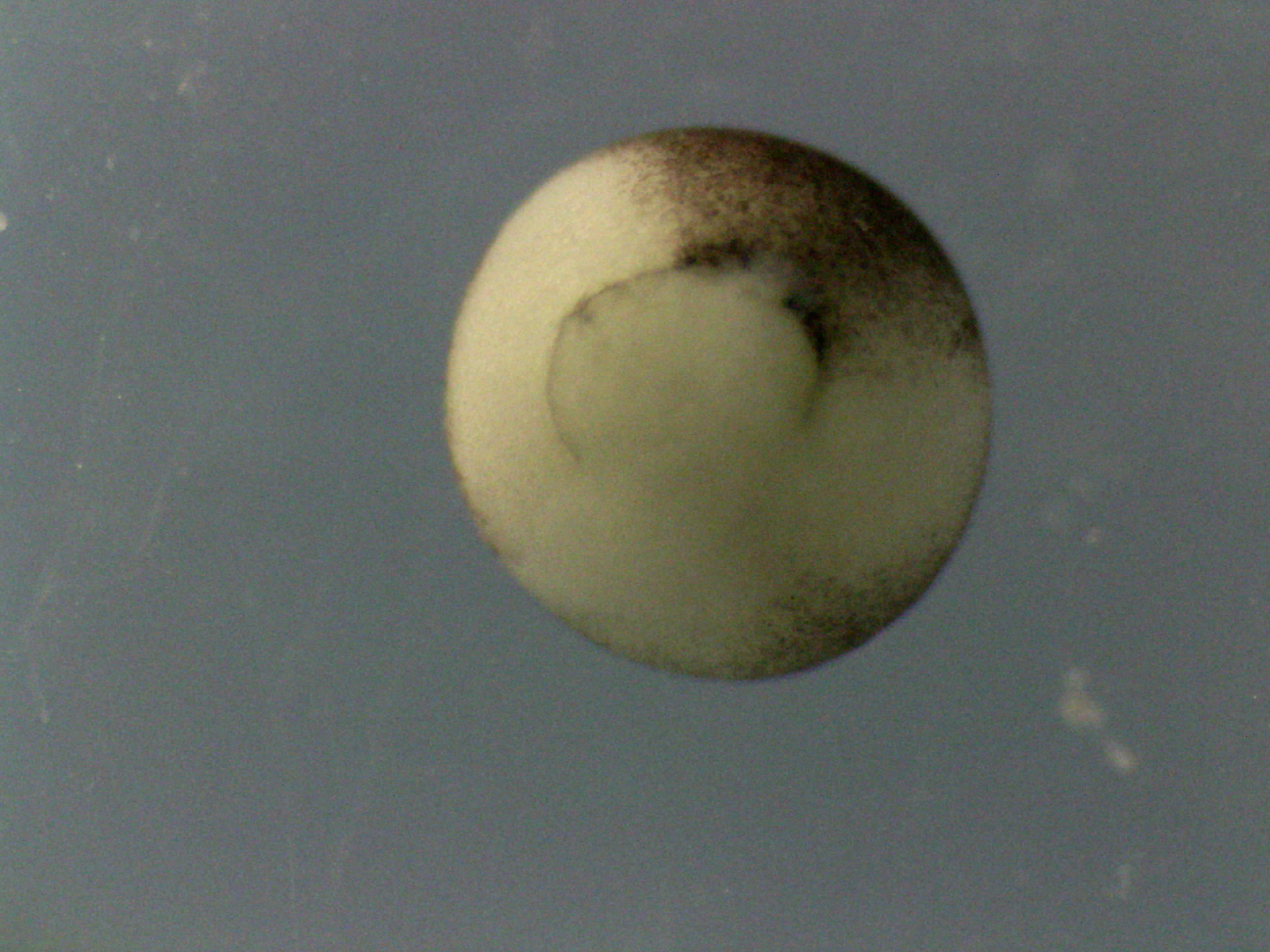

Supplement: Supplementary file 10 — Source data Fig. 6 [file 44319_2025_617_MOESM10_ESM.zip › Figure 6/Figure 6A/kremen2 CRISPR + mRNA kremen2/A066 - 20240416_172510.bmp]

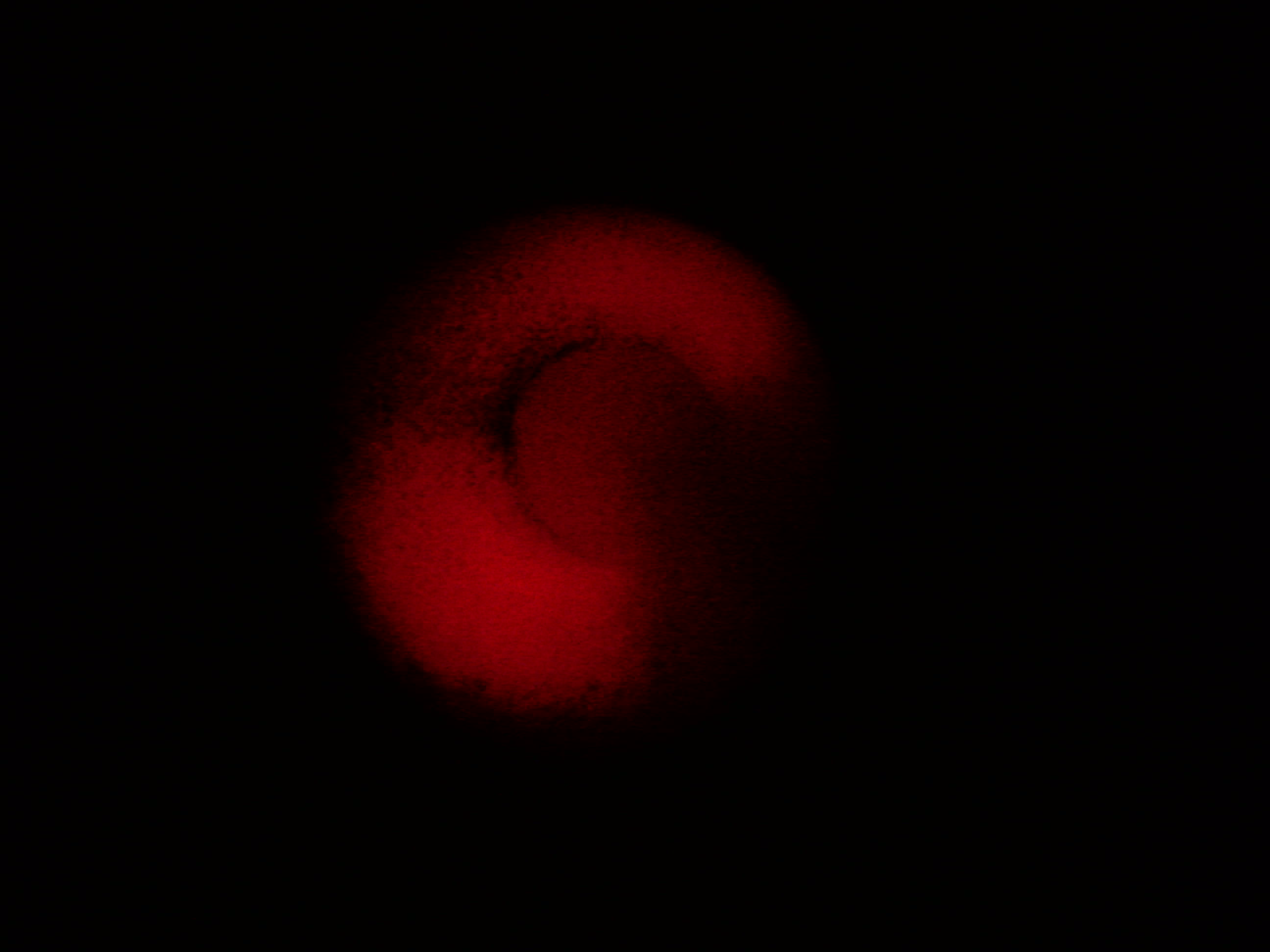

Supplement: Supplementary file 10 — Source data Fig. 6 [file 44319_2025_617_MOESM10_ESM.zip › Figure 6/Figure 6A/Control/A014 - 20240416_170653.bmp]

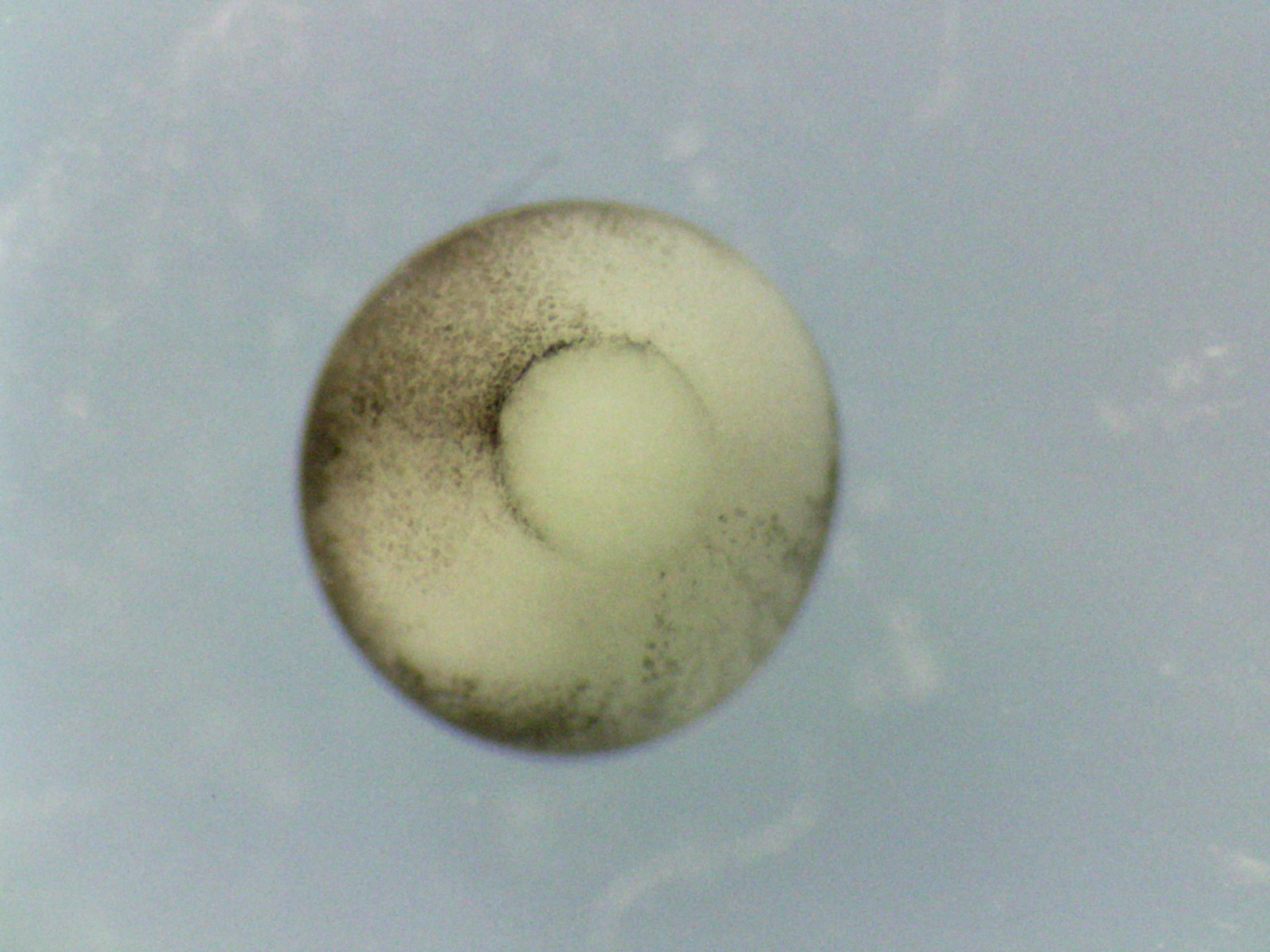

Supplement: Supplementary file 10 — Source data Fig. 6 [file 44319_2025_617_MOESM10_ESM.zip › Figure 6/Figure 6A/Control/A013 - 20240416_170642.bmp]

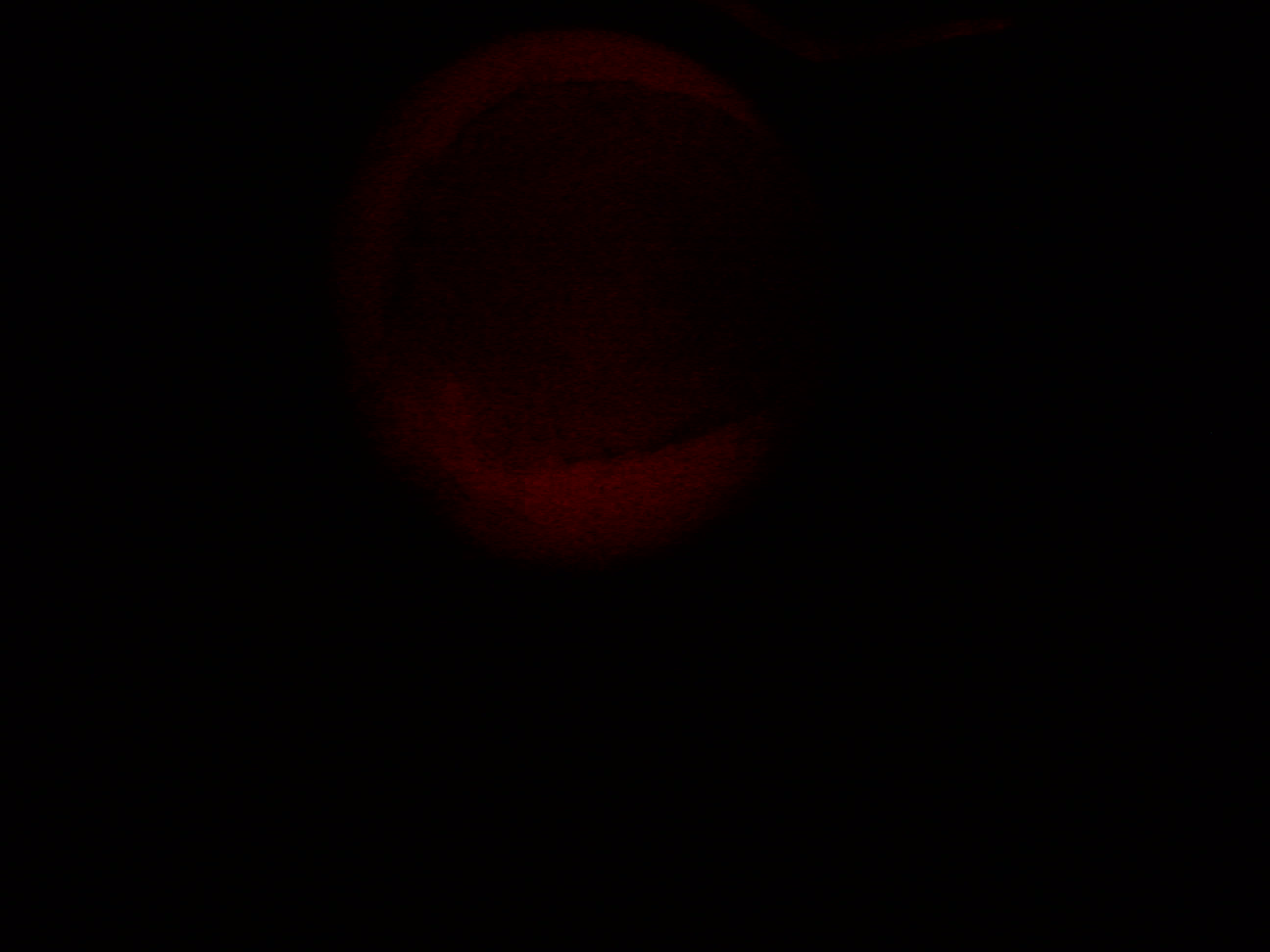

Supplement: Supplementary file 10 — Source data Fig. 6 [file 44319_2025_617_MOESM10_ESM.zip › Figure 6/Figure 6G-L/Fig 6L_Kremen2 CRISPR mRNA sox8/A119 - 20240416_174332.bmp]

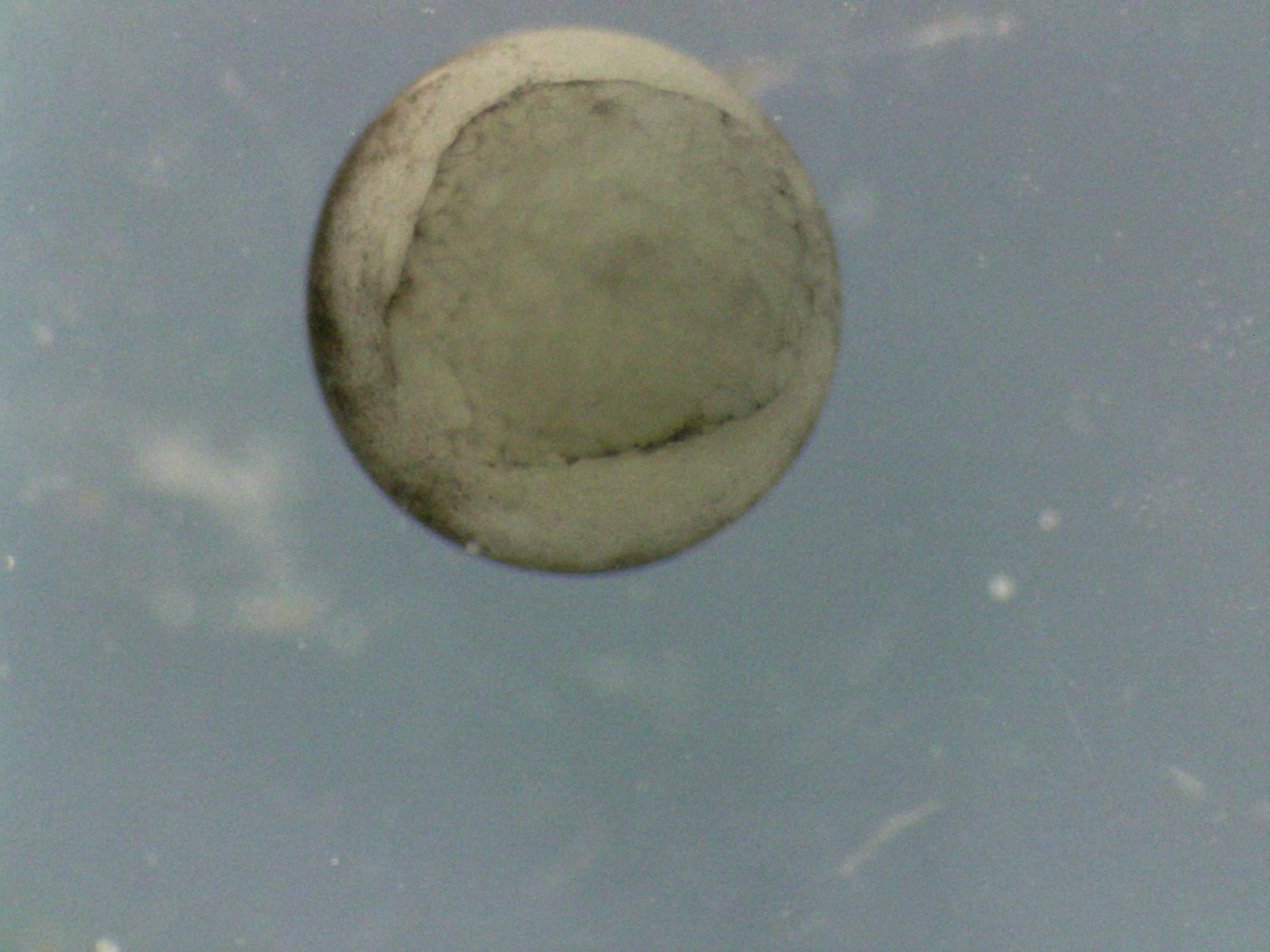

Supplement: Supplementary file 10 — Source data Fig. 6 [file 44319_2025_617_MOESM10_ESM.zip › Figure 6/Figure 6G-L/Fig 6L_Kremen2 CRISPR mRNA sox8/A118 - 20240416_174327.bmp]

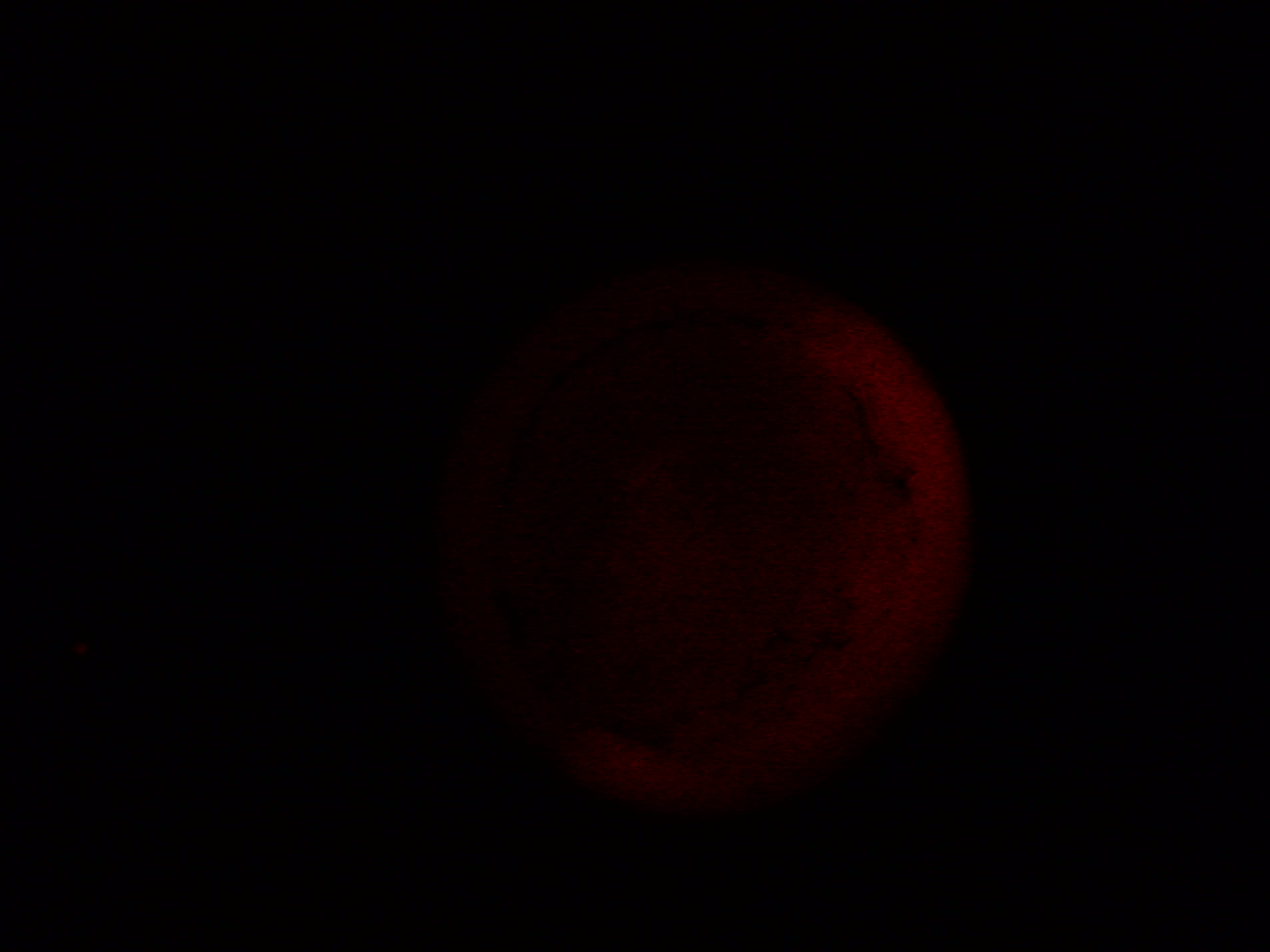

Supplement: Supplementary file 10 — Source data Fig. 6 [file 44319_2025_617_MOESM10_ESM.zip › Figure 6/Figure 6G-L/Fig 6K_Kremen2 CRISPR/A029 - 20240416_171322.bmp]

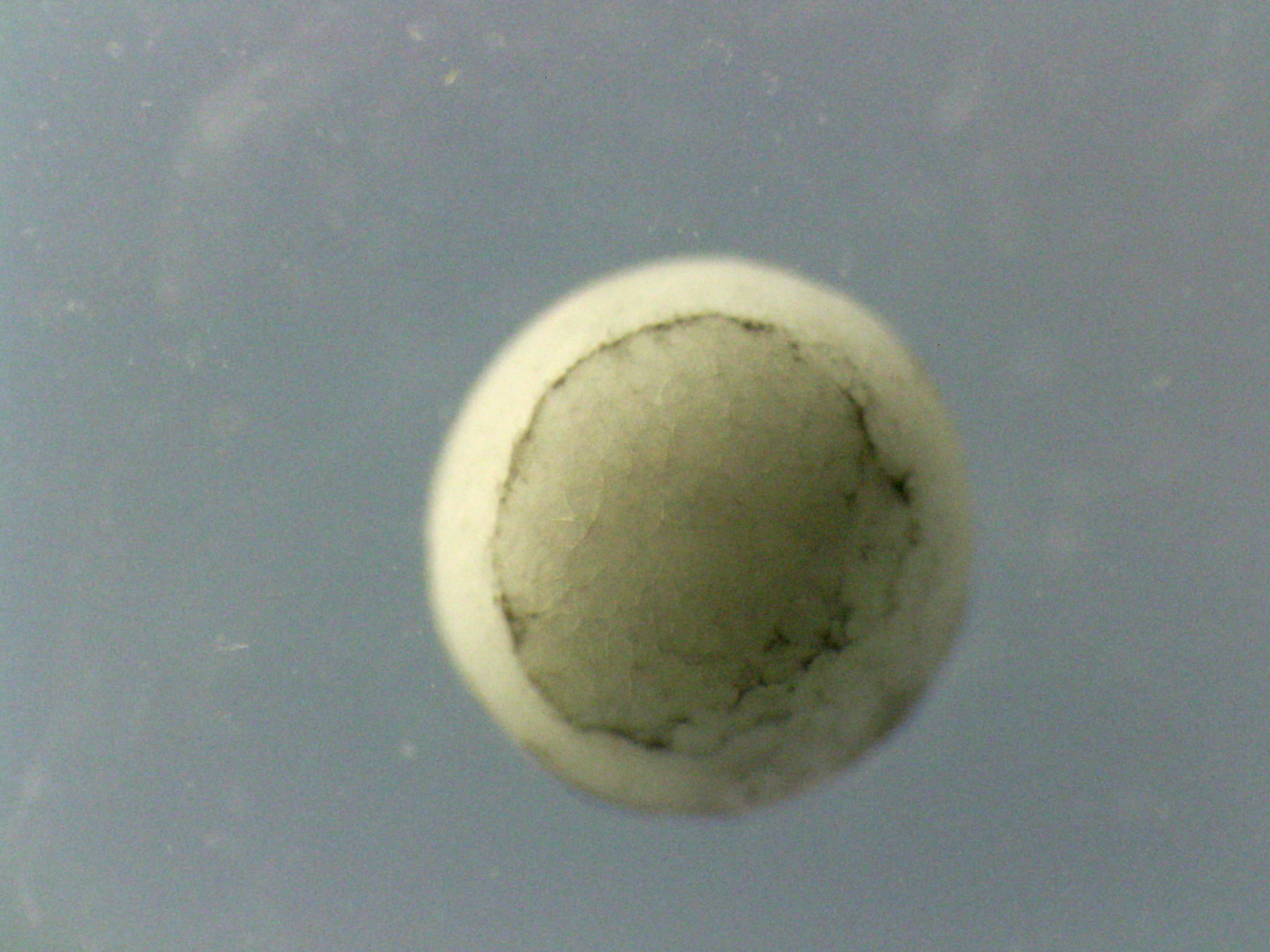

Supplement: Supplementary file 10 — Source data Fig. 6 [file 44319_2025_617_MOESM10_ESM.zip › Figure 6/Figure 6G-L/Fig 6K_Kremen2 CRISPR/A030 - 20240416_171332.bmp]

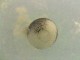

Supplement: Supplementary file 10 — Source data Fig. 6 [file 44319_2025_617_MOESM10_ESM.zip › Figure 6/Figure 6G-L/Fig 6I_sox8 CRISPR mRNA Krm2/A029 - 20240523_162754.jpg]

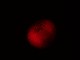

Supplement: Supplementary file 10 — Source data Fig. 6 [file 44319_2025_617_MOESM10_ESM.zip › Figure 6/Figure 6G-L/Fig 6I_sox8 CRISPR mRNA Krm2/A028 - 20240523_162728.jpg]

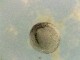

Supplement: Supplementary file 10 — Source data Fig. 6 [file 44319_2025_617_MOESM10_ESM.zip › Figure 6/Figure 6G-L/Fig 6H_sox8 CRISPR/A026 - 20240523_162400.jpg]

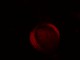

Supplement: Supplementary file 10 — Source data Fig. 6 [file 44319_2025_617_MOESM10_ESM.zip › Figure 6/Figure 6G-L/Fig 6H_sox8 CRISPR/A027 - 20240523_162406.jpg]

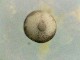

Supplement: Supplementary file 10 — Source data Fig. 6 [file 44319_2025_617_MOESM10_ESM.zip › Figure 6/Figure 6G-L/Fig 6G_Control/A022 - 20240523_162020.jpg]

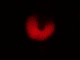

Supplement: Supplementary file 10 — Source data Fig. 6 [file 44319_2025_617_MOESM10_ESM.zip › Figure 6/Figure 6G-L/Fig 6G_Control/A021 - 20240523_162002.jpg]

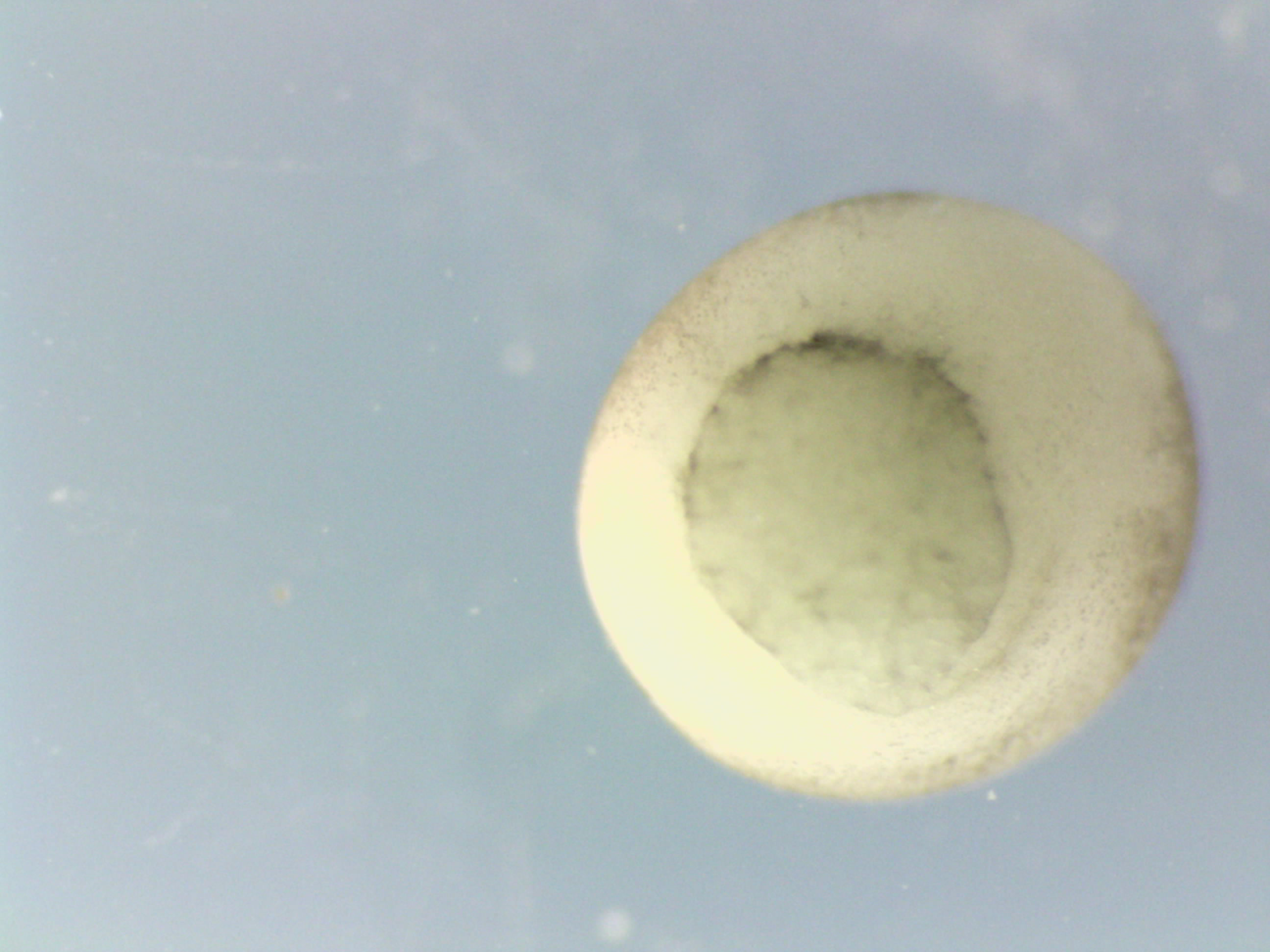

Supplement: Supplementary file 10 — Source data Fig. 6 [file 44319_2025_617_MOESM10_ESM.zip › Figure 6/Figure 6G-L/Fig 6J_Sox8 CRISPR mRNA BMP7/A104 - 20240423_150847.bmp]

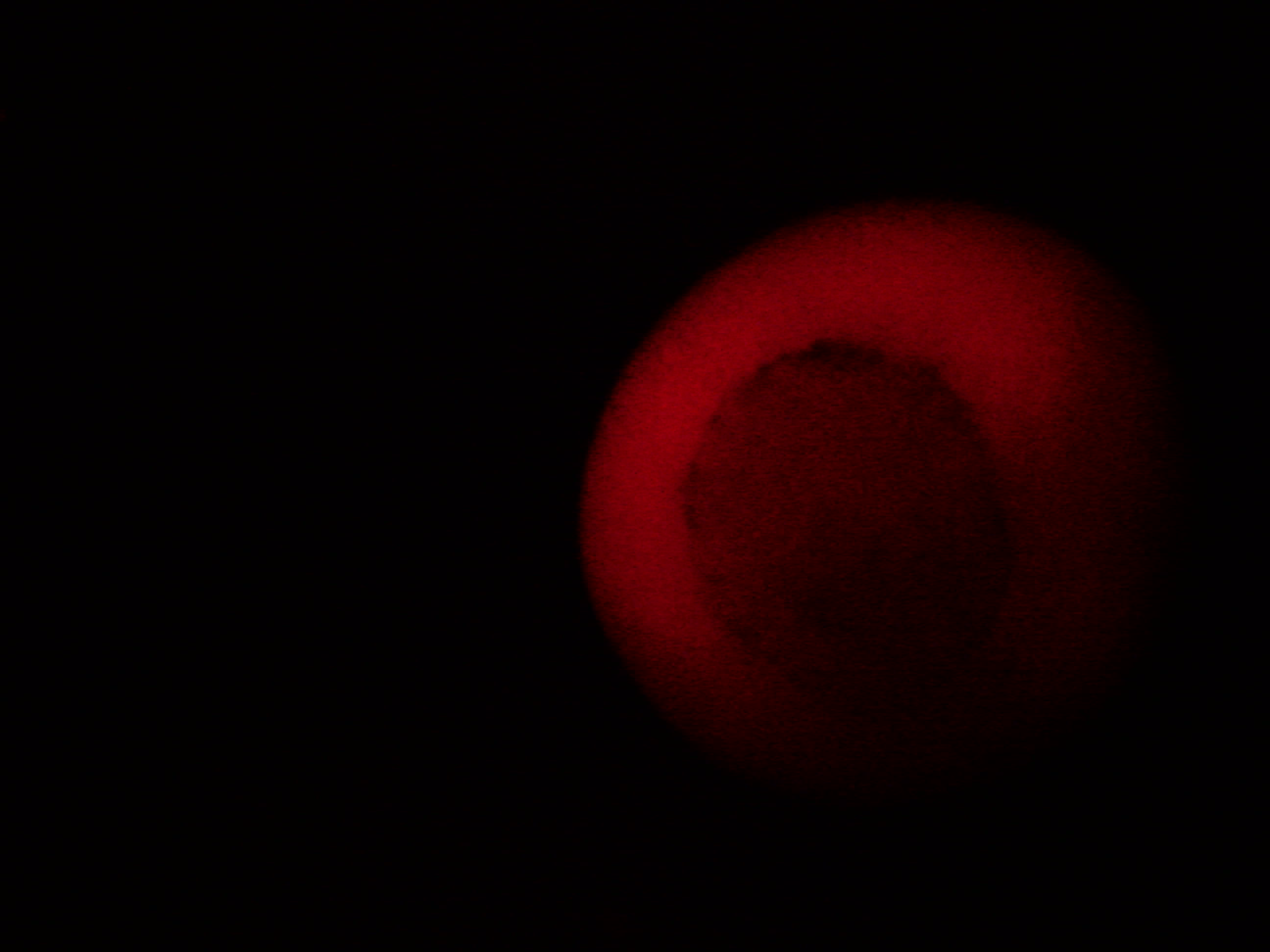

Supplement: Supplementary file 10 — Source data Fig. 6 [file 44319_2025_617_MOESM10_ESM.zip › Figure 6/Figure 6G-L/Fig 6J_Sox8 CRISPR mRNA BMP7/A105 - 20240423_150855.bmp]

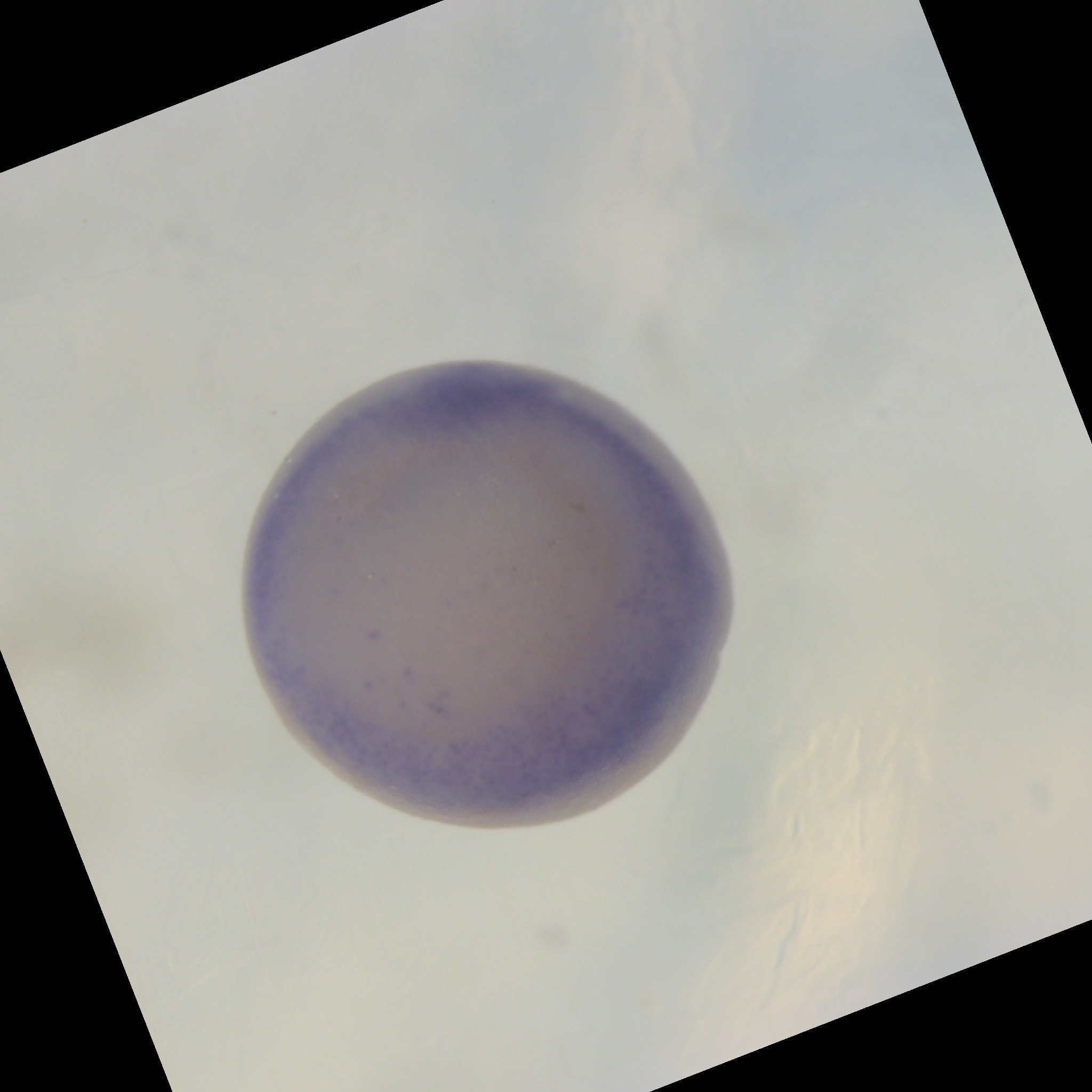

Supplement: Supplementary file 10 — Source data Fig. 6 [file 44319_2025_617_MOESM10_ESM.zip › Figure 6/Figure 6M-Q/Figure 6Q_Kremen2 CRISPR mRNA sox8/6.1_55.tif]

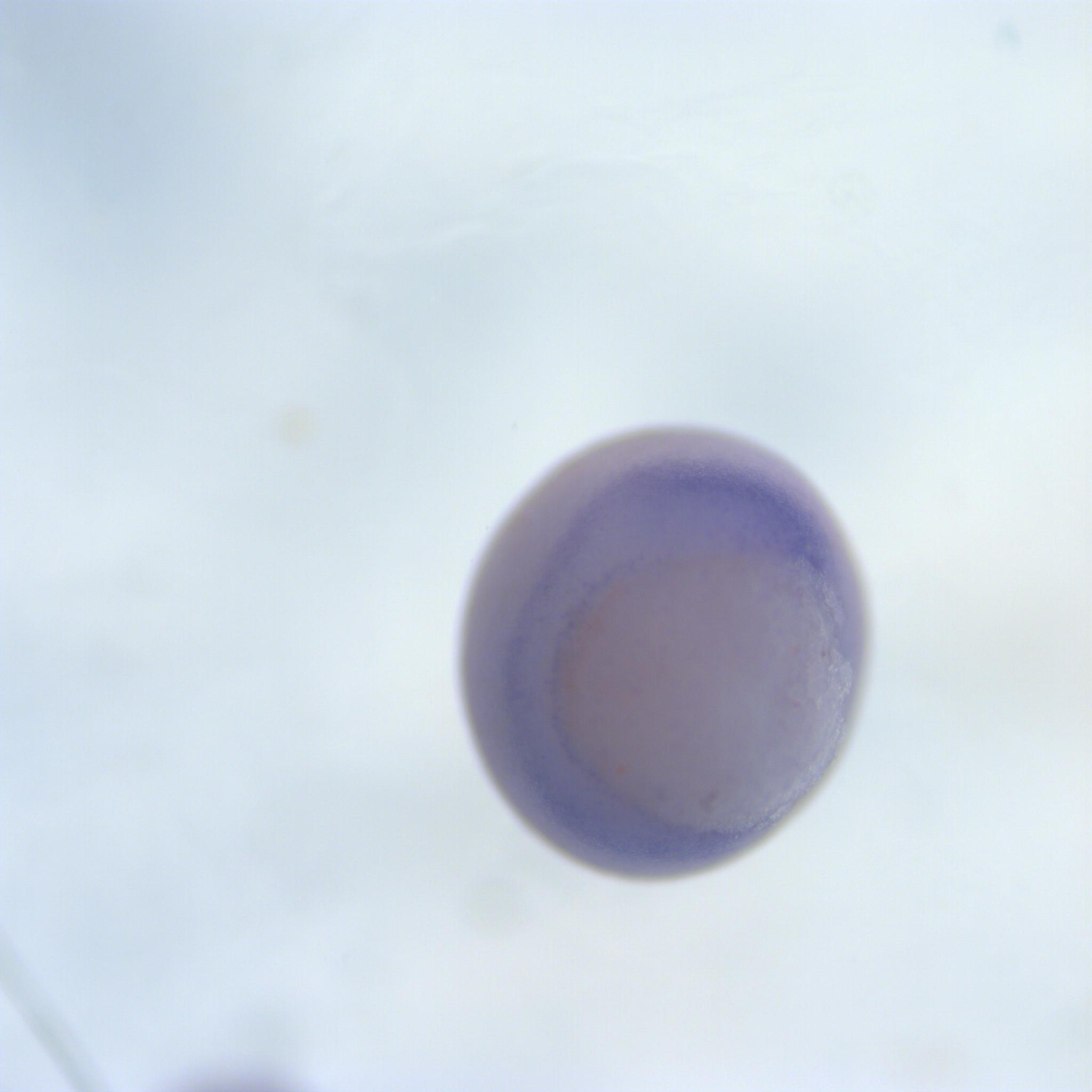

Supplement: Supplementary file 10 — Source data Fig. 6 [file 44319_2025_617_MOESM10_ESM.zip › Figure 6/Figure 6M-Q/Fig 6P_Kremen2 CRISPR/4.tif]

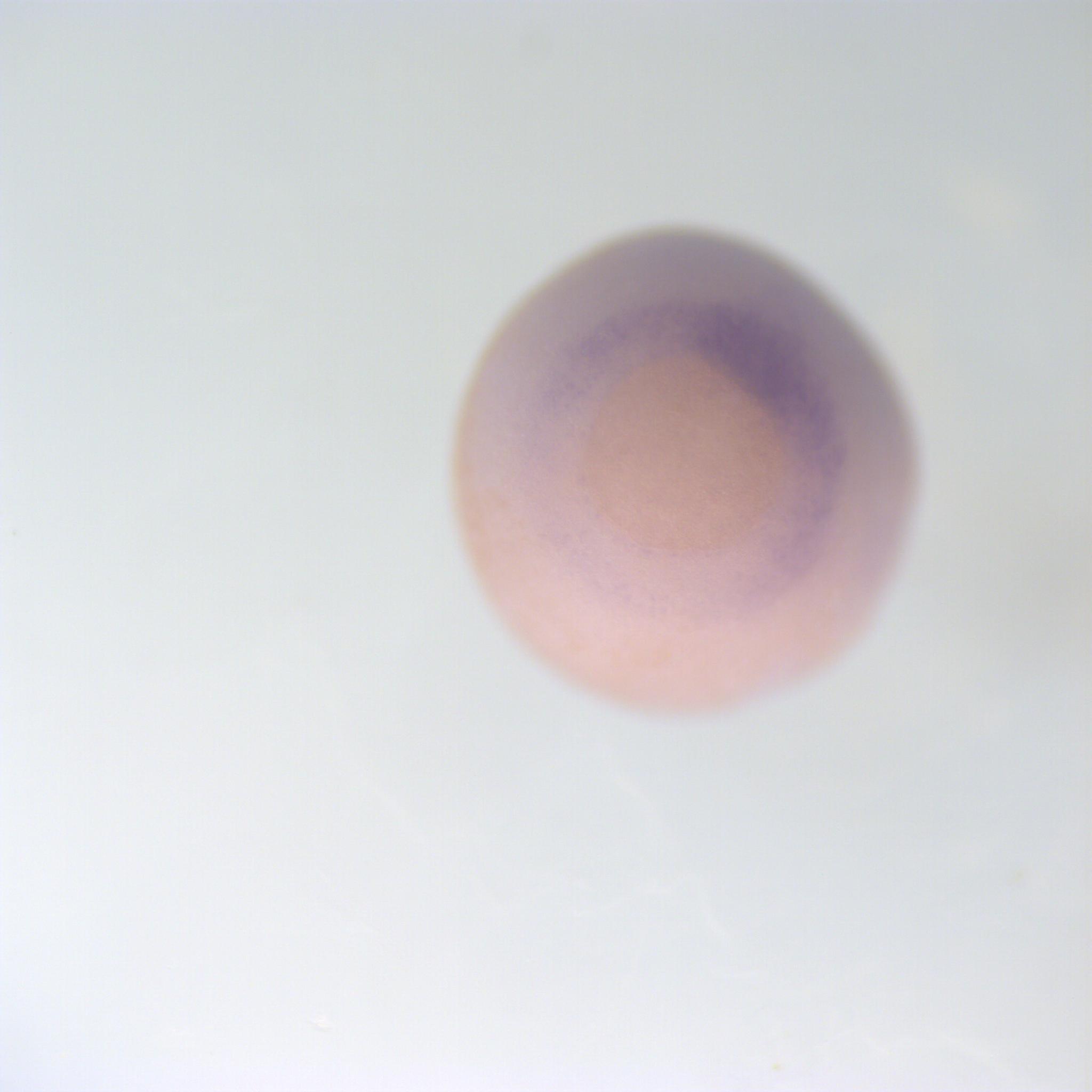

Supplement: Supplementary file 10 — Source data Fig. 6 [file 44319_2025_617_MOESM10_ESM.zip › Figure 6/Figure 6M-Q/Fig 6M_Control/original_1.jpg]

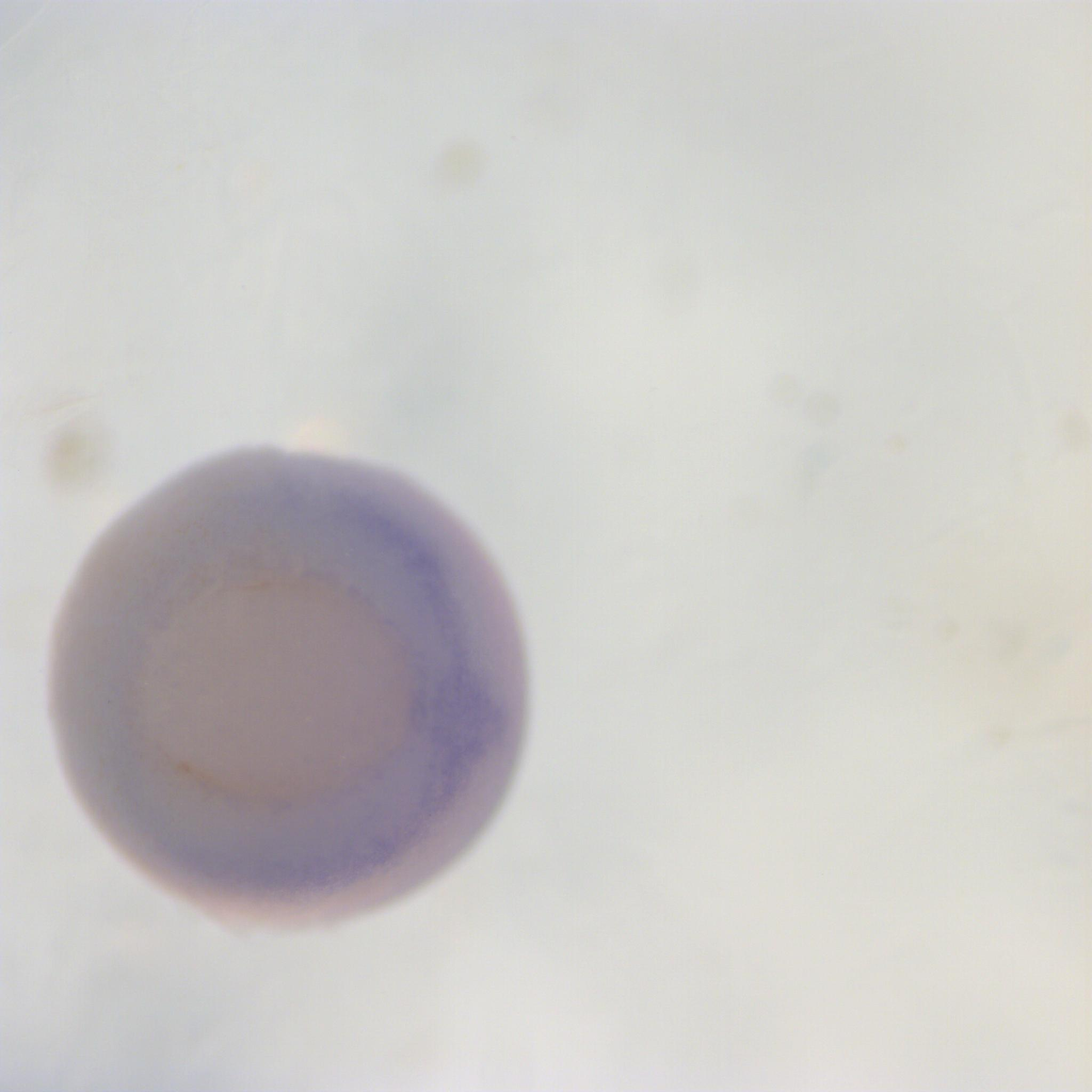

Supplement: Supplementary file 10 — Source data Fig. 6 [file 44319_2025_617_MOESM10_ESM.zip › Figure 6/Figure 6M-Q/Fig 6O_sox8 CRISPR mRNA kremen2/1.3 original.tif]

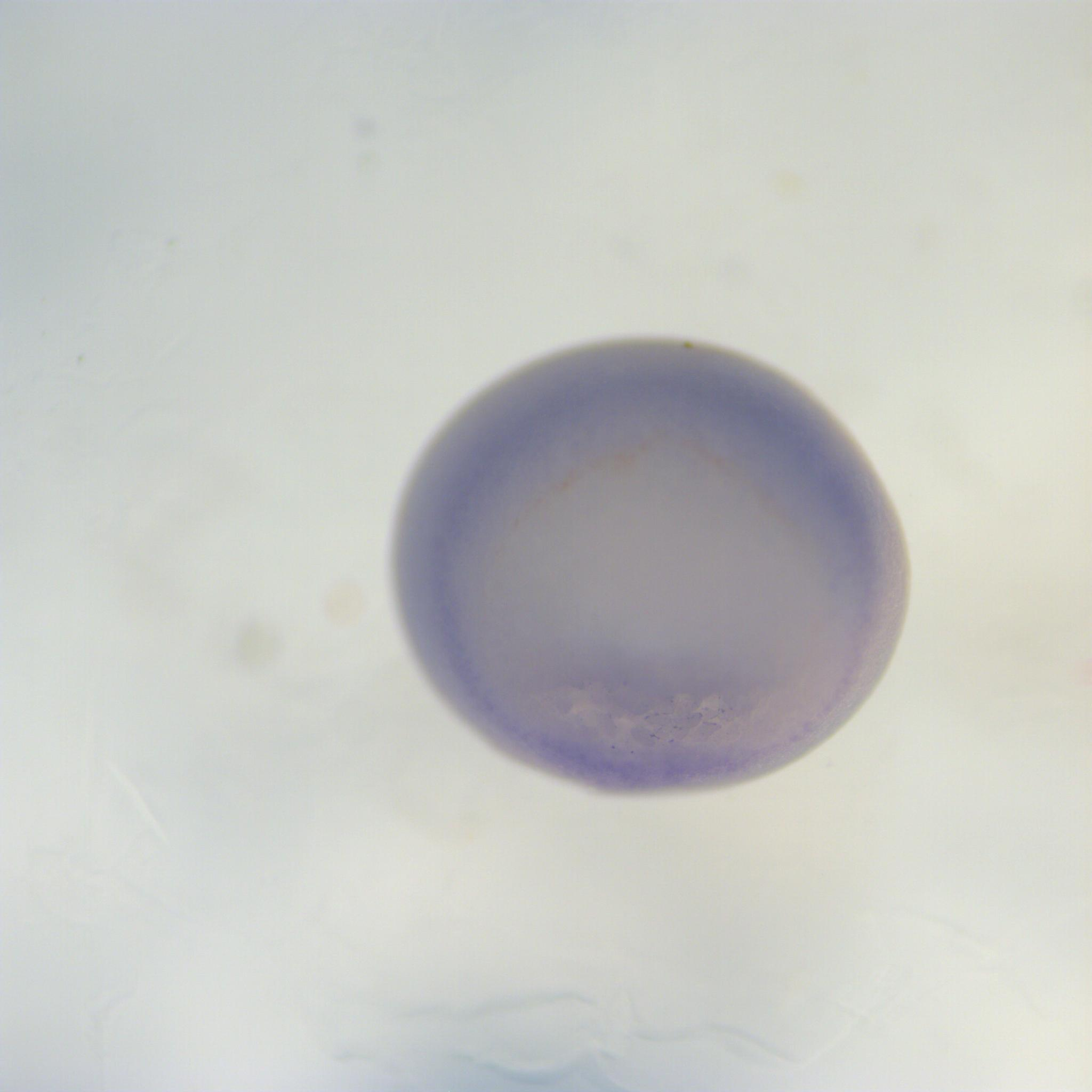

Supplement: Supplementary file 10 — Source data Fig. 6 [file 44319_2025_617_MOESM10_ESM.zip › Figure 6/Figure 6M-Q/Fig 6N_sox8 CRISPR/2.tif]

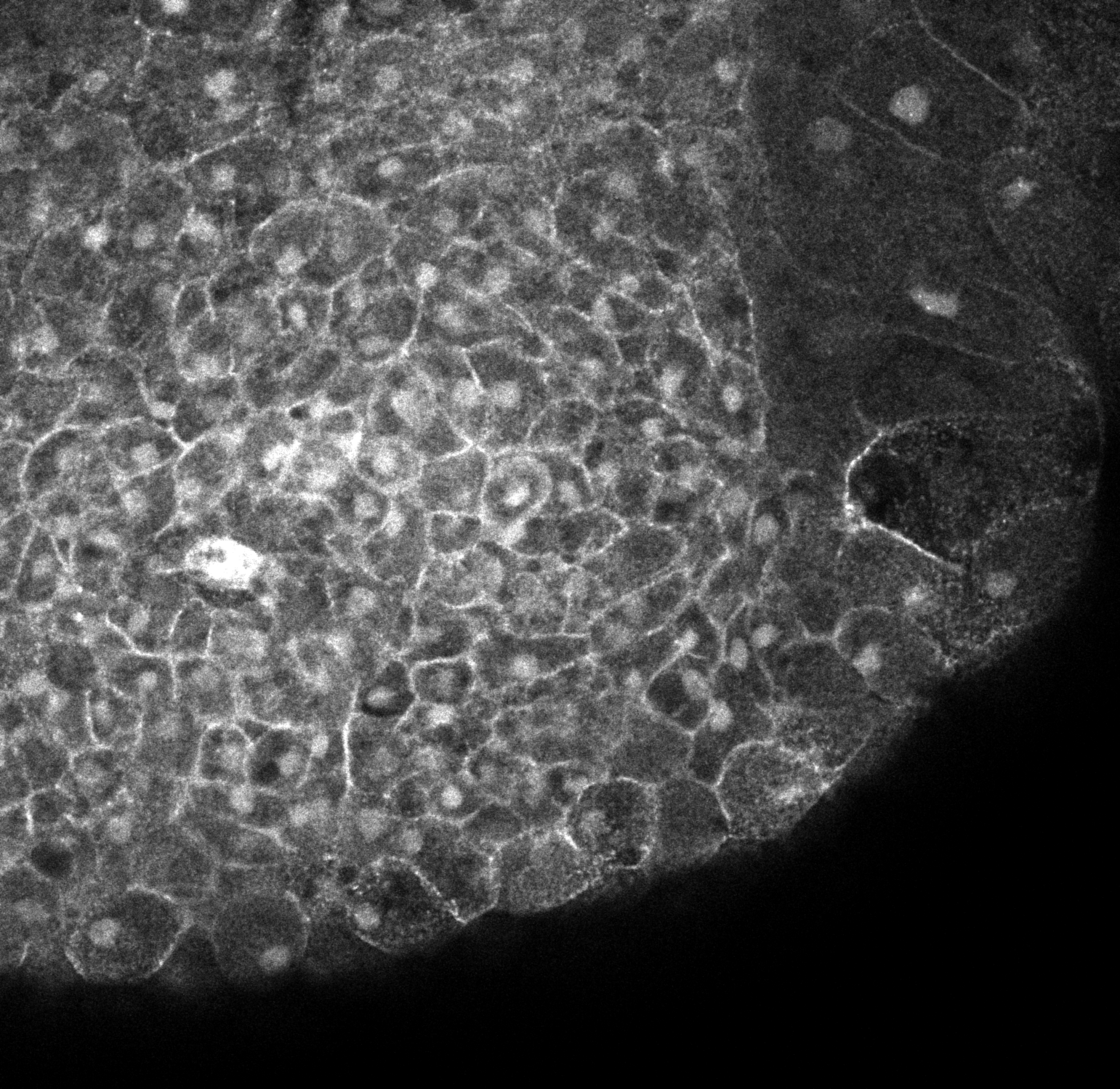

Supplement: Supplementary file 10 — Source data Fig. 6 [file 44319_2025_617_MOESM10_ESM.zip › Figure 6/Figure 6E, F/kremen2 CRISPR/kremen2 CRISPR beta cat_single plan.tif]

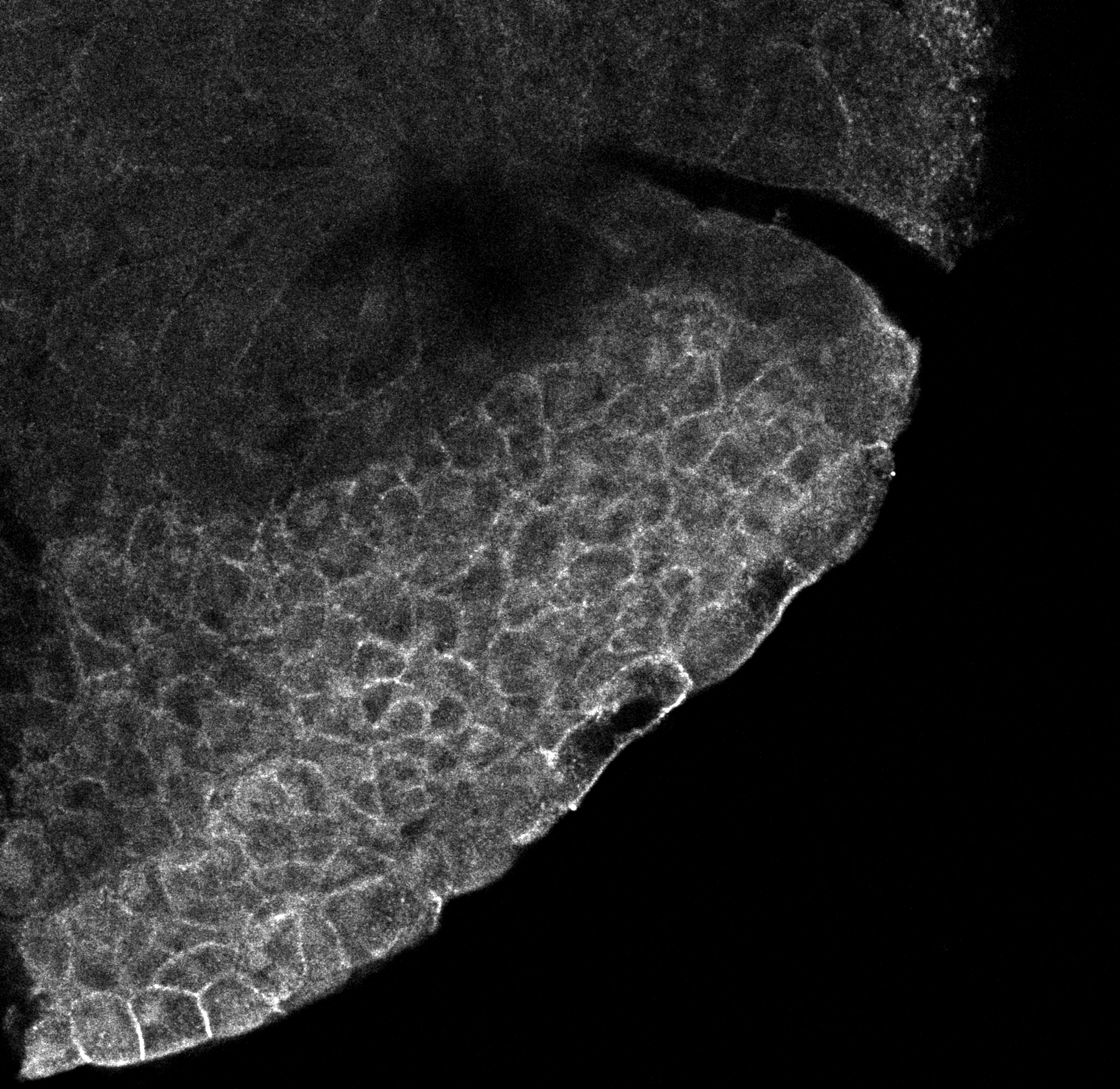

Supplement: Supplementary file 10 — Source data Fig. 6 [file 44319_2025_617_MOESM10_ESM.zip › Figure 6/Figure 6E, F/Control/Control beta cat_single plan.tif]
