# Supplementary material for: Sox8 is essential for vertebrate gastrulation
Source: EMBO Rep. 2025 Nov 10;26(24):6179–208. doi: 10.1038/s44319-025-00617-z (PMC12715262; doi:10.1038/s44319-025-00617-z)
Supplement: Supplementary file 11 — Expanded View Figures [file 44319_2025_617_MOESM11_ESM.pdf]

Expanded View Figures

Figure EV1. *sox8* knockdown by Cas7–11 in the ventrolateral mesoderm impairs embryonic development.

(**A**, **B**) Time-course analysis of the blastopore area shows delayed closure in *sox8* CRISPR embryos compared with controls. Dotted lines delineate the blastopore. Statistical significance was assessed using the two-tailed Mann–Whitney test, ****$P < 0.0001$. Error bars: mean ± SD. (**C**) Representative images of *sox8* CRISPR embryos at the neurula and tailbud stages showing open blastopores (arrow) and shortened anteroposterior axis. Dotted lines delineate the blastopore. (**D**) Percentage of embryos with normal morphology at the neurulation stage. Statistical significance was assessed using a two-sided Fisher's exact test, **** $P < 0.0001$. Error bars represent mean ± SD. $N = 3$ independent experiments ($n = 32$ control, $n = 56$ *sox8* CRISPR Cas7–11). (**E**) Quantification of embryos with normal morphology at the tailbud stage, showing a significant reduction in normal morphology following *sox8* knockdown. Two-sided Fisher's exact test, **** $P < 0.0001$. Error bars represent mean ± SD; $N = 3$ independent experiments ($n = 33$ control, $n = 55$ *sox8* CRISPR). Scale bars: 250 µm.

© The Author(s)

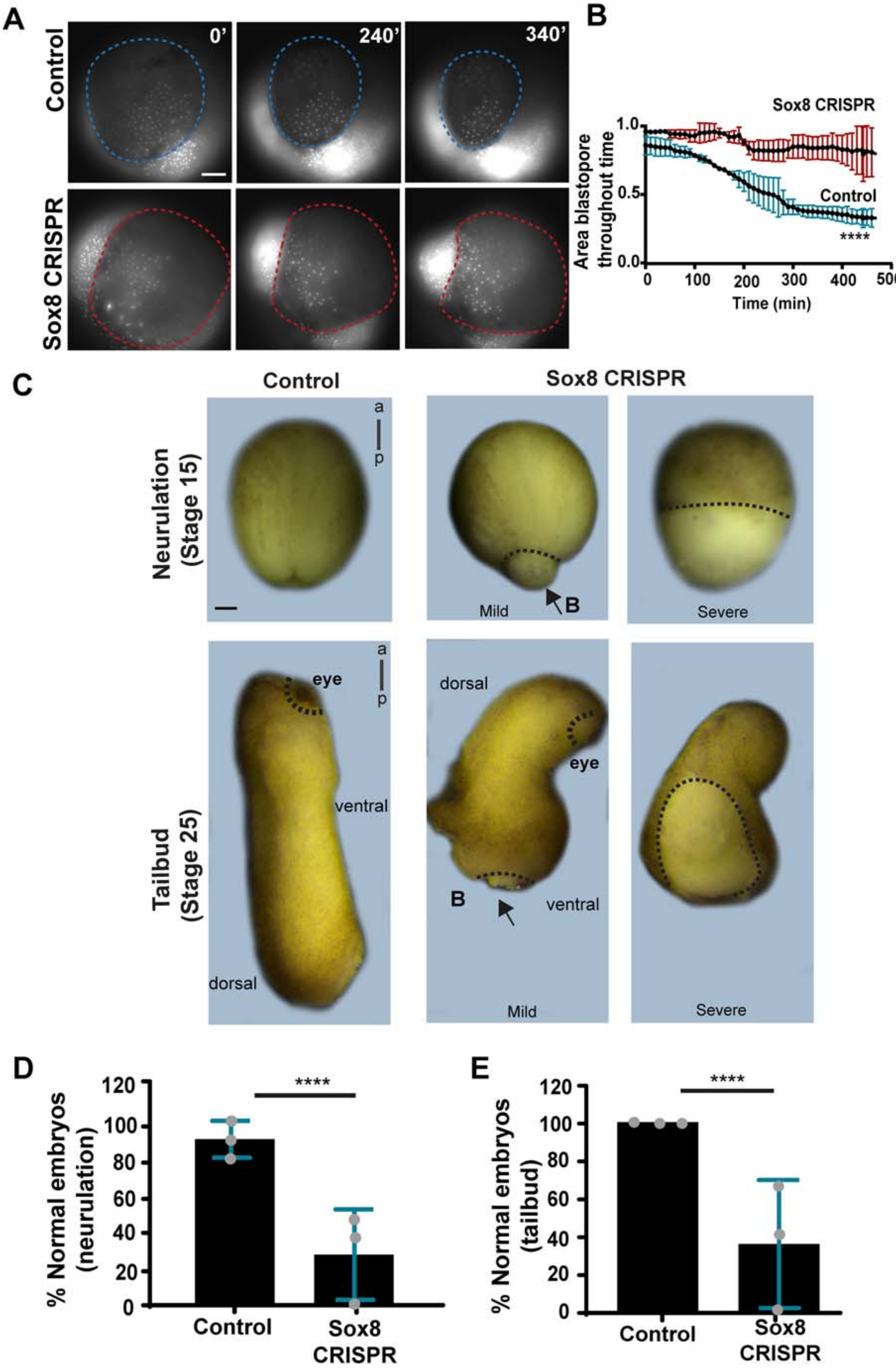
A
Control
Sox8 CRISPR
0'
240'
340'
B
Sox8 CRISPR
Control
Area blastopore throughout time
Time (min)
****
C
Control
Sox8 CRISPR
Neurulation (Stage 15)
Tailbud (Stage 25)
a
p
Mild
Severe
B
eye
dorsal
ventral
D
% Normal embryos (neurulation)
Control
Sox8 CRISPR
E
% Normal embryos (tailbud)

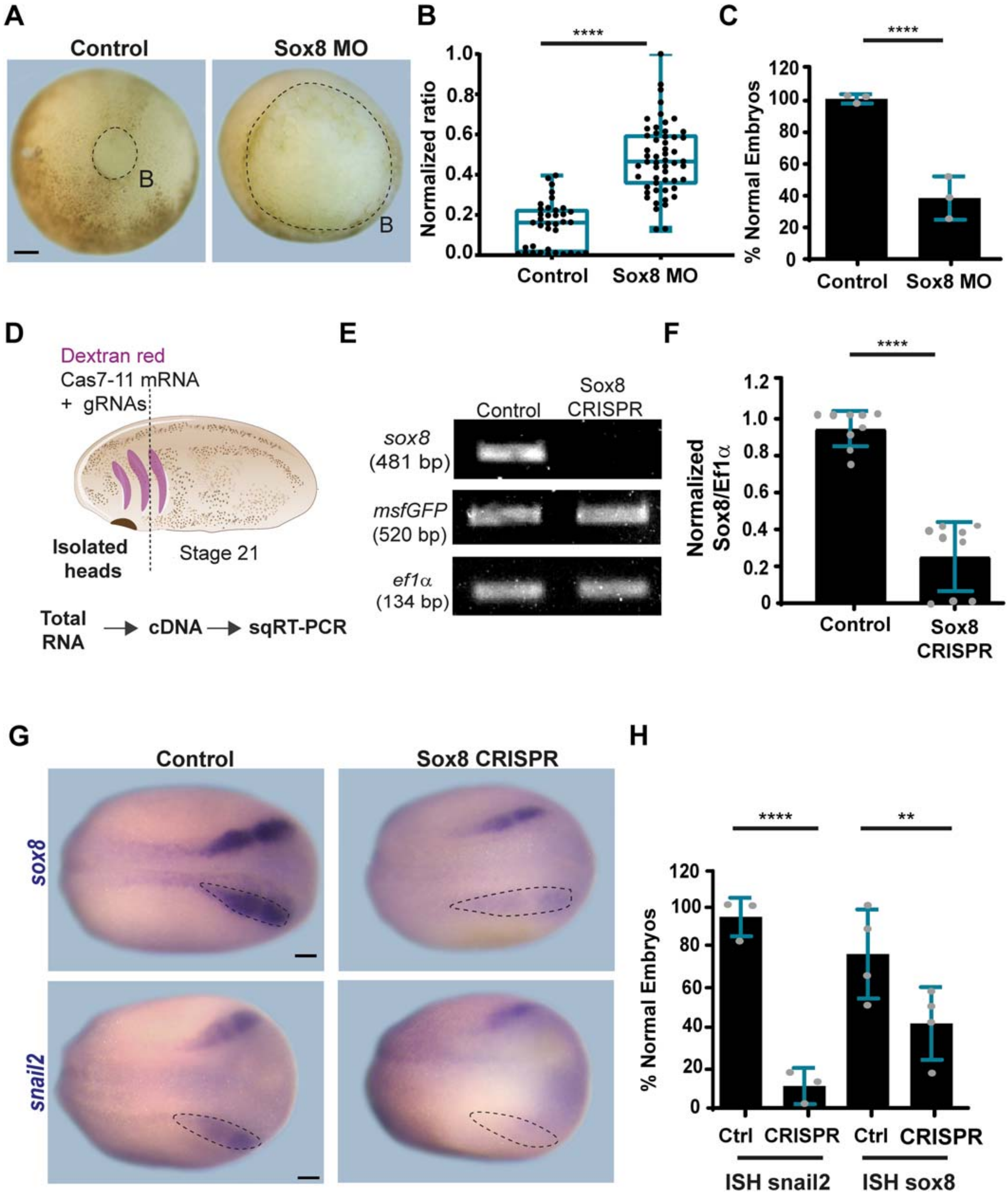
A
Control
Sox8 MO
B
B
B
****
1.0
0.8
0.6
0.4
0.2
0.0
Normalized ratio
Control
Sox8 MO
C
****
120
100
80
60
40
20
0
% Normal Embryos
Control
Sox8 MO
D
Dextran red
Cas7-11 mRNA
+ gRNAs
Isolated heads
Stage 21
Total RNA
cDNA
sqRT-PCR
E
Control
Sox8 CRISPR
sox8 (481 bp)
msfGFP (520 bp)
ef1α (134 bp)
F
****
1.2
1.0
0.8
0.6
0.4
0.2
0
Normalized Sox8/Ef1α
Control
Sox8 CRISPR
G
Control
Sox8 CRISPR
sox8
snail2
H
****
**
120
100
80
60
40
20
0
% Normal Embryos
Ctrl
CRISPR
Ctrl
CRISPR
ISH snail2
ISH sox8

 © The Author(s)

Figure EV2. *sox8* CRISPR recapitulates Sox8 MO phenotypes during the gastrulation and neurula stages.

(**A–C**) Injection of a validated Sox8 morpholino (MO) into the ventrolateral mesoderm induces defects in blastopore closure, as illustrated by representative examples. Dotted lines delineate the blastopore (**B**). (**B**) Box plot showing the normalized ratio of blastopore area to embryo area. Boxes represent the interquartile range (IQR; 25th–75th percentile), with the center line indicating the median and whiskers extending to the minimum and maximum values. Statistical significance was assessed using the two-tailed Mann–Whitney test, $^{****}P < 0.0001$; $N = 3$ independent experiments ($n = 37$ control embryos; n = 54 sox8 MO). Scale bars: 250 µm. (**C**) Percentage of embryos exhibiting normal blastopore size per condition (embryos displaying values below the maximum control ratio value are classified as normal). Two-sided Fisher's exact test, $^{****}P < 0.0001$; Error bars: mean ± SD. $N = 3$ independent experiments ($n = 37$ control; $n = 52$ *sox8* MO). (**D–F**) Semi-quantitative RT–PCR of stage 21 heads (early neurulation), confirming the depletion of *sox8*. The purple stripes represent target injections in the neural crest. Statistical significance was assessed using a two-tailed Mann–Whitney test, $^{****}P < 0.0001$; Error bars: mean ± SD. $N = 3$ independent experiments. (**G**) CRISPR Cas7–11-mediated depletion of *sox8* in the neural crest phenocopies previously reported *sox8* MO phenotypes in the neural crest, such as a delay in neural crest induction (as detected by *ISH* for *snail2*). Dotted lines delineate the neural crest region. (**H**) Quantification of the percentage of embryos with normal signals for *sox8* or *snail2*. Two-sided Fisher's exact test, $^{****}P < 0.0001$; $^{**}P = 0.0033$. Error bars: mean ± SD. $N = 3$ independent experiments; $n = 17$ control embryos (*ISH snail2*); $n = 18$ *sox8* CRISPR Cas7–11 (*ISH snail2*); $n = 38$ control embryos (*ISH sox8*); $n = 41$ *sox8* CRISPR (*ISH Sox8*). Scale bars: 250 µm.

© The Author(s)

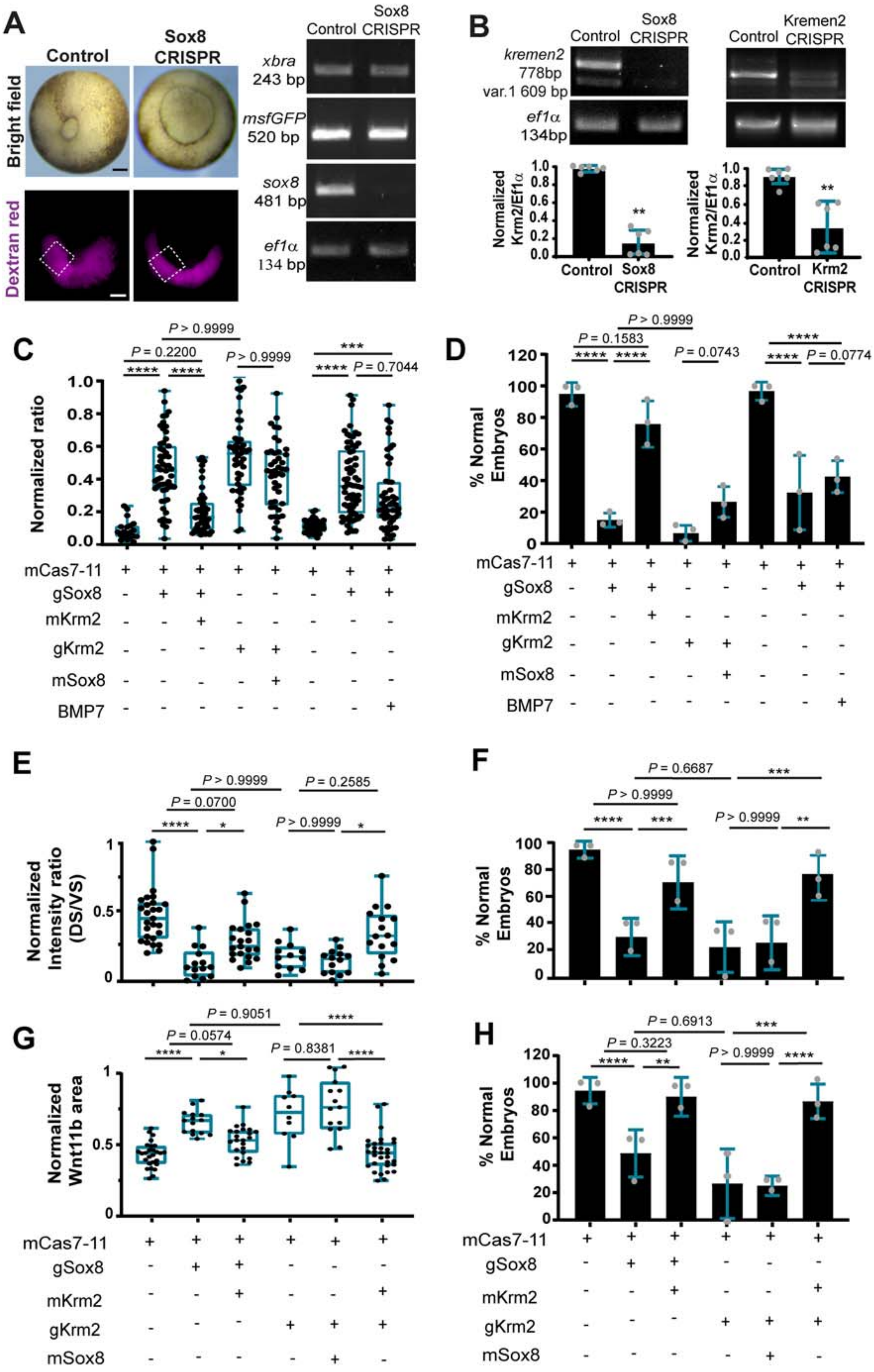
A
Control
Sox8 CRISPR
Bright field
Dextran red
Control Sox8 CRISPR
xbra 243 bp
msfGFP 520 bp
sox8 481 bp
ef1α 134 bp
B
Control Sox8 CRISPR
kremen2 778bp
var.1 609 bp
ef1α 134bp
Control Kremen2 CRISPR
Normalized Krm2/Ef1α
Control Sox8 CRISPR
Control Krm2 CRISPR
C
Normalized ratio
P = 0.2200
P > 0.9999
P = 0.7044
mCas7-11
gSox8
mKrm2
gKrm2
mSox8
BMP7
D
% Normal Embryos
P = 0.1583
P = 0.0743
P = 0.0774
E
Normalized Intensity ratio (DS/VS)
P = 0.0700
P = 0.2585
F
P = 0.6687
G
Normalized Wnt11b area
P = 0.9051
P = 0.0574
P = 0.8381
H
P = 0.6913
P = 0.3223

 © The Author(s)

Figure EV3. *sox8* and *kremen2* downregulation impairs blastopore closure and alters the *wnt11b* expression pattern.

(**A**) SqRT–PCR was performed as a quality control for samples sent for RNA-seq. (**B**) SqRT–PCR and quantification of samples sent for RNA-seq revealed that *kremen2* is downregulated. Statistical significance was assessed by a two-tailed Mann–Whitney test, $^{**}P = 0.0022$. Control for *kremen2* depletion in the context of *kremen2* CRISPR is also shown. Mann–Whitney test, $^{**}P = 0.0022$; Error bars: mean ± SD. (**C**) Box plot showing the normalized ratio of blastopore area to embryos. Statistical significance was assessed by Kruskal–Wallis multiple comparisons test, $^{****}P < 0.0001$; $^{***}P = 0.0002$ (control vs *sox8* CRISPR + *bmp7* mRNA); ns non-significant: $P = 0.2200$ (control vs *sox8* CRISPR + *kremen2* mRNA); $P > 0.9999$ (*sox8* CRISPR vs kremen2 CRISPR); $P > 0.9999$ (*kremen2* CRISPR vs *kremen2* CRISPR + *sox8* mRNA); $P = 0.7044$ (*sox8* CRISPR vs *sox8* CRISPR + *bmp7* mRNA). (**D**) Percentage of embryos exhibiting normal blastopore size per condition (embryos displaying values below the maximum control ratio value are classified as normal). Two-sided Fisher's exact test, $^{****}P < 0.0001$; ns non-significant: $P = 0.0774$ (*sox8* CRISPR vs *sox8* CRISPR + *bmp7* mRNA); $P = 0.1583$ (control vs *sox8* CRISPR + *kremen2*); $P > 0.9999$ (*sox8* CRISPR vs *kremen2* CRISPR); $P = 0.0774$ (*sox8* CRISPR vs *sox8* CRISPR + *bmp7* mRNA); $P = 0.0743$ (*kremen2* CRISPR vs *kremen2* CRISPR + *sox8* mRNA). Error bars: mean ± SD; For (**C**, **D**): $n = 20$ control Cas7-11; $n = 55$ *sox8* CRISPR; $n = 51$ *sox8* CRISPR + *kremen2* mRNA; $n = 45$ *kremen2* CRISPR; $n = 45$ *kremen2* CRISPR + *sox8* mRNA; $n = 39$ control (for rescue with *bmp7* mRNA); $n = 68$ *sox8* CRISPR (for rescue with *bmp7* mRNA); $n = 51$ *sox8* CRISPR + *bmp7* mRNA. $N = 3$ independent experiments. (**E**) Box plot showing the *wnt11b* intensity ratio between dorsal and ventral signals in different conditions. Statistical significance was assessed by Kruskal–Wallis multiple comparisons test, $^{****}P < 0.0001$; $^{*}P = 0.0184$ (*kremen2* CRISPR + *sox8* mRNA vs *kremen2* CRISPR + *kremen2* mRNA); $^{*}P = 0.0472$ (*sox8* CRISPR vs *sox8* CRISPR + *kremen2* mRNA); ns non-significant: $P = 0.0700$ (control vs *sox8* CRISPR + *kremen2* mRNA); $P > 0.9999$ (*kremen2* CRISPR + *kremen2 sox8* mRNA); $P > 0.9999$ (*sox8* CRISPR vs *kremen2* CRISPR); $P = 0.2585$ (*kremen2* CRISPR vs *kremen2* CRISPR + *kremen2* mRNA). (**F**) Percentage of embryos displaying a normal *wnt11b* expression pattern is shown; percentage of normal and abnormal embryos in each condition (embryos displaying higher values than the baseline control intensity ratio value are classified as normal). Two-sided Fisher's exact test, $^{****}P < 0.0001$ (control vs *sox8* CRISPR); $^{***}P = 0.0001$ (*sox8* CRISPR vs *sox8* CRISPR + *kremen2* mRNA); $^{**}P = 0.0092$ (*kremen2* CRISPR + *sox8* mRNA vs *kremen2* CRISPR); $P > 0.9999$ (Control vs *sox8* CRISPR + *kremen2* mRNA); $P = 0.6687$ (*sox8* CRISPR vs *kremen2* CRISPR); $P > 0.9999$ (*kremen2* CRISPR vs *kremen2* CRISPR + *sox8* mRNA). Error bars: mean ± SD. $N = 3$ independent experiments; $n = 27$ control embryos; $n = 14$ sox8 CRISPR; $n = 21$ sox8 CRISPR + kremen2 mRNA; $n = 8$ kremen2 CRISPR; $n = 14$ *kremen2* CRISPR + sox8 mRNA and $n = 15$ kremen2 CRISPR + kremen2 mRNA; (**G**) Box plot showing area of the wnt11b intensity in different conditions. Statistical significance was assessed by ordinary one-way ANOVA, $^{****}P < 0.0001$; $^{*}P = 0.0147$ (*sox8* CRISPR vs sox8 CRISPR + kremen2 mRNA); $P = 0.0574$ (control vs *sox8* CRISPR + *kremen2* mRNA); $P = 0.9051$ (*sox8* CRISPR vs *kremen2* CRISPR); $P = 0.8381$ (*kremen2* CRISPR vs *kremen2* CRISPR + *sox8* mRNA); (**H**) Percentage of embryos displaying a normal *wnt11b* expression pattern is shown; percentage of normal embryos in each condition (embryos displaying lower values than the maximum control area value are classified as normal). Two-sided Fisher's exact test, $^{****}P < 0.0001$; $^{***}P = 0.0004$ (*kremen2* CRISPR + *sox8* mRNA); $^{**}P = 0.0041$ (*sox8* CRISPR vs *sox8* CRISPR + *kremen2* mRNA); $P = 0.3223$ (control vs *sox8* CRISPR + *kremen2* mRNA); $P = 0.6913$ (*sox8* CRISPR vs *kremen2* CRISPR); $P > 0.9999$ (*kremen2* CRISPR + *kremen2* CRISPR + *sox8* mRNA). Error bars: mean ± SD. $n = 27$ control embryos; $n = 15$ *sox8* CRISPR; $n = 23$ *sox8* CRISPR + *kremen2* mRNA; $n = 10$ *kremen2* CRISPR; $n = 15$ *kremen2* CRISPR + *sox8* mRNA and $n = 32$ *kremen2* CRISPR + *kremen2* mRNA (**E**–**H**) $N = 3$ independent experiments; Scale bars: 250 μm. For box plots in (**C**, **E**, **G**), boxes represent the interquartile range (IQR; 25th–75th percentile), with the center line indicating the median and whiskers extending to the minimum and maximum values.

© The Author(s)

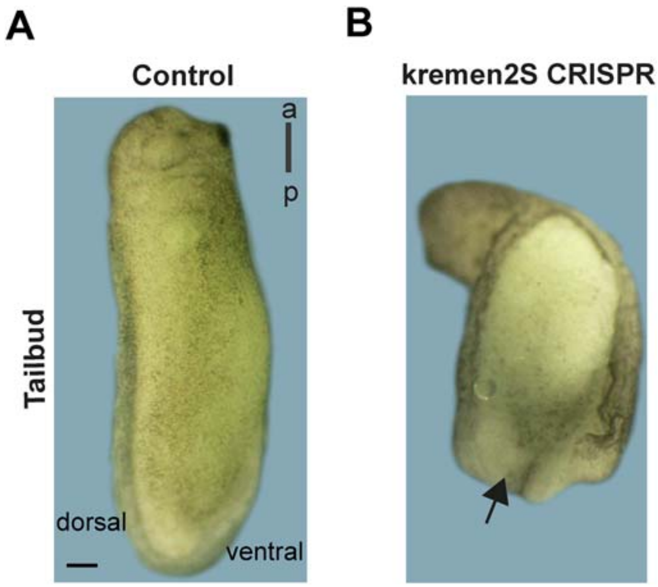

Figure EV4. ***kremen2*** **knockdown by Cas7–11 in the ventrolateral mesoderm impairs embryonic development.**

Representative images of (**A**) control embryos and (**B**) *kremen2* CRISPR embryos at the tailbud stage, showing open blastopores (arrows), with shortened embryos on the anteroposterior axis. Scale bars: 250 µm.

 © The Author(s)

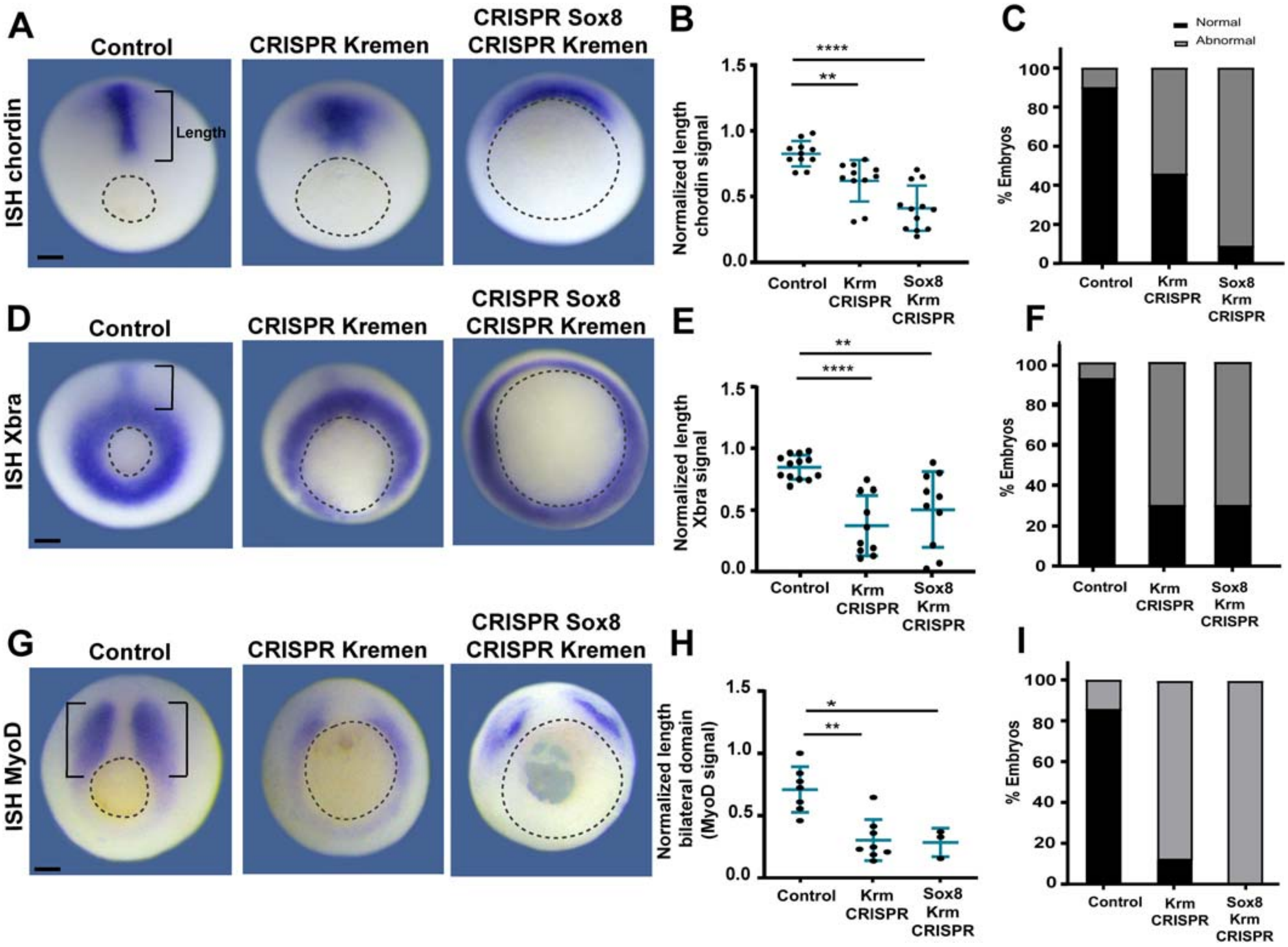

Figure EV5. ***kremen2* CRISPR and *sox8 kremen2* CRISPR double-knockdown disrupt the expression of mesodermal markers.**

(**A**, **D**, **G**) Whole-mount in situ hybridization (ISH) for different markers, *chordin* (**A**), *brachyury* (**D**), and *myoD* (**G**), revealed abnormal expression patterns of these markers in *kremen2* CRISPR and *sox8 kremen2* CRISPR double-knockdown embryos compared with control embryos. Dotted lines delineate the blastopore. (**B**, **E**, **H**) Quantification of marker expression length in the axial mesoderm revealed a significant reduction under CRISPR conditions. (**B**) Ordinary one-way ANOVA, $^{**}P = 0.0081$; $^{****}P < 0.0001$. Error bars: mean ± SD. (**E**) Ordinary one-way ANOVA, $^{**}P = 0.0028$; $^{****}P < 0.0001$. Error bars: mean ± SD. (**H**) Two-tailed Mann–Whitney test, $^{**}P = 0.0022$ (control vs *kremen2* CRISPR) $^{*}P = 0.0167$ (control vs *sox8 kremen2* CRISPR). Error bars: mean ± SD. (**C**, **F**, **I**) Percentages of normal and abnormal embryos in each condition (embryos displaying greater values than the baseline control length value are classified as normal). Sample sizes: ISH chordin: $n = 10$ control; $n = 11$ *kremen2* CRISPR; $n = 12$ *sox8 kremen2* CRISPR; *Xbra* ISH: $n = 13$ control; $n = 10$ *kremen2* CRISPR; $n = 10$ *sox8 kremen2* CRISPR ; *ISH MyoD*: $n = 7$ control; $n = 7$ *kremen2* CRISPR; $n = 3$ *sox8 kremen2* CRISPR. Scale bars: 250 µm.

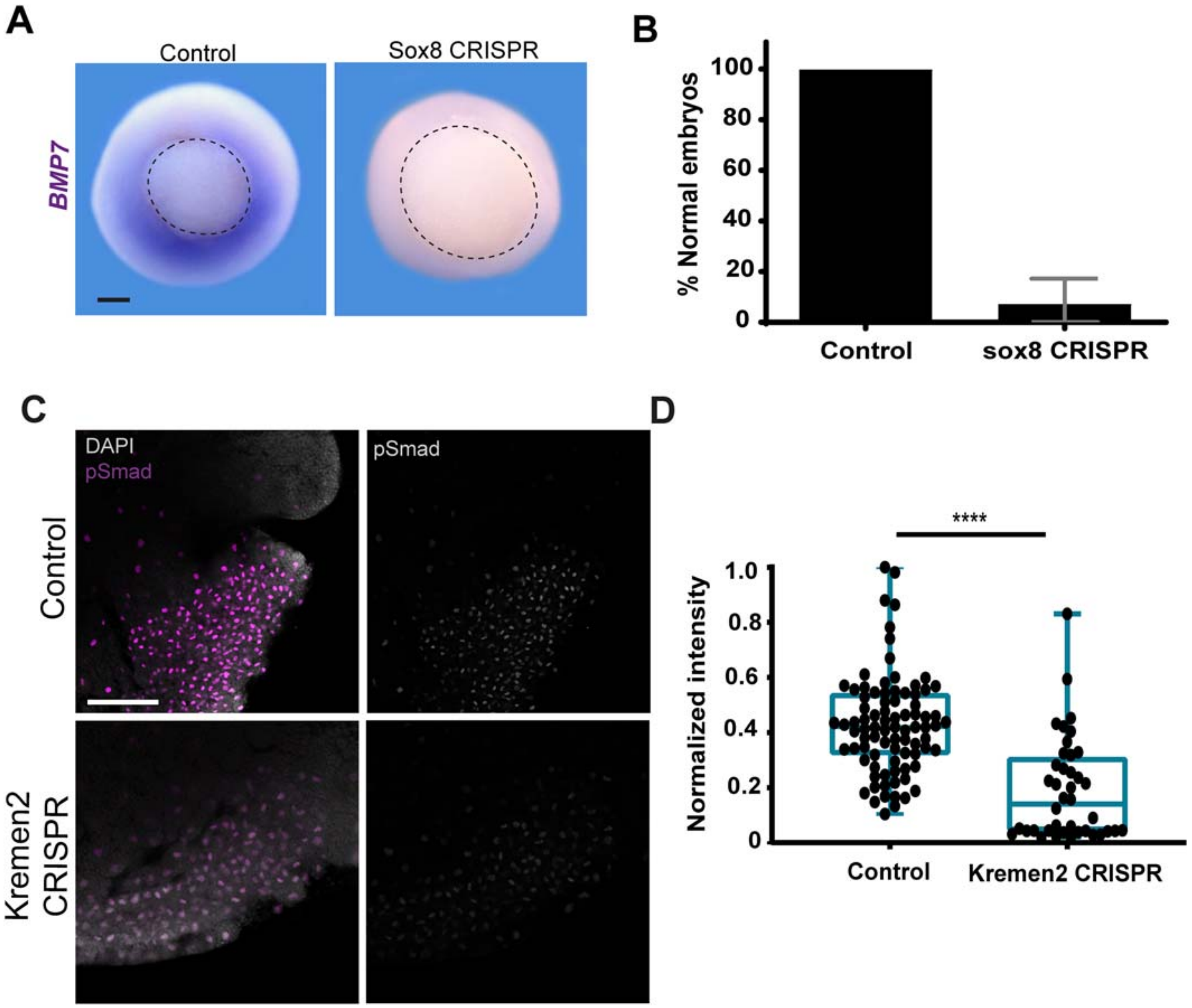

Figure EV6. ***BMP*** **signaling is perturbed under CRISPR conditions.**

(**A**) Whole-mount *ISH* for *bmp7* revealed reduced expression in *sox8* CRISPR embryos. Dotted lines delineate the blastopore. Scale bar: 250 µm. (**B**) Quantification of the percentage of embryos with a normal *bmp7 ISH* signal. *N* = 2 independent experiments (*n* = 5 embryos control; n = 7 embryos *sox8* CRISPR). (**C**) pSmad staining levels are decreased in *kremen2* CRISPR conditions compared with controls. (**D**) Box plot showing the quantification of the nuclear signal in the ventral regions. Boxes represent the interquartile range (IQR; 25th–75th percentile), with the center line indicating the median and whiskers extending to the minimum and maximum values. Statistical significance was assessed by a two-tailed Mann–Whitney test, ****$P < 0.0001$. n = 9 control embryos (85 cells); *n* = 4 *kremen2* CRISPR (40 cells). Scale bar: 100 µm.

© The Author(s)
